# Discovery of a Novel Intron in US10/US11/US12 of HSV-1 Strain 17

**DOI:** 10.3390/v15112144

**Published:** 2023-10-25

**Authors:** Weizhong Chang, Ming Hao, Ju Qiu, Brad T. Sherman, Tomozumi Imamichi

**Affiliations:** Laboratory of Human Retrovirology and Lmmunoinformatics, Frederick National Laboratory for Cancer Research, Frederick, MD 21702, USA; ming.hao@nih.gov (M.H.); jqiumail@gmail.com (J.Q.); bsherman@mail.nih.gov (B.T.S.); timamichi@mail.nih.gov (T.I.)

**Keywords:** novel intron, virus gene, HSV-1, gene annotation, *US10*, *US11*, *US12*

## Abstract

Herpes Simplex Virus type 1 (HSV-1) infects humans and causes a variety of clinical manifestations. Many HSV-1 genomes have been sequenced with high-throughput sequencing technologies and the annotation of these genome sequences heavily relies on the known genes in reference strains. Consequently, the accuracy of reference strain annotation is critical for future research and treatment of HSV-1 infection. In this study, we analyzed RNA-Seq data of HSV-1 from NCBI databases and discovered a novel intron in the overlapping coding sequence (CDS) of *US10* and *US11*, and the 3′ UTR of *US12* in strain 17, a commonly used HSV-1 reference strain. To comprehensively understand the shared *US10*/*US11*/*US12* intron structure, we used *US11* as a representative and surveyed all *US11* gene sequences from the NCBI nt/nr database. A total of 193 high-quality *US11* sequences were obtained, of which 186 sequences have a domain of uninterrupted tandemly repeated RXP (Arg-X-Pro) in the C-terminus half of the protein. In total, 97 of the 186 sequences encode US11 protein with the same length of the mature US11 in strain 17:26 of them have the same structure of *US11* and can be spliced as in strain 17; 71 of them have transcripts that are the same as mature *US11* mRNA in strain 17. In total, 76 *US11* gene sequences have either canonical or known noncanonical intron border sequences and may be spliced like strain 17 and obtain mature *US11* CDS with the same length. If not spliced, they will have extra RXP repeats. A tandemly repeated RXP domain was proposed to be essential for US11 to bind with RNA and other host factors. US10 protein sequences from the same strains have also been studied. The results of this study show that even a frequently used reference organism may have errors in widely used databases. This study provides accurate annotation of the *US10*, *US11*, and *US12* gene structure, which will build a more solid foundation to study expression regulation of the function of these genes.

## 1. Introduction

Herpes Simplex Virus type 1 (HSV-1) is a double-stranded DNA (~152 Kb) virus belonging to the *Alphaherpesvirinae* subfamily of the *Orthoherpesviridae* family [1,2]. HSV-1 infects humans and causes a variety of clinical manifestations, including neonatal herpes, corneal blindness, herpetic whitlow, meningitis, encephalitis, and genital herpes [3,4]. With the advance of high-throughput sequencing technologies, an increased number of HSV-1 strains have been sequenced. The annotation of these genome sequences heavily relies on the known genes in reference strains. Therefore, the accuracy of reference strain annotation is critical for the field. Strain 17 is a commonly used HSV-1 reference strain, which has 56 protein coding genes in the long unique region (UL) [5,6], 2 protein coding genes in the two inverted long repeated regions (TRL and IRL) [6,7], 12 protein coding genes in the short unique region (US) [6,8], and 1 protein coding gene in the two inverted short repeat regions (IRS and TRS) [6,9].

Next Generation Sequencing (NGS) technologies have accelerated the advances in all biological and medical research in the past two decades [10,11,12,13]. RNA-sequencing (RNA-Seq) uses NGS to determine the presence of all RNA molecules and their quality in a biological sample and directly compares these results between experiments [14]. RNA-Seq, using the Illumina platform, has been used to study the repertoire of human fibroblast circular RNAs associated with cellular responses to HSV-1 strain 17 infection [15]. Using the data from this study, we evaluated the transcripts annotated in the HSV-1 strain 17 genome sequence and discovered a 27 nt novel intron in overlapping coding regions in *US10* and *US11*, and the 3′ UTR of *US12*. The results were further validated with poly-A RNA-Seq data using the PacBio platform from another group [16,17]. The genome sequences in the same region of other HSV-1 strain genomes were evaluated to obtain the full picture of this region in circulating HSV-1 strains. Although the function of *US10* is unclear [18], *US11* has been studied and reported by several groups during the past several decades. *US11* encodes a late-expressed, double-stranded RNA binding protein [19,20]. It is a virion component and associates with the 60S subunit of ribosomes [20,21]. Like HIV (human immunodeficiency virus) Rev and HLTV-1 (human T-lymphotropic virus type 1) Rex, US11 is involved in post-transcriptional regulation of gene expression [22]. US11 interacts with protein kinase R (PKR), blocks the phosphorylation of eIF-2alpha by activated PKR and prevents the cessation of protein synthesis [23]. US11 also interacts with other host factors, including Nucleolin, which is involved in US11 trafficking [24]; PAT1, which is important for the intracellular movement of viral components [25]; and Nucleophosmin, which is implicated in multiple stages of viral infection for different viruses [26]. In virus-infected cells, the US11 protein drastically reduces the formation of autophagosomes by disrupting the TRIM23-TBK1 Complex, which regulates the innate immune response in the infected cells [27]. An accurate HSV-1 *US10*/*US11*/*US12* structure and annotation will provide a more solid foundation to study expression regulation and the function of these genes and may help to control HSV-1 infection.

## 2. Materials and Methods

### 2.1. Virus Genome Sequence and RNA-Seq Data

All data used in this study were downloaded from NCBI. HSV-1 genome sequences from GenBank: strain 17 (NC_001806.2), F (GU734771.1), McKrae (MN136524.1) and KOS (JQ780693.1), whole genome sequencing data of HSV-1 strain 17 (NCBI SRA #: ERR3278848). RNA-Seq data from SRA using SRA-Tools (v.3.0.3): Illumina total RNA-Seq data of HSV-1 strain 17-infected human fibroblast KMB17 cells (NCBI SRA #: SRR6029569, SRR6029570, SRR6029571) [15], strain F, McKrae and KOS infected neuron (NCBI SRA #: SRR10885165 and SRR10885180 for strain F, SRR10885168 and SRR10885184 for McKrae, SRR10885177 and SRR10885186 for KOS) [28]; PacBio poly-A RNA-Seq data of HSV-1 strain 17-infected Vero cell culture (NCBI BioProject #: PRJNA382882) [16,17].

### 2.2. Analysis of US10/US11/US12 Gene and Protein

To study the transcriptome of HSV-1, the Illumina RNA-Seq sequencing reads were quality trimmed with Trimmomatic [29] with a quality score > 30 within a sliding window size of 20 and minimum length of 100. The trimmed reads were then aligned to virus genome sequences using STAR (V. 2.7.10b) [30] and indexed with Samtools [31]. For the PacBio poly-A RNA-Seq data, the sequencing reads were aligned to the genome sequence using minimap2 (v2.24) [32]; the SAM file was then converted to BAM, and sorted and indexed with Samtools. The indexed alignment was visualized and investigated using IGV (V. 2.13.2).

Since the novel intron is shared by US10/US11/US12, we only need to use one as a representative to understand the intron structure. US11 sequences were obtained by BLAST searching the NCBI nr/nt database with the coding region of the HSV-1 strain 17 US11 gene (searched on 28 June 2023). Duplicated sequences of the same strains, the sequence of recombinant genomes and the sequences containing ambiguous bases (N), which indicated a low quality of the sequence, were removed. The remaining sequences were grouped based on the lengths of *US11* nucleotide sequences of its coding region. The sequences in the same group were then aligned with a multiple sequence aligner, Clustal Omega (V.2.1) [33], in the EBI website (https://www.ebi.ac.uk/Tools/msa/clustalo/, accessed April–September 2023). The US10 protein sequences in the same strains were retrieved from GenBank and followed by the same analyses. The US12 protein structure was not studied because the novel intron is located in the 3′ UTR of US12.

### 2.3. Phylogenetic Analysis of HSV-1 Strains with Different US11 Gene Length

To analyze the relationship between HSV-1 strains with different *US10*/*US11*/*US12* gene lengths, we performed a phylogenetic analysis as previously described [34]. Briefly, we used the genome sequences with TRL (~9 K) and TRS (~7 K) trimmed to perform the phylogenetic analysis since TRL and IRL, and TRS and IRS, are two pairs of inverted repeat sequences. These trimmed non-redundant sequences were aligned using ClustalW with standard parameters (v2.1) [35]. The alignment file was then converted to mega format with the MEGAX software package (v11.0.10) [36]. A maximum-likelihood tree was generated using MEGAX software, with the general time-reversible nucleotide substitution model with 5 gamma categories, 1000 bootstrap replicates, and complete deletion of alignment gaps, giving a total of 125,368 positions in the data set.

## 3. Results

### 3.1. Discovery of Novel Intron in US10/US11/US12 of HSV-1 Strain 17

**Figure 1:**
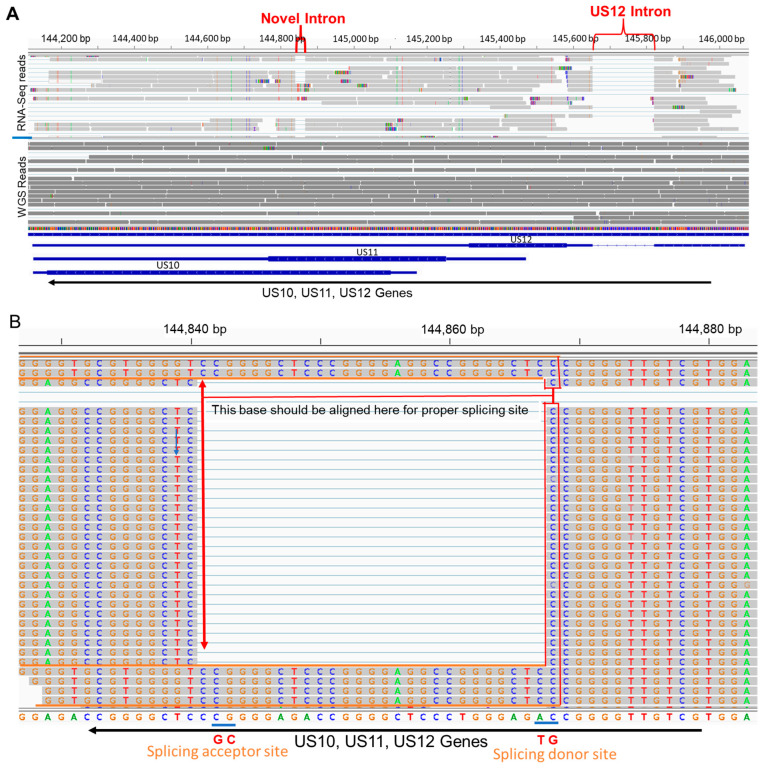
Identification of a novel intron in *US10*/*US11*/*US12* genes of HSV-1 strain 17: (**A**) the RNA-Seq sequencing reads from an HSV-1 strain 17-infected human fibroblast KMB17 cell culture sample (NCBI SRA #: SRR6029571) and the genome sequencing reads of HSV-1 strain 17 BAC (NCBI SRA #: ERR3278848) were aligned with the reference sequence, HSV-1 strain 17 genome sequence (NCBI AC #: NC_001806.2), and are shown with light gray and dark gray color, respectively. The novel intron in *US10*/*US11*/*US12* and well-known *US12* intron are marked with a red bracket. The annotated gene structure of *US10*, *US11*, and *US12* in the reference is shown at the bottom of the panel. Thick bar: coding sequence, narrow bar: untranslated exon sequence, line: intron. These genes are on the complement strand of the reference sequence. (**B**) A zoomed-in view of the region of a newly identified novel intron. After we move base “C” on right side of gap to left side, the splicing donor site GT (complement of AC) and acceptor site CG (complement of CG) are shown at the border of the novel intron. Some read sequences within the intron could be moved properly as shown in the figure, consistent with the intron structure. *US12* intron zoomed-in view is shown in Figure S1.

**Figure 2:**
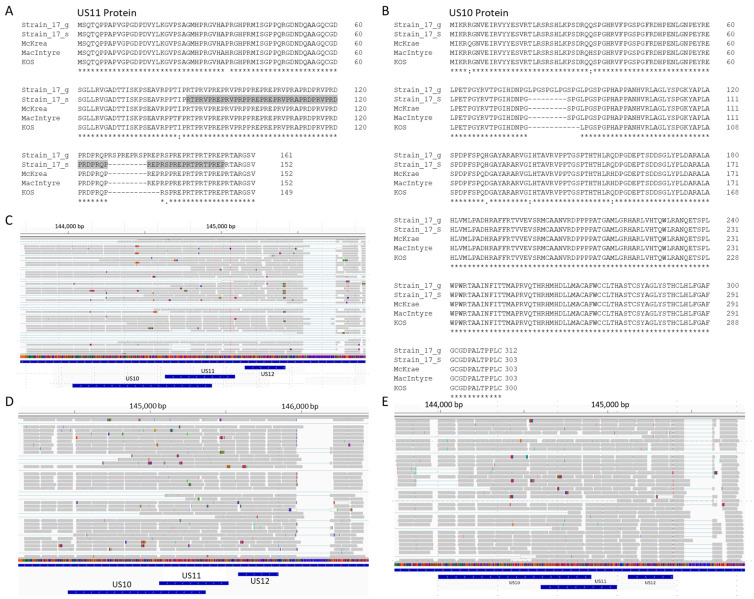
No new intron identified in *US10*/*US11*/*US12* genes in HSV-1 strain F, McKrae, and KOS. Alignment of annotated US11 (**A**) and US10 (**B**) protein sequence in HSV-1 strain 17 in reference sequence (Strain_17_g, NCBI ACC #: NC_001806.2) and mature form with 27 nt intron spliced out (Strain_17_s), together with the annotated *US11* and *US10* from HSV-1 strain F, McKrae, MacItyre, and KOS. (**C**) The alignment of the RNA-Seq sequencing reads from an HSV-1 strain F-infected human neuron cell culture sample (NCBI SRA #: SRR10885165) with its genome sequence (NCBI ACC #: GU734771.1). (**D**) The alignment of the RNA-Seq sequencing reads from the HSV-1 strain McKrae-infected human neuron cell culture sample (NCBI SRA #: SRR10885168) with its genome sequence (NCBI ACC #: MN136524.1). (**E**) The alignment of the RNA-Seq sequencing reads from the HSV-1 strain KOS-infected human neuron cell culture sample (NCBI SRA #: SRR10885177) with its DNA genome sequence (NCBI ACC #: JQ780693.1). The blue bars at the bottom of panels (**C**–**E**) are the ORFs of *US10*, *US11*, and *US12*. * annotate the homologous position based on the alignment.

To evaluate the annotation of the genome sequence, the Illumina total RNA-Seq sequencing reads from HSV-1 strain 17-infected fibroblast KMB14 were obtained from NCBI (NCBI SRA #: SRR6029571), quality trimmed with Trimmomatic (V 0.39) [29], and aligned with the HSV-1 strain 17 genome sequence (NCBI Accession #: NC_001806.2) using STAR (V 2.7.10b) [37]. The alignments were visualized and evaluated using IGV together with the annotation file. A 27 nt gap was present (upper part in Figure 1A) in the overlapped *US10* and *US11* coding sequence, which also overlapped with the 3′ UTR of *US12*. The genome sequence used here can be validated by aligning with the Illumina genome sequencing reads (NCBI SRA #: ERR3278848) (lower part of Figure 1A). With proper shifting of bases of RNA-Seq sequencing reads aligned to the gap region, it becomes clear that this 27 nt gap is a complete intron structure with the border sequence GT–CG (Figure 1B). The intron boundary sequence GT-CG was previously observed [38,39]. The well-known intron in US12 [40,41,42] was observed in the same alignment (upper part of Figure 1A and Figure S1), which validated that the sequencing reads are RNA-Seq reads. The novel intron in *US10*/*US11*/*US12* was further verified in two other samples, NCBI SRA #: SRR6029569 (Figure S2A,B) and SRR6029570 (Figure S2C,D), showing the reproducibility of the results. We could not find intron-unprocessed RNAs of *US10*/*US11*/*US12* in these RNA-Seq sequencing reads with total RNA. Most likely, the portion of unprocessed pre-mRNA is too small and needs higher depth to be detected. We further validated our novel discovery with the data from a different platform: combined RNA-Seq sequencing reads with the PacBio SMRT platform from six HSV-1 strain 17-infected Vero cell culture samples (NCBI BioProject #: PRJNA382882) were aligned with the same HSV-1 strain 17 genome sequence. We could identify the same newly discovered intron structure for *US10*, *US11*, and *US12* (Figure S3). With this novel intron spliced (Strain_17_s), the HSV-1 strain 17 US10 and US11 protein have the same length as other laboratory HSV-1 strains, including F, McKrae, and MacIntyre (303 aa for US10 and 152 aa for US11); is nine amino acids shorter than the US10 and US11 annotated in GenBank (NCBI Accession: NC_001806.2, Strain_17_g); and is three amino acids longer than strain KOS (300 for US10 and 149 aa for US11) (Figure 2A,B). The alignment of publicly available RNA-Seq sequencing reads of strain F (NCBI SRA #: SRR10885165 and SRR10885180), McKrae (NCBI SRA #: SRR10885168 and SRR10885184), and KOS (NCBI SRA #: SRR10885177 and SRR10885186) to their genomic sequences (NCBI ACC #: F, GU734771.1; McKrae, MN136524.1; and KOS, JQ780693.1) showed that these strains also contain the known intron within the *US12* transcript [40,41,42], but do not have the intron in the *US10*/*US11*/*US12* overlapping region (Figure 2C–E and Figure S4A–C).

### 3.2. Survey of the Intron Region of US11/US10/US12 in HSV-1 Strains

**Figure 3:**
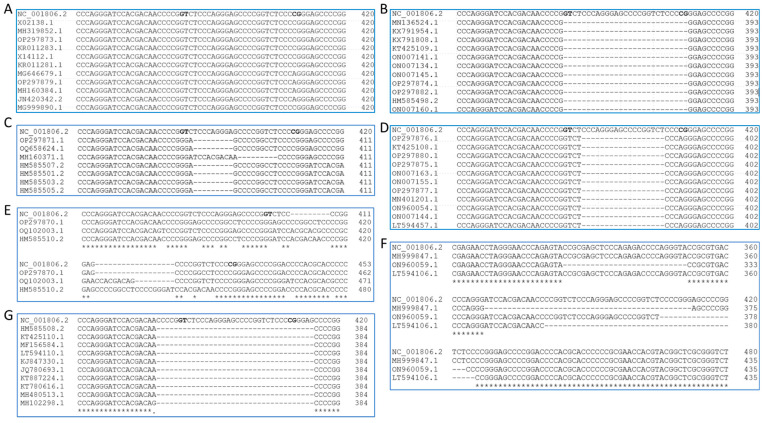
The alignment of *US11* gene sequences with different lengths compared to the *US11* gene in strain 17. (**A**) The same length; (**B**) 27 nt shorter, the same length of mature HSV-1 *US11* in HSV-1 strain 17; (**C**) 9 nt shorter; (**D**) 18 nt shorter; (**E**) 9 nt, 18 nt, and 27 nt longer; (**F**) 45 nt shorter; (**G**) 36 nt shorter. Splicing donor and acceptor sites are noted with bold font in US11 sequence of strain 17. Bold letters (GT, CG) represent intron border sequences; * represent the homologous position based on the alignment.

At the nucleotide level, the intron structure for US10, US11, and US12 is identical. Therefore, we used the US11 sequence to comprehensively survey the structure around the newly discovered intron. A total of 408 full-length US11 sequences were obtained by BLAST searching the NCBI nr/nt database with the HSV-1 strain 17 genome sequence of the US11 coding region (searched on 28 June 2023). In total, 241 of these remained after removing duplicated sequences of the same strains or recombinant genomes and 193 sequences were left after removing the sequences containing ambiguous bases (Ns), which indicated low quality. More than half of the sequences were the same length as the HSV-1 strain 17 genome sequence covering US11 coding sequences (486 bp, 26 strains; Figure 3A, Supplemental Data S1) or the same length as the mature HSV-1 strain 17 US11 coding sequence (459 bp, 71 strains; Figure 3B, Supplemental Data S2). Alignment of the 26 US11 genomic sequences of length 486 bp (Supplemental Data S1) showed 9 of them with canonical intron border sequences GT-AG [43] and 17 of them with known noncanonical intron border sequences: 15 with GT-CG [38,39] and 1 with GG-CA [43]. The remaining one GG-CG had known 5′ splicing donor site GG [44] and 3′ splicing acceptor site CG [38,39]. Therefore, the 27 nt sequence within the US11 gene in these strains can be spliced out like strain 17, and they form the same length of mature US11 protein (152 aa) as strain 17.

Most of the remaining *US11* gene sequences of the other strains are also a multiple of 9 nt different from the HSV-1 strain 17 *US11* gene: 7 sequences are 9 nt shorter (Figure 3C, Supplemental Data S3); 66 sequences are 18 nt shorter (Figure 3D, Supplemental Data S4); 3 sequences are 9 nt (GenBank ACC #: OP297870.1), 18 nt (GenBank ACC #: OQ102003.1), and 27 nt (GenBank ACC #: HM585510.2) longer than HSV-1 strain 17 US11 (Figure 3E, Supplemental Data S5), respectively. All of these sequences (76 total) have either canonical or noncanonical intron donor and acceptor sequences. Therefore, mature US11 in these strains can possibly have the same length as that of US11 in strain 17. However, we do not have access to transcript data for these strains to evaluate this possibility. *US11* in 13 strains is shorter than mature *US11* in HSV-1 strain 17: 10 sequences are 36 nt shorter than the *US11* gene in strain 17 including the frequently used strain KOS (Figure 3G, JQ780693.1) and 3 sequences are 45 nt shorter than the *US11* gene in strain 17 (Figure 3F).

Only seven sequences were not a multiple of 9 nt different from US11 of strain 17. Three are 21 nt and one is 24 nt shorter than the US11 gene of strain 17 (Supplemental Data S6), and three sequences are not a multiple of 3 nt (22, 23, and 25) shorter compared with the HSV-1 strain 17 US11 gene (Supplemental Data S7). US11 in these three strains will have a frameshift at the C-terminus. It is worth noting that these three genome sequences contain many ambiguous nucleotides, indicating that these non-multiples of three nt deletions may be due to sequencing errors.

In summary, 186 out of 193 HSV-1 US11 sequences surveyed have either the same mature length as the coding sequence of US11 in strain 17 or a multiple of 9 nt difference from the coding sequence of US11 in strain 17. All of these differences are around the region aligned to the novel intron and are present in US10 and US12.

### 3.3. HSV-1 US11 and US10 Protein Structure

**Figure 4:**
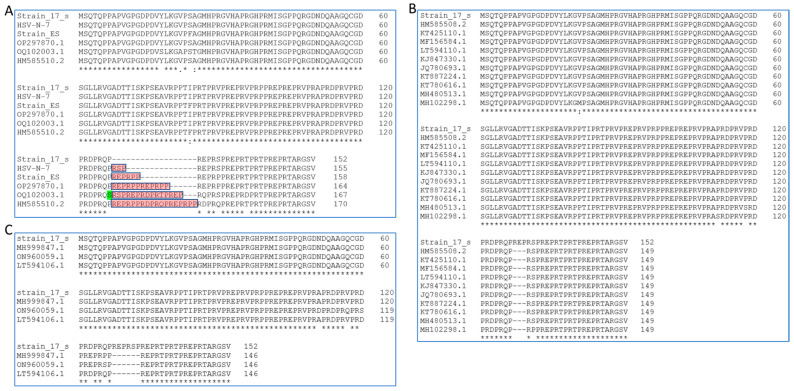
The alignment of US11 amino acid sequences with different lengths compared to mature US11 protein in strain 17. (**A**) Alignment of the US11 amino acid sequence of different lengths with mature US11 in HSV-1 strain 17: HSV-N-7 (representative of 18-nt-shorter group), ES (representative of 9-nt-shorter group), OP297870.1 (9 nt longer), OQ102003.1 (18 nt longer), and HM585510.2 (27 nt longer). The peptide shaded with red represents the intron region if the introns were not spliced. If the proposed introns were spliced, these peptide sequences would not be present and US11 protein is of the same length as US11 in HSV-1 strain 17. (**B**) Alignment of the US11 amino acid sequences of 36-nt-shorter group with mature US11 in HSV-1 strain 17. Amino acids in red represent peptide translated from the proposed intron region if the intron is not spliced out. Green “S” annotated Serion occupies Proline position in RXP repeat. (**C**) Alignment of the US11 amino acid sequences of 45-nt-shorter group with mature US11 in HSV-1 strain 17. * represents homologous position based on the alignment.

As described above, *US11* in more than half of the strains that either have the 27 nt novel intron or the same length of the mature *US11* in strain 17 (Figure 3A,B) will have the US11 protein of 152 aa (Figure 2A). There are a total of 20 tandem RXP repeats located in the C-terminus half of the US11 protein strain 17, as reported by Rixon and McGeoch [45]. The only difference is that they had three extra RXP repeats from the intron, which was unknown at the time. *US10* in these strains will also have the same length (Figure 2B). As described above, 76 of these *US11* gene sequences are longer than mature *US11* in strain 17 and have canonical or known noncanonical splicing sites (Figure 3C–E). If spliced, the US11 protein in these strains may have the same length of mature US11 in strain 17. If these sequences are not spliced, the retained sequence would be translated to one or multiple copies of RXP, and the tandemly repeated RXP in these US11 proteins would still be uninterrupted (Figure 4A) except for strain OQ102003.1, which had a Serine in the Proline position (highlighted in green). Since *US11* genes with a length that is 9 or 18 nt shorter than that of *US11* in strain 17 have the same sequence within the intron splicing border, we used strain ES (NCBI ACC #: OQ658624) and HSV-N-7 (NCBI ACC #: KY922719.1) to represent each group in this protein alignment (Figure 4A and Figure S5A). Ten *US11* genes, which are 36 nt shorter than the *US11* gene in strain 17 (Figure 3G), have one RXP repeat less than the mature US11 protein in strain 17 (Figure 4B). Although 45 nt deletions in three *US11* genes are aligned to different locations of the *US11* gene of strain 17 (Figure 3F), the two RXP deletions are aligned to the same location due to the tandem repeat feature (Figure 4C). In summary, the uninterrupted RXP repeat domain might be essential for the function of US11, but its length is not stringent.

The proposed intron sequences within *US11* in different strains are located in the overlapping coding sequence of *US10* and *US11*. *US10* and *US11* are in different reading frames and *US10* has several short PGX (Proline-Glycine-X) repeat regions. If the proposed non-27-nt introns could not be spliced, the US10 protein would retain an extra peptide in the proposed intron regions (Figure S5A). The *US10* sequences with a length 9 nt and 18 nt shorter than *US10* in HSV-1 strain 17 represented by ES (NCBI ACC #: OQ658624) and HSV-N-7 (NCBI ACC #: KY922719.1) would have an extra six amino acids (PGLPGS) or three amino acids (PGL) in the short tandem PGX repeat domain if the intron could not be spliced. The *US10* sequences with a length 36 nt shorter than *US10* in HSV-1 strain 17 represented by KOS (NCBI ACC #: JQ780693.1) had lost a PGS repeat in the short tandem PGX repeat domain (Figure S5B). We noticed that US10 proteins in three strains with a length 45 nt shorter than US11 in strain 17 (HSV-H12118—NCBI ACC #: MH999847.1, 1974-HTZ—NCBI ACC #: ON960059.1, and 2158_2007—NCBI ACC #: LT594106.1) have significantly different sequences around the region aligned to the newly discovered intron in *US10* in strain 17 (Figure S5C), suggesting that the region is flexible for US10 function. For the three strains that have a longer proposed *US10*/*US11*/*US12* intron than strain 17 (OP297870.1, OQ102003.1, and HM585510.2), US10 would also retain a significantly different peptide sequence if the intron could not be spliced (Figure S5A).

### 3.4. Evolution of HSV-1 with Different Length of US10/US11 Gene

**Figure 5:**
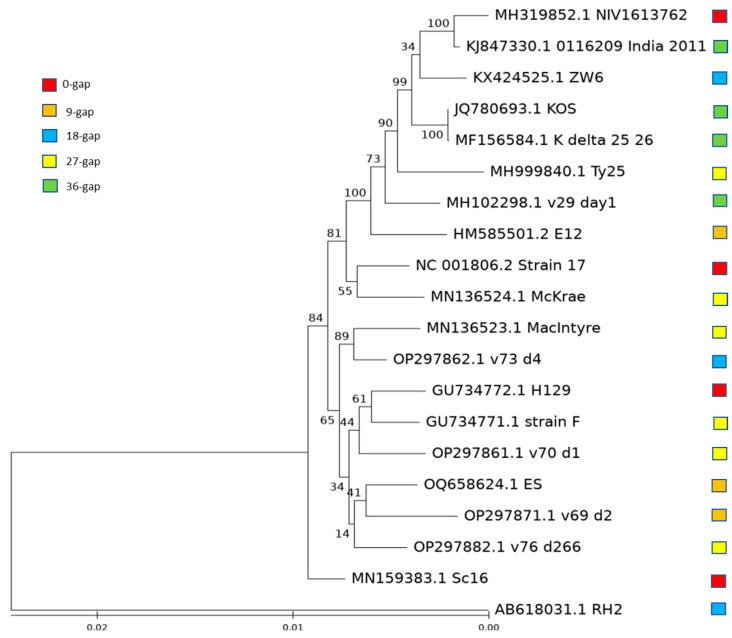
Phylogenetic analysis of HSV-1 genome sequences from the groups with different *US10*/*US11* gene length. Red square: same length of *US10*/*US11* gene in strain 17; brown square: 9 nt shorter than *US10*/*US11* gene in strain 17; blue square: 18 nt shorter than *US10*/*US11* gene in strain 17; yellow square: 27 nt shorter than *US10*/*US11* gene in strain 17; green square: 36 nt shorter than *US10*/*US11* gene in strain 17.

To determine if the strains with different lengths of *US10* and *US11* genes have an evolutionary relationship based on the length, we performed a phylogenetic analysis with 20 strains including 5 strains commonly used in HSV-1 research as references: strains 17, F, McKrae, MacIntyre, and KOS, and 15 randomly selected strains with different lengths: 3 from the groups with common strains and 4 from the groups that do not have commonly used strains, to ensure that every group has a balanced number of strains. The phylogenetic tree shows that the strains with the same *US10*/*US11* gene length were not necessarily clustered together (Figure 5), suggesting that strains with the same *US10*/*US11* gene length evolved independently.

## 4. Discussion

In this work, we discovered a 27 nt novel intron in commonly used HSV-1 reference strain 17, which is located within an overlapping CDS of *US10*/*US11* and the 3′ UTR of *US12*, using data from the publicly available NCBI databases. The intron is located within the tandem RXP repeat region in *US11* with the 27 nt intron representing 3 RXP repeats in the annotation from GenBank in NCBI (NC_001806.2). A total of 20 RXP repeats can be found from position 86 to 145 in mature *US11* of strain 17 (Figure 2A). This region is critical for US11 to bind with RNA and associate with ribosomes [20]. It is also the region interacting with PKR [23], Nucleolin [24], PAT1 [25], and Nucleophosmin [26].

A total of 193 *US11* gene sequences were surveyed in this study, revealing different lengths of *US11*. A total of 71 strains have *US11* gene sequences with the same length of mature HSV-1 strain 17 *US11* without the intron (Figure 3B, Supplemental Data S2). A total of 26 strains have gene sequences with the same length and structure of *US11* in strain 17 and can be spliced as *US11* in strain 17 (Figure 3A, Supplemental Data S1). Together, more than half of the investigated strains (97 out of 193) have the same US11 protein structure (152 aa). A total of 89 out of the remaining *US11* genome sequences are a multiple of 9 nt different from HSV-*1* strain 17 US11. A total of 76 of them are longer than mature HSV-1 *US11* in strain 17 (Figure 3C–E, and Supplemental Data S3–S5). All 76 of these *US11* gene sequences have either canonical or known noncanonical intron donor and acceptor sequences. Therefore, they could have a mature US11 protein of the same length as the strain 17 mature US11 protein, which needs to be further investigated by researchers with access to those strains. If these *US11* sequences cannot be spliced, they would have longer lengths than US11 in strain 17 with extra RXP repeats (Figure 4A, shaded with red). Thirteen *US11* gene sequences are shorter than the mature US11 in HSV-1 strain 17. Of these, 10 sequences are 36 nt shorter than the US11 gene in strain 17 (Figure 3G) and 3 amino acids (one RXP) shorter than mature *US11* in strain 17, like strain KOS (Figure 4B); 3 sequences are 45 nt shorter than the *US11* gene in strain 17 (Figure 3F) and 6 amino acids (two RXP) shorter than mature US11 in strain 17 (Figure 4C). Only seven sequences were not a multiple of 9 nt different from US11 of strain 17. A total of 186 out of 193 surveyed US11 amino sequences have uninterrupted RXP repeats, suggesting its possible role in the function of US11. It is proposed that the tandem RXP repeat can form poly-L-proline II helices, which have a strong electrostatic polarity [20,46]. The ring atoms of the proline residues form the uncharged and relatively hydrophobic face. The side chains of the repeating arginine residues form the second, highly positively charged face, which may stabilize binding to RNA by electrostatic interactions with phosphates. The third face is formed with a variety of hydrophobic, uncharged polar, and acidic side chains, which can provide the specificity of binding by US11 [20]. Poly-L-proline II helices are not stabilized with intrachain hydrogen bonds, which is consistent with our predicted structure of mature US11 protein in HSV-1 strain 17 using secondary and tertiary structural prediction tools (Supplemental Methods, Figure S6).

In contrast, the difference in US10 from three strains that are 45 nt shorter than the *US10* gene sequence of strain 17 is significantly different in the nearby region from the strain 17 mature US10 protein because the *US10* and *US11* genes are in different reading frames (Figure S4C), suggesting that the region is not important for the function of US10. In fact, we could not find any substantial reports on the function of US10. For the three strains, which have longer *US10* gene sequences than that of strain 17, the US10 protein sequences are the same as mature US10 in strain 17 if the proposed intron was spliced. They would be significantly different with the proposed intron from the strain 17 mature US10 if the proposed intron cannot be spliced (Figure S5A, shaded with red in OP297870.1, OQ102003.1, and HM58551.2).

As described above, *US10*/*US11* in HSV-1 strains have different lengths of gene sequences. Most of them could have mature *US10* and *US11* of the same length as that of strain 17. We found that not all strains with the same gene lengths evolved from the same ancestor strains using a phylogenetic analysis (Figure 5). How this novel intron is acquired or lost in *US10*/*US11*/*US12* and the effect of the introns with different lengths on the regulation of gene expression need to be further investigated. On the other hand, the RXP tandem repeat might be a strict restraint for HSV-1 evolution and 9 nt is the insertion/deletion module in the corresponding region of the intron we discovered in strain 17.

The organization of *US10*, *US11*, and *US12* in the HSV-1 genome is very interesting: they start from a different position but end at the same position. More interestingly, US12 has a very long 3′ UTR, which covers the overlapping full *US10* and *US11* coding sequences [47]. Since this intron is located within the 3′ UTR of *US12,* we do not expect any effect on the function of US12. However, the 3′ UTR has been proven to play important roles in the regulation of mRNA including stability, localization, and translation of the mRNA [48]. The function of the 3′ UTR of *US12* and how the intron affects this function need to be further investigated.

## 5. Conclusions

In summary, we discovered a novel intron in the overlapping coding sequences of *US10* and *US11*, which overlaps the 3′ UTR of *US12* in the frequently used HSV-1 reference strain 17. The intron structure remains intact in many other strains and these strains have mature *US10* and *US11* of the same length. This study shows that even sequence information of frequently used model organisms can contain errors and accumulated publicly available data can be useful to correct them. The correction of *US10*/*US11*/*US12* transcript annotation in HSV-1 strains offers the opportunity to study the function of this intron in the regulation of these genes and could help to better understand HSV-1 infection and its treatment in the future.

## Data Availability

All data are acquired from NCBI and EBI using the information in the Materials and Methods section.

## Supplementary Materials

The following supporting information can be downloaded at: https://www.mdpi.com/article/10.3390/v15112144/s1, Supplemental Materials: Supplemental Methods with seven references [49,50,51,52,53,54,55], and Figures; Supplemental Data S1: Alignment of the US11 Gene with the Same Gene Length of US11 Gene in HSV-1 Strain 17; Supplemental Data S2: Alignment of the HSV-1 US11 Gene without Intron; Supplemental Data S3: Alignment of the HSV-1 US11 Gene 9 nt Shorter Than HSV-1 Strain 17; Supplemental Data S4: Alignment of the HSV-1 US11 Gene 18 nt Shorter Than HSV-1 Strain 17; Supplemental Data S5: Alignment of the HSV-1 US11 Gene with the Length of Multiple-9-nt Longer Than HSV-1 Strain 17; Supplemental Data S6: Alignment of the HSV-1 US11 Gene Non-Multiple-9-nt shorter Than US11 Gene in HSV-1 Strain 17; Supplemental Data S7: Alignment of the HSV-1 US11 Gene Non-Multiple-3-nt shorter Than US11 Gene in HSV-1 Strain 17.
