# Supplementary material for: Discovery of a Novel Intron in US10/US11/US12 of HSV-1 Strain 17
Source: Viruses. 2023 Oct 25;15(11):2144. doi: 10.3390/v15112144 (PMC10675037; doi:10.3390/v15112144)
Supplement: Supplementary file 1 [file viruses-15-02144-s001.zip › Supplemental Data S1. 0-nt alignment.pdf]

Supplemental Data S1: Alignment of the US11 Gene with the Same Gene Length of US11 Gene in HSV-1 Strain 17

```
NC_001806.2      ATGAGCCAGACCCAACCCCCGGCCCCAGTTGGGCCGGGCGACCCAGATGTTTACTTAAAA         60
X02138.1         ATGAGCCAGACCCAACCCCCGGCCCCAGTTGGGCCGGGCGACCCAGATGTTTACTTAAAA         60
MH319852.1       ATGAGCCAGACCCAACCCCCGGCCCCAGTTGGGCCGGGCGACCCAGATGTTTACTTAAAA         60
OP297873.1       ATGAGCCAGACCCAACCCCCGGCCCCAGTTGGGCCGGGCGACCCAGATGTTTACTTAAAA         60
KR011283.1       ATGAGCCAGACCCAACCCCCGGCCCCAGTTGGGCCGGGCGACCCAGATGTTTACTTAAAA         60
X14112.1         ATGAGCCAGACCCAACCCCCGGCCCCAGTTGGGCCGGGCGACCCAGATGTTTACTTAAAA         60
KR011281.1       ATGAGCCAGACCCAACCCCCGGCCCCAGTTGGGCCGGGCGACCCAGATGTTTACTTAAAA         60
MG646679.1       ATGAGCCAGACCCAACCCCCGGCCCCAGTTGGGCCGGGCGACCCAGATGTTTACTTAAAA         60
OP297879.1       ATGAGCCAGACCCAACCCCCGGCCCCAGTTGGGCCGGGCGACCCAGATGTTTACTTAAAA         60
MH160384.1       ATGAGCCAGACCCAACCCCCGGCCCCAGTTGGGCCGGGCGACCCAGATGTTTACTTAAAA         60
JN420342.2       ATGAGCCAGACCCAACCCCCGGCCCCAGTTGGGCCGGGCGACCCAGATGTTTACTTAAAA         60
MG999890.1       ATGAGCCAGACCCAACCCCCGGCCCCAGTTGGGCCGGGCGACCCAGATGTTTACTTAAAA         60
MG999850.1       ATGAGCCAGACCCAACCCCCGGCCCCAGTTGGGCCGGGCGACCCAGATGTTTACTTAAAA         60
MH999848.1       ATGAGCCAGACCCAACCCCCGGCCCCAGTTGGGCCGGGCGACCCAGATGTTTACTTAAAA         60
MH160390.1       ATGAGCCAGACCCAACCCCCGGCCCCAGTTGGGCCGGGCGACCCAGATGTTTACTTAAAA         60
GU734772.1       ATGAGCCAGACCCAACCCCCGGCCCCAGTTGGGTCGGGCGACCCAGATGTTTACTTAAAA         60
MG999839.1       ATGAGCCAGACCCAACCCCCGGCCCCAGTTGGGCCGGGCGACCCAGATGTTTACTTAAAA         60
JN420341.1       ATGAGCCAGACCCAACCCCCGGCCCCAGTTGGGCCGGGCGACCCAGATGTTTACTTAAAA         60
JN400093.1       ATGAGCCAGACCCAACCCCCGGCCCCAGTTGGGCCGGGCGACCCAGATGTTTACTTAAAA         60
MG999872.1       ATGAGCCAGACCCAACCCCCGGCCCCAGTTGGGCCGGGCGACCCAGATGTTTACTTAAAA         60
JN420340.1       ATGAGCCAGACCCAACCCCCGGCCCCAGTTGGGCCGGGCGACCCAGATGTTTACTTAAAA         60
JN420339.1       ATGAGCCAGACCCAACCCCCGGCCCCAGTTGGGCCGGGCGACCCAGATGTTTACTTAAAA         60
MN159383.1       ATGAGCCAGACCCAACCCCCGGCCCCCGTTGGGCCGGGCGACCCAGATGTTTACTTAAAA         60
JN420337.1       ATGAGCCAGACCCAACCCCCGGCCCCCGTTGGGCCGGGCGACCCAGATGTTTACTTAAAA         60
JN420338.1       ATGAGCCAGACCCAACCCCCGGCCCCCGTTGGGCCGGGCGACCCAGATGTTTACTTAAAA         60
MH160388.1       ATGAGCCAGACCCAACCCCCGGCCCCAGTTGGGCCGGGCGACCCAGATGTTTACTTAAAA         60
                 ************************** ****** **************************

NC_001806.2      GGCGTGCCGTCCGCCGGCATGCACCCCAGAGGTGTTCACGCACCTCGAGGACACCCGCGC         120
X02138.1         GGCGTGCCGTCCGCCGGCATGCACCCCAGAGGTGTTCACGCACCTCGAGGACACCCGCGC         120
MH319852.1       GGCGTGCCGTCCGCCGGCATGCACCCCAGAGGTGTTCACGCACCTCGAGGACACCCGCGC         120
OP297873.1       GGCGTGCCGTCCGCCGGCATGCACCCCAGAGGTGTTCACGCACCTCGAGGACACCCGCGC         120
KR011283.1       GGCGTGCCGTCCGCCGGCATGCACCCCAGAGGTGTTCACGCACCTCGAGGACACCCGCGC         120
X14112.1         GGCGTGCCGTCCGCCGGCATGCACCCCAGAGGTGTTCACGCACCTCGAGGACACCCGCGC         120
KR011281.1       GGCGTGCCGTCCGCCGGCATGCACCCCAGAGGTGTTCACGCACCTCGAGGACACCCGCGC         120
MG646679.1       GGCGTGCCGTCCGCCGGCATGCACCCCAGAGGTGTTCACGCACCTCGAGGACACCCGCGC         120
OP297879.1       GGCGTGCCGTCCGCCGGCATGCACCCCAGAGGTGTTCACGCACCTCGAGGACACCCGCGC         120
MH160384.1       GGCGTGCCGTCCGCCGGCATGCACCCCAGAGGTGTTCACGCACCTCGAGGACACCCGCGC         120
JN420342.2       GGCGTGCCGTCCGCCGGCATGCACCCCAGAGGTGTTCACGCACCTCGAGGACACCCGCGC         120
MG999890.1       GGCGTGCCGTCCGCCGGCATGCACCCCAGAGGTGTTCACGCACCTCGAGGACACCCGCGC         120
MG999850.1       GGCGTGCCGTCCGCCGGCATGCACCCCAGAGGTGTTCACGCACCTCGAGGACACCCGCGC         120
MH999848.1       GGCGTGCCGTCCGCCGGCATGCACCCCAGAGGTGTTCACGCACCTCGAGGACACCCGCGC         120
MH160390.1       GGCGTGCCGTCCGCCGGCATGCACCCCAGAGGTGTTCACGCACCTCGAGGACACCCGCGC         120
GU734772.1       GGCGTGCCGTCCGCCGGCATGCACCCCAGAGGTGTTCACGCACCTCGAGGACACCCGCGC         120
MG999839.1       GGCGTGCCGTCCGCCGGCATGCACCCCAGAGGTGTTCACGCACCTCGAGGACACCCGCGC         120
JN420341.1       GGCGTGCCGTCCGCCGGCATGCACCCCAGAGGTGTTCACGCACCTCGAGGACACCCGCGC         120
JN400093.1       GGCGTGCCGTCCGCCGGCATGCACCCCAGAGGTGTTCACGCACCTCGAGGACACCCGCGC         120
MG999872.1       GGCGTGCCGTCCGCCGGCATGCACCCCAGAGGTGTTCACGCACCTCGAGGACACCCGCGC         120
JN420340.1       GGCGTGCCGTCCGCCGGCATGCACCCCAGAGGTGTTCACGCACCTCGAGGACACCCGCGC         120
JN420339.1       GGCGTGCCGTCCGCCGGCATGCACCCCAGAGGTGTTCACGCACCTCGAGGACACCCGCGC         120
MN159383.1       GGCATGCCGTCCGCCGGCATGCACCCCAGAGGTGTTCACGCACCTCGAGGACACCCGCGC         120
JN420337.1       GGCATGCCGTCCGCCGGCATGCACCCCAGAGGTGTTCACGCACCTCGAGGACACCCGCGC         120
JN420338.1       GGCATGCCGTCCGCCGGCATGCACCCCAGAGGTGTTCACGCACCTCGAGGACACCCGCGC         120
MH160388.1       GGCGTGCCGTCCGCCGGCATGCACCCCAGAGGTGTTCACGCACCTCGAGGACACCCGCGC         120
                 *** ********************************************************
```

```
NC_001806.2      ATGATCTCCGGACCCCCGCAACGGGGTGATAATGATCAAGCGGCGGGGCAATGTGGAGAT      180
X02138.1         ATGATCTCCGGACCCCCGCAACGGGGTGATAATGATCAAGCGGCGGGGCAATGTGGAGAT      180
MH319852.1       ATGATCTCCGGACCCCCGCAACGGGGTGATAATGATCAAGCGGCGGGGCAATGTGGAGAT      180
OP297873.1       ATGATCTCCGGACCCCCGCAACGGGGTGATAATGATCAAGCGGCGGGGCAATGTGGAGAT      180
KR011283.1       ATGATCTCCGGACCCCCGCAACGGGGTGATAATGATCAAGCGGCGGGGCAATGTGGAGAT      180
X14112.1         ATGATCTCCGGACCCCCGCAACGGGGTGATAATGATCAAGCGGCGGGGCAATGTGGAGAT      180
KR011281.1       ATGATCTCCGGACCCCCGCAACGGGGTGATAATGATCAAGCGGCGGGGCAATGTGGAGAT      180
MG646679.1       ATGATCTCCGGACCCCCGCAACGGGGTGATAATGATCAAGCGGCGGGGCAATGTGGAGAT      180
OP297879.1       ATGATCTCCGGACCCCCGCAACGGGGTGATAATGATCAAGCGGCGGGGCAATGTGGAGAT      180
MH160384.1       ATGATCTCCGGACCCCCGCAACGGGGTGATAATGATCAAGCGGCGGGGCAATGTGGAGAT      180
JN420342.2       ATGATCTCCGGACCCCCGCAACGGGGTGATAATGATCAAGCGGCGGGGCAATGTGGAGAT      180
MG999890.1       ATGATCTCCGGACCCCCGCAACGGGGTGATAATGATCAAGCGGCGGGGCAATGTGGAGAT      180
MG999850.1       ATGATCTCCGGACCCCCGCAACGGGGTGATAATGATCAAGCGGCGGGGCAATGTGGAGAT      180
MH999848.1       ATGATCTCCGGACCCCCGCAACGGGGTGATAATGATCAAGCGGCGGGGCAATGTGGAGAT      180
MH160390.1       ATGATCTCCGGACCCCCGCAACGGGGTGATAATGATCAAGCGGCGGGGCAATGTGGAGAT      180
GU734772.1       ATGATCTCCGGACCCCCGCAACGGGGTGATAATGATCAAGCGGCGGGGCAATGTGGAGAT      180
MG999839.1       ATGATCTCCGGACCCCCGCAACGGGGTGATAATGATCAAGCGGCGGGGCAATGTGGAGAT      180
JN420341.1       ATGATCTCCGGACCCCCGCAACGGGGTGATAATGATCAAGCGGCGGGGCAATGTGGAGAT      180
JN400093.1       ATGATCTCCGGACCCCCGCAACGGGGTGATAATGATCAAGCGGCGGGGCAATGTGGAGAT      180
MG999872.1       ATGATCTCCGGACCCCCGCAACGGGGTGATAATGATCAAGCGGCGGGGCAATGTGGAGAT      180
JN420340.1       ATGATCTCCGGACCCCCGCAACGGGGTGATAATGATCAAGCGGCGGGGCAATGTGGAGAT      180
JN420339.1       ATGATCTCCGGACCCCCGCAACGGGGTGATAATGATCAAGCGGCGGGGCAATGTGGAGAT      180
MN159383.1       ATGATCTCCGGACCCCCACAACGGGGTGATAATGATCAAGCGGCGGGGCAATGTGGAGAT      180
JN420337.1       ATGATCTCCGGACCCCCACAACGGGGTGATAATGATCAAGCGGCGGGGCAATGTGGAGAT      180
JN420338.1       ATGATCTCCGGACCCCCACAACGGGGTGATAATGATCAAGCGGCGGGGCAATGTGGAGAT      180
MH160388.1       ATGATCTCCGGACCCCCGCAACGGGGTGATAATGATCAAGCGGCGGGGCAATGTGGAGAT      180
                 ***************** ******************************************

NC_001806.2      TCGGGTCTACTACGAGTCGGTGCGGACACTACGATCTCGAAGCCATCTGAAGCCGTCCGA      240
X02138.1         TCGGGTCTACTACGAGTCGGTGCGGACACTACGATCTCGAAGCCATCTGAAGCCGTCCGA      240
MH319852.1       TCGGGTCTACTACGAGTCGGTGCGGACACTACGATCTCGAAGCCATCTGAAGCCGTCCGA      240
OP297873.1       TCGGGTCTACTACGAGTCGGTGCGGACACTACGATCTCGAAGCCATCTGAAGCCGTCCGA      240
KR011283.1       TCGGGTCTACTACGAGTCGGTGCGGACACTACGATCTCGAAGCCATCTGAAGCCGTCCGA      240
X14112.1         TCGGGTCTACTACGAGTCGGTGCGGACACTACGATCTCGAAGCCATCTGAAGCCGTCCGA      240
KR011281.1       TCGGGTCTACTACGAGTCGGTGCGGACACTACGATCTCGAAGCCATCTGAAGCCGTCCGA      240
MG646679.1       TCGGGTCTACTACGAGTCGGTGCGGACACTACGATCTCGAAGCCATCTGAAGCCGTCCGA      240
OP297879.1       TCGGGTCTACTACGAGTCGGTGCGGACACTACGATCTCGAAGCCATCTGAAGCCGTCCGA      240
MH160384.1       TCGGGTCTACTACGAGTCGGTGCGGACACTACGATCTCGAAGCCATCTGAAGCCGTCCGA      240
JN420342.2       TCGGGTCTACTACGAGTCGGTGCGGACACTACGATCTCGAAGCCATCTGAAGCCGTCCGA      240
MG999890.1       TCGGGTCTACTACGAGTCGGTGCGGACACTACGATCTCGAAGCCATCTGAAGCCGTCCGA      240
MG999850.1       TCGGGTCTACTACGAGTCGGTGCGGACACTACGATCTCGCAGCCATCTGAAGCCGTCCGA      240
MH999848.1       TCGGGTCTACTACGAGTCGGTGCGGACACTACGATCTCGAAGCCATCTGAAGCCGTCCGA      240
MH160390.1       TCGGGTCTACTACGAGTCGGTGCGGACACTACGATCTCGAAGCCATCTGAAGCCGTCCGA      240
GU734772.1       TCGGGTCTACTACGAGTCGGTGCGGACACTACGATCTCGAAGCCATCTGAAGCCGTCCGA      240
MG999839.1       TCGGGTCTACTACGAGTCGGTGCGGACACTACGATCTCGAAGCCATCTGAAGCCGTCCGA      240
JN420341.1       TCGGGTCTACTACGAGTCGGTGCGGACACTACGATCTCGAAGCCATCTGAAGCCGTCCGA      240
JN400093.1       TCGGGTCTACTACGAGTCGGTGCGGACACTACGATCTCGAAGCCATCTGAAGCCGTCCGA      240
MG999872.1       TCGGGTCTACTACGAGTCGGTGCGGACACTACGATCTCGAAGCCATCTGAAGCCGTCCGA      240
JN420340.1       TCGGGTCTACTACGAGTCGGTGCGGACACTACGATCTCGAAGCCATCTGAAGCCGTCCGA      240
JN420339.1       TCGGGTCTACTACGAGTCGGTGCGGACACTACGATCTCGAAGCCATCTGAAGCCGTCCGA      240
MN159383.1       TCGGGTCTACTACGAGTCGGTGCGGACACTACGATCTCGAAGCCATCTGAAGCCGTCCGA      240
JN420337.1       TCGGGTCTACTACGAGTCGGTGCGGACACTACGATCTCGAAGCCATCTGAAGCCGTCCGA      240
JN420338.1       TCGGGTCTACTACGAGTCGGTGCGGACACTACGATCTCGAAGCCATCTGAAGCCGTCCGA      240
MH160388.1       TCGGGTCTACTACGAGTCGGTGCGGACACTACGATCTCGAAGCCATCTGAAGCCGTCCGA      240
                 *************************************** ********************
```

```
NC_001806.2      CCGCCAACAATCCCCAGGACACCGCGTGTTCCCCGGGAGCCCCGGGTTCCGCGACCACCC      300
X02138.1         CCGCCAACAATCCCCAGGACACCGCGTGTTCCCCGGGAGCCCCGGGTTCCGCGACCACCC      300
MH319852.1       CCGCCAACAATCCCCAGGACACCGCGTGTTCCCCGGGAGCCCCGGGTTCCGCGACCACCC      300
OP297873.1       CCGCCAACAATCCCCAGGACACCGCGTGTTCCCCGGGAGCCCCGGGTTCCGCGACCACCC      300
KR011283.1       CCGCCAACAATCCCCAGGACACCGCGTGTTCCCCGGGAGCCCCGGGTTCCGCGACCACCC      300
X14112.1         CCGCCAACAATCCCCAGGACACCGCGTGTTCCCCGGGAGCCCCGGGTTCCGCGACCACCC      300
KR011281.1       CCGCCAACAATCCCCAGGACACCGCGTGTTCCCCGGGAGCCCCGGGTTCCGCGACCACCC      300
MG646679.1       CCGCCAACAATCCCCAGGACACCGCGTGTTCCCCGGGAGCCCCGGGTTCCGCGACCACCC      300
OP297879.1       CCGCCAACAATCCCCAGGACACCGCGTGTTCCCCGGGAGCCCCGGGTTCCGCGACCACCC      300
MH160384.1       CCGCCAACAATCCCCAGGACACCGCGTGTTCCCCGGGAGCCCCGGGTTCCGCGACCACCC      300
JN420342.2       CCGCCAACAATCCCCAGGACACCGCGTGTTCCCCGGGAGCCCCGGGTTCCGCGACCACCC      300
MG999890.1       CTGCCAACAATCCCCAGGACACCGCGTGTTCCCCGGGAGCCCCGGGTTCCGCGCCCACCC      300
MG999850.1       CCGCCAACAATCCCCAGGACACCGCGTGTTCCCCGGGAGCCCCGGGTTCCGCGACCACCC      300
MH999848.1       CCGCCAACAATCCCCAGGACACCGCGTGTTCCCCGGGAGCCCCGGGTTCCGCGACCACCC      300
MH160390.1       CCGCCAACAATCCCCAGGACACCGCGTGTTCCCCGGGAGTCCCGGGTTCCGCGACCACCC      300
GU734772.1       CCGCCAACAATCCCCAGGACACCGCGTGTTCCCCGGGAGCCCCGGGTTCCGCGACCACCC      300
MG999839.1       CTGCCAACAATCCCCAGGACACCGCGTGTTCCCCGGGAGCCCCGGGTTCCGCGCCCACCC      300
JN420341.1       CCGCCAACATTCCCCAGGACACCGCGTGTTCCCCGGGAGCCCCGGGTTCCGCGACCACCC      300
JN400093.1       CCGCCAACAATCCCCAGGACACCGCGTGTTCCCCGGGAGCCCCGGGTTCCGCGACCACCC      300
MG999872.1       CCACCAACAATCCCCAGGACACCGCGTGTTCCCCGGGAGCCCCGGGTTCCGCGACCACCC      300
JN420340.1       CCGCCAACAATCCCCAGGACACCGCGTGTTCCCCGGGAGCCCCGGGTTCCGCGACCACCC      300
JN420339.1       CCGCCAACAATCCCCAGGACACCGCGTGTTCCCCGGGAGCCCCGGGTTCCGCGACCACCC      300
MN159383.1       CCACCAACAATCCCCAGGACACCGCGTGTTCCCCGGGAGCCCCGGGTTCCGCGACCACCC      300
JN420337.1       CCACCAACAATCCCCAGGACACCGCGTGTTCCCCGGGAGCCCCGGGTTCCGCGACCACCC      300
JN420338.1       CCGCCAACAATCCCCAGGACACCGCGTGTTCCCCGGGAGCCCCGGGTTCCGCGACCACCC      300
MH160388.1       CCGCCAACAATCCCCAGGACACCGCGTGTTCCCCGGGAGCCCCGGGTTCCGCGACCACCC      300
                 *  ****** ***************************** ************* ******

NC_001806.2      CGAGAACCTAGGGAACCCAGAGTACCGCGAGCTCCCAGAGACCCCAGGGTACCGCGTGAC      360
X02138.1         CGAGAACCTAGGGAACCCAGAGTACCGCGAGCTCCCAGAGACCCCAGGGTACCGCGTGAC      360
MH319852.1       CGAGAACCTAGGGAACCCAGAGTACCGCGAGCTCCCAGAGACCCCAGGGTACCGCGTGAC      360
OP297873.1       CGAGAACCTAGGGAACCCAGAGTACCGCGAGCTCCCAGAGACCCCAGGGTACCGCGTGAC      360
KR011283.1       CGAGAACCTAGGGAACCCAGAGTACCGCGAGCTCCCAGAGACCCCAGGGTACCGCGTGAC      360
X14112.1         CGAGAACCTAGGGAACCCAGAGTACCGCGAGCTCCCAGAGACCCCAGGGTACCGCGTGAC      360
KR011281.1       CGAGAACCTAGGGAACCCAGAGTACCGCGAGCTCCCAGAGACCCCAGGGTACCGCGTGAC      360
MG646679.1       CGAGAACCTAGGGAACCCAGAGTACCGCGAGCTCCCAGAGACCCCAGGGTACCGCGTGAC      360
OP297879.1       CGAGAACCTAGGGAACCCAGAGTACCGCGAGCTCCCAGAGACCCCAGGGTACCGCGTGAC      360
MH160384.1       CGAGAACCTAGGGAACCCAGAGTACCGCGAGCTCCCAGAGACCCCAGGGTACCGCGTGAC      360
JN420342.2       CGAGAACCTAGGGAACCCAGAGTACCGCGAGCTCCCAGAGACCCCAGGGTACCGCGTGAC      360
MG999890.1       CGAGAACCTAGGGAACCCAGAGTACCGCGAGCTCCCAGAGACCCCAGGGTACCGCGTGAC      360
MG999850.1       CGAGAACCTAGGGAACCCAGAGTACCGCGAGCTCCCAGAGACCCCAGGGTACCGCGTGAC      360
MH999848.1       CGAGAACCTAGGGAACCCAGAGTACCGCGAGCTCCCAGAGACCCCAGGGTACCGCGTGAC      360
MH160390.1       CGAGAACCTAGGGAACCCAGAGTACCGCGAGCTCCCAGAGACCCCAGGGTACCGCGTGAC      360
GU734772.1       CGAGAACCTAGGGAACCCAGAGTACCGCGAGCTCCCAGAGACCCCAGGGTACCGCGTGAC      360
MG999839.1       CGAGAACCTAGGGAACCCAGAGTACCGCGAGCTCCCAGAGACCCCAGGGTACCGCGTGAC      360
JN420341.1       CGAGAACCTAGGGAACCCAGAGTACCGCGAGCTCCCAGAGACCCCAGGGTACCGCGTGAC      360
JN400093.1       CGAGAACCTAGGGAACCCAGAGTACCGCGAGCTCCCAGAGACCCCAGGGTACCGCGTGAC      360
MG999872.1       CGAGAACCTAGGGAACCCAGAGTACCGCGAGCTTCCAGAGACCCCAGGGTACCGCGTGAC      360
JN420340.1       CGAGAACCTAGGGAACCCAGAGTACCGCGAGCTCCCAGAGACCCCAGGGTACCGCGTGAC      360
JN420339.1       CGAGAACCTAGGGAACCCAGAGTACCGCGAGCTCCCAGAGACCCCAGGGTACCGCGTGAC      360
MN159383.1       CGAGAACCTAGGGAACCCAGAGTACCGCGAGCTTCCAGAGACCCCAGGGTACCGCGTGAC      360
JN420337.1       CGAGAACCTAGGGAACCCAGAGTACCGCGAGCTTCCAGAGACCCCAGGGTACCGCGTGAC      360
JN420338.1       CGAGAACCTAGGGAACCCAGAGTACCGCGAGCTCCCAGAGACCCCAGGGTACCGCGTGAC      360
MH160388.1       CGAGAACCTAGGGAACCCAGAGTACCGCGAGCTCCCAGAGACCCCAGGGTACCGCGTGAC      360
                 ********************************* **************************
```

```
NC_001806.2     CCCAGGGATCCACGACAACCCCGGTCTCCCAGGGAGCCCCGGTCTCCCCGGGAGCCCCGG     420
X02138.1        CCCAGGGATCCACGACAACCCCGGTCTCCCAGGGAGCCCCGGTCTCCCCGGGAGCCCCGG     420
MH319852.1      CCCAGGGATCCACGACAACCCCGGTCTCCCAGGGAGCCCCGGTCTCCCCGGGAGCCCCGG     420
OP297873.1      CCCAGGGATCCACGACAACCCCGGTCTCCCAGGGAGCCCCGGTCTCCCCGGGAGCCCCGG     420
KR011283.1      CCCAGGGATCCACGACAACCCCGGTCTCCCAGGGAGCCCCGGTCTCCCCGGGAGCCCCGG     420
X14112.1        CCCAGGGATCCACGACAACCCCGGTCTCCCAGGGAGCCCCGGTCTCCCCGGGAGCCCCGG     420
KR011281.1      CCCAGGGATCCACGACAACCCCGGTCTCCCAGGGAGCCCCGGTCTCCCCGGGAGCCCCGG     420
MG646679.1      CCCAGGGATCCACGACAACCCCGGTCTCCCAGGGAGCCCCGGTCTCCCAGGGAGCCCCGG     420
OP297879.1      CCCAGGGATCCACGACAACCCCGGTCTCCCAGGGAGCCCCGGTCTCCCAGGGAGCCCCGG     420
MH160384.1      CCCAGGGATCCACGACAACCCCGGTCTCCCAGGGAGCCCCGGTCTCCCAGGGAGCCCCGG     420
JN420342.2      CCCAGGGATCCACGACAACCCCGGTCTCCCAGGGAGCCCCGGTCTCCCAGGGAGCCCCGG     420
MG999890.1      CCCAGGGATCCACGACAACCCCGGTCTCCCAGGGAGCCCCGGTCTCCCCGGGAGCCCCGG     420
MG999850.1      CCCAGGGATCCACGACAACCCCGGTCTCCCAGGGAGCCCCGGTCTCCCAGGGAGCCCCGG     420
MH999848.1      CCCAGGGATCCACGACAACCCCGGTGTCCCAGGGAGCCCCGGTCTCCCAGGGAGCCCCGG     420
MH160390.1      CCCAGGGATCCACGACAACCCCGGTCTCCCAGGGAGCCCCGGTCTCCCAGGGAGCCCCGG     420
GU734772.1      CCCAGGGATCCACGACAACCCCGGTCTCCCAGGGAGCCCCGGTCTCCCCGGGAGCCCCGG     420
MG999839.1      CCCAGGGATCCACGACAACCCCGGTCTCCCAGGGAGCCCCGGTCTCCCCGGGAGCCCCGG     420
JN420341.1      CCCAGGGATCCACGACAACCCCGGTCTCCCAGGGAGCCCCGGTCTCCCCGGGAGCCCCGG     420
JN400093.1      CCCAGGGATCCACGACAACCCCGGTCTCCCAGGGAGCCCCGACATCCCCGGGAGCCCCGG     420
MG999872.1      CCCAGGGATCCACGACAGCCCCGGTCTCCCAGGGAGCCCCGGTCTCCCAGGGAGCCCCGG     420
JN420340.1      CCCAGGGATCCACGACAACCCCGGTCTCCCAGGGAGCCACGACAACCCCGGGAGCCCCGG     420
JN420339.1      CCCAGGGATCCACGACAACCCCGGTCCCCCAGGGAGCCACGACAACCCCGGGAGCCCCGG     420
MN159383.1      CCCAGGGATCCACGACAGCCCCGGTCTCCCAGGGAGCCCCGGCCTCCCCGGGAGCCACGG     420
JN420337.1      CCCAGGGATCCACGACAGCCCCGGTCTCCCAGGGAGCCCCGGTCTCCCAGGGAGCCCCGG     420
JN420338.1      CCCAGGGATCCACGACAACCCCGGGCCCCCAGGGAGCCACGACAACCCCGGGAGCCCCGG     420
MH160388.1      CCCAGGGCTCCCAGAGACCCCAGGGCTCCCAGAGACCCCAGGTGACCCCAGGAACCCCGG     420
                ******* ***  ** * *** **   ***** ** **  *    ***  *** ** ***

NC_001806.2     TCTCCCCGGGAGCCCCGGACCCCACGCACCCCCCGCGAACCACGTACGGCTCGCGGGTCT     480
X02138.1        TCTCCCCGGGAGCCCCGGACCCCACGCACCCCCCGCGAACCACGTACGGCTCGCGGGTCT     480
MH319852.1      TCTCCCCGGGAGCCCCGGACCCCACGCACCCCCCGCGAACCACGTACGGCTCGCGGGTCT     480
OP297873.1      TCTCCCCGGGAGCCCCGGACCCCACGCACCCCCCGCGAACCACGTACGGCTCGCGGGTCT     480
KR011283.1      TCTCCCCGGGAGCCCCGGACCCCACGCACCCCCCGCGAACCACGTACGGCTCGCGGGTCT     480
X14112.1        TCTCCCCGGGAGCCCCGGACCCCACGCACCCCCCGCGAACCACGTACGGCTCGCGGGTCT     480
KR011281.1      TCTCCCCGGGAGCCCCGGACCCCACGCACCCCCCGCGAACCACGTACGGCTCGCGGGTCT     480
MG646679.1      TCTCCCCGGGAGCCCCGGACCCCACGCACCCCCCGCGAACCACGTACGGCTCGCGGGTCT     480
OP297879.1      TCTCCCCGGGAGCCCCGGACCCCACGCACCCCCCGCGAACCACGTACGGCTCGCGGGTCT     480
MH160384.1      TCTCCCCGGGAGCCCCGGACCCCACGCACCCCCCGCGAACCACGTACGGCTCGCGGGTCT     480
JN420342.2      TCTCCCCGGGAGCCCCGGACCCCACGCACCCCCCGCGAACCACGTACGGCTCGCGGGTCT     480
MG999890.1      TCTCCCCGGGAGCCCCGGACCCCACGCACCCCCCGCGAACCACGTACGGCTCGCGGGTCT     480
MG999850.1      TCTCCCCGGGAGCCCCGGACCCCACGCACCCCCCGCGAACCACGTACGGCTCGCGGGTCT     480
MH999848.1      TCTCCCCGGGAGCCCCGGACCCCACGCACCCCCCGCGAACCACGTACGGCTCGCGGGTCT     480
MH160390.1      TCTCCCCGGGAGCCCCGGACCCCACGCACCCCCCGCGAACCACGTACGGCTCGCGGGTCT     480
GU734772.1      TCTCCCAGGGAGCCCCGGACCCCACGCACCCCCCGCGAACCACGTACGGCTCGCGGGTCT     480
MG999839.1      TCTCCCCGGGAGCCCCGGACCCCACGCACCCCCCGCGAACCACGTACGGCTCGCGGGTCT     480
JN420341.1      CATCCCCGGGAGCCCCGGACCCCACGCACCCCCCGCGAACCACGTACGGCTCGCGGGTCT     480
JN400093.1      CCTCCCCGGGAGCCCCGGACCCCACGCACCCCCCGCGAACCACGTACGGCTCGCGGGTCT     480
MG999872.1      CCTCCCCGGGAGCCACGGACCCCACGCACCCCCCGCGAACCACGTACGGCTCGCGGGTCT     480
JN420340.1      CCTCCCCGGGAGCCCCGGACCCCACGCACCCCCCGCGAACCACGTACGGCTCGCGGGTCT     480
JN420339.1      CCTCCCCGGGAGCCCCGGACCCCACGCACCCCCCGCGAACCACGTACGGCTCGCGGGTCT     480
MN159383.1      TCTCCCCGGGAGCCCCGGACCCCACGCACCCCCCGCGAACCACGTACGGCTCGCGGGTCT     480
JN420337.1      TCTCCCAGGGAGCCACGGACCCCACGCACCCCCCGCGAACCACGTACGGCTCGCGGGTCT     480
JN420338.1      CCTCCCCGGGAGCCCCGGACCCCACGCACCCCCCGCGAACCACGTACGGCTCGCGGGTCT     480
MH160388.1      TCTCCCAGGGAGCCCCGGACCCCACGCACCCCCCGCGAACCACGTACGGCTCGCGGGTCT     480
                  **** ******* *********************************************
```

NC_001806.2	GTATAG	486
X02138.1	GTATAG	486
MH319852.1	GTATAG	486
OP297873.1	GTATAG	486
KR011283.1	GTATAG	486
X14112.1	GTATAG	486
KR011281.1	GTATAG	486
MG646679.1	GTATAG	486
OP297879.1	GTATAG	486
MH160384.1	GTATAG	486
JN420342.2	GTATAG	486
MG999890.1	GTATAG	486
MG999850.1	GTATAG	486
MH999848.1	GTATAG	486
MH160390.1	GTATAG	486
GU734772.1	GTATAG	486
MG999839.1	GTATAG	486
JN420341.1	GTATAG	486
JN400093.1	GTATAG	486
MG999872.1	GTATAG	486
JN420340.1	GTATAG	486
JN420339.1	GTATAG	486
MN159383.1	GTATAG	486
JN420337.1	GTATAG	486
JN420338.1	GTATAG	486
MH160388.1	GTATAG	486
	******	
