# Supplementary material for: Discovery of a Novel Intron in US10/US11/US12 of HSV-1 Strain 17
Source: Viruses. 2023 Oct 25;15(11):2144. doi: 10.3390/v15112144 (PMC10675037; doi:10.3390/v15112144)
Supplement: Supplementary file 1 [file viruses-15-02144-s001.zip › Supplemental Data S2. 27-nt alignment.pdf]

Supplemental Data S2: Alignment of the HSV-1 US11 Gene without Intron

```
NC_001806.2      ATGAGCCAGACCCAACCCCCGGCCCCAGTTGGGCCGGGCGACCCAGATGTTTACTTAAAA   60
MN136524.1       ATGAGCCAGACCCAACCCCCGGCCCCAGTTGGGCCGGGCGACCCAGATGTTTACTTAAAA   60
KX791954.1       ATGAGCCAGACCCAACCCCCGGCCCCAGTTGGGCCGGGCGACCCAGATGTTTACTTAAAA   60
KX791808.1       ATGAGCCAGACCCAACCCCCGGCCCCAGTTGGGCCGGGCGACCCAGATGTTTACTTAAAA   60
KT425109.1       ATGAGCCAGACCCAACCCCCGGCCCCAGTTGGGCCGGGCGACCCAGATGTTTACTTAAAA   60
ON007141.1       ATGAGCCAGACCCAACCCCCGGCCCCAGTTGGGCCGGGCGACCCAGATGTTTACTTAAAA   60
ON007134.1       ATGAGCCAGACCCAACCCCCGGCCCCAGTTGGGCCGGGCGACCCAGATGTTTACTTAAAA   60
ON007145.1       ATGAGCCAGACCCAACCCCCGGCCCCAGTTGGGCCGGGCGACCCAGATGTTTACTTAAAA   60
OP297874.1       ATGAGCCAGACCCAACCCCCGGCCCCAGTTGGGCCGGGCGACCCAGATGTTTACTTAAAA   60
OP297882.1       ATGAGCCAGACCCAACCCCCGGCCCCAGTTGGGCCGGGCGACCCAGATGTTTACTTAAAA   60
HM585498.2       ATGAGCCAGACCCAACCCCCGGCCCCAGTTGGGCCGGGCGACCCAGATGTTTACTTAAAA   60
ON007160.1       ATGAGCCAGACCCAACCCCCGGCCCCAGTTGGGCCGGGCGACCCAGATGTTTACTTAAAA   60
HM585514.2       ATGAGCCAGACCCAACCCCCGGCCCCAGTTGGGCCGGGCGACCCAGATGTTTACTTAAAA   60
OP297867.1       ATGAGCCAGACCCAACCCCCGGCCCCAGTTGGGCCGGGCGACCCAGATGTTTACTTAAAA   60
LT594109.1       ATGAGCCAGACCCAACCCCCGGCCCCAGTTGGGCCGGGCGACCCAGATGTTTACTTAAAA   60
LT594112.1       ATGAGCCAGACCCAACCCCCGGCCCCAGTTGGGCCGGGCGACCCAGATGTTTACTTAAAA   60
HM585511.2       ATGAGCCAGACCCAACCCCCGGCCCCAGTTGGGCCGGGCGACCCAGATGTTTACTTAAAA   60
KF498959.1       ATGAGCCAGACCCAACCCCCGGCCCCAGTTGGGCCGGGCGACCCAGATGTTTACTTAAAA   60
MG999888.1       ATGAGCCAGACCCAACCCCCGGCCCCAGTTGGGCCGGGCGACCCAGATGTTTACTTAAAA   60
ON960058.1       ATGAGCCAGACCCAACCCCCGGCCCCAGTTGGGCCGGGCGACCCAGATGTTTACTTAAAA   60
MH160359.1       ATGAGCCAGACCCAACCCCCGGCCCCAGTTGGGCCGGGCGACCCAGATGTTTACTTAAAA   60
MG999885.1       ATGAGCCAGACCCAACCCCCGGCCCCAGTTGGGCCGGGCGACCCAGATGTTTACTTAAAA   60
MH999844.1       ATGAGCCAGACCCAACCCCCGGCCCCAGTTGGGCCGGGCGACCCAGATGTTTACTTAAAA   60
MH160361.1       ATGAGCCAGACCCAACCCCCGGCCCCAGTTGGGCCGGGCGACCCAGATGTTTACTTAAAA   60
MG999865.1       ATGAGCCAGACCCAACCCCCGGCCCCAGTTGGGCCGGGCGACCCAGATGTTTACTTAAAA   60
MG999876.1       ATGAGCCAGACCCAACCCCCGGCCCCAGTTGGGCCGGGCGACCCAGATGTTTACTTAAAA   60
GU734771.1       ATGAGCCAGACCCAACCCCCGGCCCCAGTTGGGCCGGGCGACCCAGATGTTTACTTAAAA   60
MG999869.1       ATGAGCCAGACCCAACCCCCGGCCCCAGTTGGGCCGGGCGACCCAGATGTTTACTTAAAA   60
MG999878.1       ATGAGCCAGACCCAACCCCCGGCCCCAGTTGGGCCGGGCGACCCAGATGTTTACTTAAAA   60
ON960060.1       ATGAGCCAGACCCAACCCCCGGCCCCAGTTGGGCCGGGCGACCCAGATGTTTACTTAAAA   60
MH999843.1       ATGAGCCAGACCCAACCCCCGGCCCCAGTTGGGCCGGGCGACCCAGATGTTTACTTAAAA   60
MH999850.1       ATGAGCCAGACCCAACCCCCGGCCCCAGTTGGGCCGGGCGACCCAGATGTTTACTTAAAA   60
MG999861.1       ATGAGCCAGACCCAACCCCCGGCCCCAGTTGGGCCGGGCGACCCAGATGTTTACTTAAAA   60
KX791871.1       ATGAGCCAGACCCAACCCCCGGCCCCAGTTGGGCCGGGCGACCCAGATGTTTACTTAAAA   60
OP297861.1       ATGAGCCAGACCCAACCCCCGGCCCCAGTTGGGCCGGGCGACCCAGATGTTTACTTAAAA   60
MG999864.1       ATGAGCCAGACCCAACCCCCGGCCCCAGTTGGGCCGGGCGACCCAGATGTTTACTTAAAA   60
OL638990.1       ATGAGCCAGACCCAACCCCCGGCCCCAGTTGGGCCGGGCGACCCAGATGTTTACTTAAAA   60
MH999838.1       ATGAGCCAGACCCAACCCCCGGCCCCAGTTGGGCCGGGCGACCCAGATGTTTACTTAAAA   60
KU310659.1       ATGAGCCAGACCCAACCCCCGGCCCCAGTTGGGCCGGGCGACCCAGATGTTTACTTAAAA   60
KX791923.1       ATGAGCCAGACCCAACCCCCGGCCCCCGTTGGGCCGGGCGACCCAGATGTTTACTTAAAA   60
MG999845.1       ATGAGCCAGACCCAACCCCCGGCCCCAGTTGGGCCGGGCGACCCAGATGTTTACTTAAAA   60
ON007132.1       ATGAGCCAGACCCAACCCCCGGCCCCAGTTGGGCCGGGCGACCCAGATGTTTACTTAAAA   60
HM585497.2       ATGAGCCAGACCCAACCCCCGGCCCCAGTTGGGCCGGGCGACCCAGATGTTTACTTAAAA   60
HM585500.2       ATGAGCCAGACCCAACCCCCGGCCCCAGTTGGGCCGGGCGACCCAGATGTTTACTTAAAA   60
HM585499.2       ATGAGCCAGACCCAACCCCCGGCCCCAGTTGGGCCGGGCGACCCAGATGTTTACTTAAAA   60
HM585504.2       ATGAGCCAGACCCAACCCCCGGCCCCAGTTGGGCCGGGCGACCCAGATGTTTACTTAAAA   60
HM585496.2       ATGAGCCAGACCCAACCCCCGGCCCCAGTTGGGCCGGGCGACCCAGATGTTTACTTAAAA   60
HM585506.2       ATGAGCCAGACCCAACCCCCGGCCCCAGTTGGGCCGGGCGACCCAGATGTTTACTTAAAA   60
ON007139.1       ATGAGCCAGACCCAACCCCCGGCCCCAGTTGGGCCGGGCGACCCAGATGTTTACTTAAAA   60
HM585509.2       ATGAGCCAGACCCAACCCCCGGCCCCAGTTGGGCCGGGCGACCCAGATGTTTACTTAAAA   60
OP297883.1       ATGAGCCAGACCCAACCCCCGGCCCCAGTTGGGCCGGGCGACCCAGATGTTTACTTAAAA   60
MN136523.1       ATGAGCCAGACCCAACCCCCGGCCCCAGTTGGGCCGGGCGACCCAGATGTTTACTTAAAA   60
MH999841.1       ATGAGCCAGACCCAACCCCCGGCCCCAGTTGGGCCGGGCGACCCAGATGTTTACTTAAAA   60
MH813983.1       ATGAGCCAGACCCAACCCCCGGCCCCCGTTGGGCCGGGCGACCCAGATGTTTACTTAAAA   60
MH813990.1       ATGAGCCAGACCCAACCCCCGGCCCCCGTTGGGCCGGGCGACCCAGATGTTTACTTAAAA   60
MH813994.1       ATGAGCCAGACCCAACCCCCGGCCCCCGTTGGGCCGGGCGACCCAGATGTTTACTTAAAA   60
MH813989.1       ATGAGCCAGACCCAACCCCCGGCCCCCGTTGGGCCGGGCGACCCAGATGTTTACTTAAAA   60
MH813985.1       ATGAGCCAGACCCAACCCCCGGCCCCCGTTGGGCCGGGCGACCCAGATGTTTACTTAAAA   60
MH813987.1       ATGAGCCAGACCCAACCCCCGGCCCCCGTTGGGCCGGGCGACCCAGATGTTTACTTAAAA   60
MH813993.1       ATGAGCCAGACCCAACCCCCGGCCCCCGTTGGGCCGGGCGACCCAGATGTTTACTTAAAA   60
MH813992.1       ATGAGCCAGACCCAACCCCCGGCCCCCGTTGGGCCGGGCGACCCAGATGTTTACTTAAAA   60
MH813988.1       ATGAGCCAGACCCAACCCCCGGCCCCCGTTGGGCCGGGCGACCCAGATGTTTACTTAAAA   60
MH813984.1       ATGAGCCAGACCCAACCCCCGGCCCCCGTTGGGCCGGGCGACCCAGATGTTTACTTAAAA   60
MH813982.1       ATGAGCCAGACCCAACCCCCGGCCCCCGTTGGGCCGGGCGACCCAGATGTTTACTTAAAA   60
OP297864.1       ATGAGCCAGACCCAACCCCCGGCCCCAGTTGGGCCGGGCGACCCAGATGTTTACTTAAAA   60
MH813991.1       ATGAGCCAGACCCAACCCCCGGCCCCCGTTGGGCCGGGCGACCCAGATGTTTACTTAAAA   60
MH813986.1       ATGAGCCAGACCCAACCCCCGGCCCCCGTTGGGCCGGGCGACCCAGATGTTTACTTAAAA   60
HM585512.2       ATGAGCCAGACCCAACCCCCGGCCCCAGTTGGGCCGTGCGACCCAGATGTTTACTTAAAA   60
HM585515.2       ATGAGCCAGACCCAACCCCCGGCCCCAGTTGGGCCGTGCGACCCAGATGTTTACTTAAAA   60
HM585513.2       ATGAGCCAGACCCAACCCCCGGCCCCAGTTGGGCCGTGCGACCCAGATGTTTACTTAAAA   60
MH999839.1       ATGAGCCAGACCCAACCCCCGGCCCCAGTTGGGCCGTGCGACCCAGATGTTTACTTAAAA   60
MH999840.1       ATGAGCCAGACCCAACCCCCGGCCCCAGTTGGGCCGGGCGACCCAGATGTTTACTTAAAA   60
                 **************************.********* ***********************
```

```
NC_001806.2        GGCGTGCCGTCCGCCGGCATGCACCCCAGAGGTGTTCACGCACCTCGAGGACACCCGCGC    120
MN136524.1         GGCGTGCCGTCCGCCGGCATGCACCCCAGAGGTGTTCACGCACCTCGAGGACACCCGCGC    120
KX791954.1         GGCGTGCCGTCCGCCGGCATGCACCCCAGAGGTGTTCACGCACCTCGAGGACACCCGCGC    120
KX791808.1         GGCGTGCCGTCCGCCGGCATGCACCCCAGAGGTGTTCACGCACCTCGAGGACACCCGCGC    120
KT425109.1         GGCGTGCCGTCCGCCGGCATGCACCCCAGAGGTGTTCACGCACCTCGAGGACACCCGCGC    120
ON007141.1         GGCGTGCCGTCCGCCGGCATGCACCCCAGAGGTGTTCACGCACCTCGAGGACACCCGCGC    120
ON007134.1         GGCGTGCCGTCCGCCGGCATGCACCCCAGAGGTGTTCACGCACCTCGAGGACACCCGCGC    120
ON007145.1         GGCGTGCCGTCCGCCGGCATGCACCCCAGAGGTGTTCACGCACCTCGAGGACACCCGCGC    120
OP297874.1         GGCGTGCCGTCCGCCGGCATGCACCCCAGAGGTGTTCACGCACCTCGAGGACACCCGCGC    120
OP297882.1         GGCGTGCCGTCCGCCGGCATGCACCCCAGAGGTGTTCACGCACCTCGAGGACACCCGCGC    120
HM585498.2         GGCGTGCCGTCCGCCGGCATGCACCCCAGAGGTGTTCACGCACCTCGAGGACACCCGCGC    120
ON007160.1         GGCGTGCCGTCCGCCGGCATGCACCCCAGAGGTGTTCACGCACCTCGAGGACACCCGCGC    120
HM585514.2         GGCGTGCCGTCCGCCGGCATGCACCCCAGAGGTGTTCACGCACCTCGAGGACACCCGCGC    120
OP297867.1         GGCGTGCCGTCCGCCGGCATGCACCCCAGAGGTGTTCACGCACCTCGAGGACACCCGCGC    120
LT594109.1         GGCGTGCCGTCCGCCGGCATGCACCCCAGAGGTGTTCACGCACCTCGAGGACACCCGCGC    120
LT594112.1         GGCGTGCCGTCCGCCGGCATGCACCCCAGAGGTGTTCACGCACCTCGAGGACACCCGCGC    120
HM585511.2         GGCGTGCCGTCCGCCGGCATGCACCCCAGAGGTGTTCACGCACCTCGAGGACACCCGCGC    120
KF498959.1         GGCGTGCCGTCCGCCGGCATGCACCCCAGAGGTGTTCACGCACCTCGAGGACACCCGCGC    120
MG999888.1         GGCGTGCCGTCCGCCGGCATGCACCCCAGAGGTGTTCACGCACCTCGAGGACACCCGCGC    120
ON960058.1         GGCGTGCCGTCCGCCGGCATGCACCCCAGAGGTGTTCACGCACCTCGAGGACACCCGCGC    120
MH160359.1         GGCGTGCCGTCCGCCGGCATGCACCCCAGAGGTGTTCACGCACCTCGAGGACACCCGCGC    120
MG999885.1         GGCGTGCCGTCCGCCGGCATGCACCCCAGAGGTGTTCACGCACCTCGAGGACACCCGCGC    120
MH999844.1         GGCGTGCCGTCCGCCGGCATGCACCCCAGAGGTGTTCACGCACCTCGAGGACACCCGCGC    120
MH160361.1         GGCGTGCCGTCCGCCGGCATGCACCCCAGAGGTGTTCACGCACCTCGAGGACACCCGCGC    120
MG999865.1         GGCGTGCCGTCCGCCGGCATGCACCCCAGAGGTGTTCACGCACCTCGAGGACACCCGCGC    120
MG999876.1         GGCGTGCCGTCCGCCGGCATGCACCCCAGAGGTGTTCACGCACCTCGAGGACACCCGCGC    120
GU734771.1         GGCGTGCCGTCCGCCGGCATGCACCCCAGAGGTGTTCACGCACCTCGAGGACACCCGCGC    120
MG999869.1         GGCGTGCCGTCCGCCGGCATGCACCCCAGAGGTGTTCACGCACCTCGAGGACACCCGCGC    120
MG999878.1         GGCGTGCCGTCCGCCGGCATGCACCCCAGAGGTGTTCACGCACCTCGAGGACACCCGCGC    120
ON960060.1         GGCGTGCCGTCCGCCGGCATGCACCCCAGAGGTGTTCACGCACCTCGAGGACACCCGCGC    120
MH999843.1         GGCGTGCCGTCCGCCGGCATGCACCCCAGAGGTGTTCACGCACCTCGAGGACACCCGCGC    120
MH999850.1         GGCGTGCCGTCCGCCGGCATGCACCCCAGAGGTGTTCACGCACCTCGAGGACACCCGCGC    120
MG999861.1         GGCGTGCCGTCCGCCGGCATGCACCCCAGAGGTGTTCACGCACCTCGAGGACACCCGCGC    120
KX791871.1         GGCGTGCCGTCCGCCGGCATGCACCCCAGAGGTGTTCACGCACCTCGAGGACACCCGCGC    120
OP297861.1         GGCGTGCCGTCCGCCGGCATGCACCCCAGAGGTGTTCACGCACCTCAAGGACACCCGCGC    120
MG999864.1         GGCGTGCCGTCCGCCGGCATGCACCCCAGAGGTGTTCACGCACCTCGAGGACACCCGCGC    120
OL638990.1         GGCGTGCCGTCCGCCGGCATGCACCCCAGAGGTGTTCACGCACCTCGAGGACACCCGCGC    120
MH999838.1         GGCGTGCCGTCCGCCGGCATGCACCCCAGAGGTGTTCACGCACCTCGAGGACACCCGCGC    120
KU310659.1         GGCGTGCCGTCCGCCGGCATGCACCCCAGAGGTGTTCACGCACCTCGAGGACACCCGCGC    120
KX791923.1         GGCGTGCCGTCCGCCGGCATGCACCCCAGAGGTGTTTACGCACCTCGAGGACACCCGCGC    120
MG999845.1         GGCGTGCCGTCCGCCGGCATGCACCCCAGAGGTGTTCACGCACCTCCAGGACACCCGCGC    120
ON007132.1         GGCGTGCCGTCCGCCGGCATGCACCCCAGAGGTGTTCACGCACCTCGAGGACACCCGCAC    120
HM585497.2         GGCGTGCCGTCCGCCGGCATGCACCCCAGAGGTGTTCACGCACCTCCAGGACACCCGCGC    120
HM585500.2         GGCGTGCCGTCCGCCGGCATGCACCCCAGAGGTGTTCACGCACCTCCAGGACACCCGCGC    120
HM585499.2         GGCGTGCCGTCCGCCGGCATGCACCCCAGAGGTGTTCACGCACCTCCAGGACACCCGCGC    120
HM585504.2         GGCGTGCCGTCCGCCGGCATGCACCCCAGAGGTGTTCACGCACCTCCAGGACACCCGCGC    120
HM585496.2         GGCGTGCCGTCCGCCGGCATGCACCCCAGAGGTGTTCACGCACCTCCAGGACACCCGCGC    120
HM585506.2         GGCGTGCCGTCCGCCGGCATGCACCCCAGAGGTGTTCACGCACCTCCAGGACACCCGCGC    120
ON007139.1         GGCGTGCCGTCCGCCGGCATGCACCCCAGAGGTGTTCACGCACCTCGAGGACACCCGCAC    120
HM585509.2         GGCGTGCCGTCCGCCGGCATGCACCCCAGAGGTGTTCACGCACCTCGAGGACACCCGCGC    120
OP297883.1         GGCGTGCCGTCCGCCGGCATGCACCCCAGAGGTGTTCACGCACCTCGAGGACACCCGCGC    120
MN136523.1         GGCGTGCCGTCCGCCGGCATGCACCCCAGAGGTGTTCACGCACATCGAGGACACCCGCGC    120
MH999841.1         GGCGTGCCGTCCGCCGGCATGCACCCCAGAGGTGTTCACGCACCTCGAGGACACCCGCGC    120
MH813983.1         GGCGTGCCGTTCGCCGGCATGCACCCCAGAGGTGTTCACGCACCTCGAGGACACCCGCGC    120
MH813990.1         GGCGTGCCGTTCGCCGGCATGCACCCCAGAGGTGTTCACGCACCTCGAGGACACCCGCGC    120
MH813994.1         GGCGTGCCGTTCGCCGGCATGCACCCCAGAGGTGTTCACGCACCTCGAGGACACCCGCGC    120
MH813989.1         GGCGTGCCGTTCGCCGGCATGCACCCCAGAGGTGTTCACGCACCTCGAGGACACCCGCGC    120
MH813985.1         GGCGTGCCGTTCGCCGGCATGCACCCCAGAGGTGTTCACGCACCTCGAGGACACCCGCGC    120
MH813987.1         GGCGTGCCGTTCGCCGGCATGCACCCCAGAGGTGTTCACGCACCTCGAGGACACCCGCGC    120
MH813993.1         GGCGTGCCGTTCGCCGGCATGCACCCCAGAGGTGTTCACGCACCTCGAGGACACCCGCGC    120
MH813992.1         GGCGTGCCGTTCGCCGGCATGCACCCCAGAGGTGTTCACGCACCTCGAGGACACCCGCGC    120
MH813988.1         GGCGTGCCGTTCGCCGGCATGCACCCCAGAGGTGTTCACGCACCTCGAGGACACCCGCGC    120
MH813984.1         GGCGTGCCGTTCGCCGGCATGCACCCCAGAGGTGTTCACGCACCTCGAGGACACCCGCGC    120
MH813982.1         GGCGTGCCGTTCGCCGGCATGCACCCCAGAGGTGTTCACGCACCTCGAGGACACCCGCGC    120
OP297864.1         GGCGTGCCGTCCGCCGGCATGCACCCCAGAGGTGTTCACGCACCTCGAGGACACCCGCAC    120
MH813991.1         GGCGTGCCGTTCGCCGGCATGCACCCCAGAGGTGTTCACGCACCTCGAGGACACCCGCGC    120
MH813986.1         GGCGTGCCGTTCGCCGGCATGCACCCCAGAGGTGTTCACGCACCTCGAGGACACCCGCGC    120
HM585512.2         GGCGTGCCGTCCGCCGGCATGCACCCCAGAGGTGTTCACGCACCTCGAGGACACCCGCAC    120
HM585515.2         GGCGTGCCGTCCGCCGGCATGCACCCCAGAGGTGTTCACGCACCTCGAGGACACCCGCAC    120
HM585513.2         GGCGTGCCGTCCGCCGGCATGCACCCCAGAGGTGTTCACGCACCTCGAGGACACCCGCAC    120
MH999839.1         GGCGTGCCGTCCGCCGGCATGCACCCCAGAGGTGTTCACGCACCTCGAGGACACCCGCAC    120
MH999840.1         GGCGTGCCGTCCGCCGGCATGCACCCCAGAGGTGTTCACGCACCTCGAGGACACCCGCAC    120
                   ********** ************************* ******.** ***********.*
```

```
NC_001806.2        ATGATCTCCGGACCCCCGCAACGGGGTGATAATGATCAAGCGGCGGGGCAATGTGGAGAT   180
MN136524.1         ATGATCTCCGGACCCCCGCAACGGGGTGATAATGATCAAGCGGCAGGGCAATGTGGAGAT   180
KX791954.1         ATGATCTCCGGACCCCCGCAACGGGGTGATAATGATCAAGCGGCGGGGCAATGTGGAGAT   180
KX791808.1         ATGATCTCCGGACCCCCGCAACGGGGTGATAATGATCAAGCGGCGGGGCAATGTGGAGAT   180
KT425109.1         ATGATCTCCGGACCCCCGCAACGGGGTGATAATGATCAAGCGGCGGGGCAATGTGGAGAT   180
ON007141.1         ATGATCTCCGGACCCCCGCAACGGGGTGATAATGATCAAGCGGCGGGGCAATGTGGAGAT   180
ON007134.1         ATGATCTCCGGACCCCCGCAACGGGGTGATAATGATCAAGCGGCGGGGCAATGTGGAGAT   180
ON007145.1         ATGATCTCCGGACCCCCGCAACGGGGTGATAATGATCAAGCGGCGGGGCAATGTGGAGAT   180
OP297874.1         ATGATCTCCGGACCCCCGCAACGGGGTGATAATGATCAAGCGGCGGGGCAATGTGGAGAT   180
OP297882.1         ATGATCTCCGGACCCCCGCAACGGGGTGATAATGATCAAGCGGCGGGGCAATGTGGAGAT   180
HM585498.2         ATGATCTCCGGACCCCCGCAACGGGGTGATAATGATCAAGCGGCGGGGCAATGTGGAGAT   180
ON007160.1         ATGATCTCCGGACCCCCGCAACGGGGTGATAATGATCAAGCGGCGGGGCAATGTGGAGAT   180
HM585514.2         ATGATCTCCGGACCCCCGCAACGGGGTGATAATGATCAAGCGGCGGGGCAATGTGGAGAT   180
OP297867.1         ATGATCTCCGGACCCCCGCAACGGGGTGATAATGATCAAGCGGCGGGGCAATGTGGAGAT   180
LT594109.1         ATGATCTCCGGACCCCCGCAACGGGGTGATAATGATCAAGCGGCGGGGCAATGTGGAGAT   180
LT594112.1         ATGATCTCCGGACCCCCGCAACGGGGTGATAATGATCAAGCGGCGGGGCAATGTGGAGAT   180
HM585511.2         ATGATCTCCGGACCCCCGCAACGGGGTGATAATGATCAAGCGGCGGGGCAATGTGGAGAT   180
KF498959.1         ATGATCTCCGGACCCCCGCAACGGGGTGATAATGATCAAGCGGCGGGGCAATGTGGAGAT   180
MG999888.1         ATGATCTCCGGACCCCCGCAACGGGGTGATAATGATCAAGCGGCGGGGCAATGTGGAGAT   180
ON960058.1         ATGATCTCCGGACCCCCGCAACGGGGTGATAATGATCAAGCGGCGGGGCAATGTGGAGAT   180
MH160359.1         ATGATCTCCGGACCCCCGCAACGGGGTGATAATGATCAAGCGGCGGGGCAATGTGGAGAT   180
MG999885.1         ATGATCTCCGGACCCCCGCAACGGGGTGATAATGATCAAGCGGCGGGGCAATGTGGAGAT   180
MH999844.1         ATGATCTCCGGACCCCCGCAACGGGGTGATAATGATCAAGCGGCGGGGCAATGTGGAGAT   180
MH160361.1         ATGATCTCCGGACCCCCGCAACGGGGTGATAATGATCAAGCGGCGGGGCAATGTGGAGAT   180
MG999865.1         ATGATCTCCGGACCCCCGCAACGGGGTGATAATGATCAAGCGGCGGGGCAATGTGGAGAT   180
MG999876.1         ATGATCTCCGGACCCCCGCAACGGGGTGATAATGATCAAGCGGCGGGGCAATGTGGAGAT   180
GU734771.1         ATGATCTCCGGACCCCCGCAACGGGGTGATAATGATCAAGCGGCGGGGCAATGTGGAGAT   180
MG999869.1         ATGATCTCCGGACCCCCGCAACGGGGTGATAATGATCAAGCGGCGGGGCAATGTGGAGAT   180
MG999878.1         ATGATCTCCGGACCCCCGCAACGGGGTGATAATGATCAAGCGGCGGGGCAATGTGGAGAT   180
ON960060.1         ATGATCTCCGGACCCCCGCAACGGGGTGATAATGATCAAGCGGCGGGGCAATGTGGAGAT   180
MH999843.1         ATGATCTCCGGACCCCCGCAACGGGGTGATAATGATCAAGCGGCGGGGCAATGTGGAGAT   180
MH999850.1         ATGATCTCCGGACCCCCGCAACGGGGTGATAATGATCAAGCGGCGGGGCAATGTGGAGAT   180
MG999861.1         ATGATCTCCGGACCCCCGCAACGGGGTGATAATGATCAAGCGGCGGGGCAATGTGGAGAT   180
KX791871.1         ATGATCTCCGGACCCCCGCAACGGGGTGATAATGATCAAGCGGCGGGGCAATGTGGAGAT   180
OP297861.1         ATGATCTCCGGACCCCCGCAACGGGGTGATAATGATCAAGCGGCGGGGCAATGTGGAGAT   180
MG999864.1         ATGATCTCCGGACCCCCGCAACGGGGTGATAATGATCAAGCGGCGGGGCAATGTGGAGAT   180
OL638990.1         ATGATCTCCGGACCCCCGCAACGGGGTGATAATGATCAAGCGGCAGGGCAATGTGGAGAT   180
MH999838.1         ATGATCTCCGGACCCCCGCAACGGGGTGATAATGATCAAGCGGCGGGGCAATGTGGAGAT   180
KU310659.1         ATGATCTCCGGACACCCCGCAACGGGGTGATAATGATCAAGCGGCAGGGCAATGTGGAGAT  180
KX791923.1         ATGATCTCCGGACCCCCGCAACGGGGTGATAATGATCAAGCGGCGGGGCAATGTGGAGAT   180
MG999845.1         ATGATCTCCGGACCCCCGCAACGGGGTGATAATGATCAAGCGGCGGGGCAATGTGGAGAT   180
ON007132.1         CTGATCTCCGGACCCCCGCAACGGGGTGATAATGATCAAGCGGCGGGGCAATGTGGAGAT   180
HM585497.2         ATGATCTCCGGACCCCCGCAACGGGGTGATAATGATCAAGCGGCGGGGCAATGTGGAGAT   180
HM585500.2         ATGATCTCCGGACCCCCGCAACGGGGTGATAATGATCAAGCGGCGGGGCAATGTGGAGAT   180
HM585499.2         ATGATCTCCGGACCCCCGCAACGGGGTGATAATGATCAAGCGGCGGGGCAATGTGGAGAT   180
HM585504.2         ATGATCTCCGGACCCCCGCAACGGGGTGATAATGATCAAGCGGCGGGGCAATGTGGAGAT   180
HM585496.2         ATGATCTCCGGACCCCCGCAACGGGGTGATAATGATCAAGCGGCGGGGCAATGTGGAGAT   180
HM585506.2         ATGATCTCCGGACCCCCGCAACGGGGTGATAATGATCAAGCGGCGGGGCAATGTGGAGAT   180
ON007139.1         CTGATCTCCGGACCCCCGCAACGGGGTGATAATGATCAAGCGGCGGGGCAATGTGGAGAT   180
HM585509.2         ATGATCTCCGGACCCCCGCAACGGGGTGATAATGATCAAGCGGCGGGGCAATGTGGAGAT   180
OP297883.1         ATGATCTCCGGACCCCCGCAACGGGGTGATAATGATCAAGCGGCGGGGCAATGTGGAGAT   180
MN136523.1         ATGATCTCCGGACCCCCGCAACGGGGTGATAATGATCAAGCGGCGGGGCAATGTGGAGAT   180
MH999841.1         ATGATCTCCGGACCCCCGCAACGGGGTGATAATGATCAAGCGGCGGGGCAATGTGGAGAT   180
MH813983.1         ATGATCTCCGGACCCCCGCAACGGGGTGATAATGATCAAGCGGCGGGGCAATGTGGAGAT   180
MH813990.1         ATGATCTCCGGACCCCCGCAACGGGGTGATAATGATCAAGCGGCGGGGCAATGTGGAGAT   180
MH813994.1         ATGATCTCCGGACCCCCGCAACGGGGTGATAATGATCAAGCGGCGGGGCAATGTGGAGAT   180
MH813989.1         ATGATCTCCGGACCCCCGCAACGGGGTGATAATGATCAAGCGGCGGGGCAATGTGGAGAT   180
MH813985.1         ATGATCTCCGGACCCCCGCAACGGGGTGATAATGATCAAGCGGCGGGGCAATGTGGAGAT   180
MH813987.1         ATGATCTCCGGACCCCCGCAACGGGGTGATAATGATCAAGCGGCGGGGCAATGTGGAGAT   180
MH813993.1         ATGATCTCCGGACCCCCGCAACGGGGTGATAATGATCAAGCGGCGGGGCAATGTGGAGAT   180
MH813992.1         ATGATCTCCGGACCCCCGCAACGGGGTGATAATGATCAAGCGGCGGGGCAATGTGGAGAT   180
MH813988.1         ATGATCTCCGGACCCCCGCAACGGGGTGATAATGATCAAGCGGCGGGGCAATGTGGAGAT   180
MH813984.1         ATGATCTCCGGACCCCCGCAACGGGGTGATAATGATCAAGCGGCGGGGCAATGTGGAGAT   180
MH813982.1         ATGATCTCCGGACCCCCGCAACGGGGTGATAATGATCAAGCGGCGGGGCAATGTGGAGAT   180
OP297864.1         CTGATCTCCGGACCCCCGCAACGGGGTGATAATGATCAAGCGGCGGGGCAATGTGGAGAT   180
MH813991.1         ATGATCTCCGGACCCCCGCAACGGGGTGATAATGATCAAGCGGCGGGGCAATGTGGAGAT   180
MH813986.1         ATGATCTCCGGACCCCCGCAACGGGGTGATAATGATCAAGCGGCGGGGCAATGTGGAGAT   180
HM585512.2         ATGATCTCCGGATCCCCGCAACGGGGTGATAATGATCAAGCGGCGGGGCAATGTGGAGAT   180
HM585515.2         ATGATCTCCGGATCCCCGCAACGGGGTGATAATGATCAAGCGGCGGGGCAATGTGGAGAT   180
HM585513.2         ATGATCTCCGGATCCCCGCAACGGGGTGATAATGATCAAGCGGCGGGGCAATGTGGAGAT   180
MH999839.1         ATGATCTCCGGATCCCCGCAACGGGGTGATAATGATCAAGCGGCGGGGCAATGTGGAGAT   180
MH999840.1         ATGATCTCCGGACCCCCGCAACGGGGTGATAATGATCAAGCGGCGGGGCAATGTGGAGAT   180
                   .*********** .*****************************.****************
```

```
NC_001806.2      TCGGGTCTACTACGAGTCGGTGCGGACACTACGATCTCGAAGCCATCTGAAGCCGTCCGA   240
MN136524.1       TCGGGTCTACTACGAGTCGGTGCGGACACTACGATCTCGAAGCCATCTGAAGCCGTCCGA   240
KX791954.1       TCGGGTCTACTACGAGTCGGTGCGGACACTACGATCTCGAAGCCATCTGAAGCCGTCCGA   240
KX791808.1       TCGGGTCTACTACGAGTCGGTGCGGACACTACGATCTCGAAGCCATCTGAAGCCGTCCGA   240
KT425109.1       TCGGGTCTACTACGAGTCGGTGCGGACACTACGATCTCGAAGCCATCTGAAGCCGTCCGA   240
ON007141.1       TCGGGTCTACTACGAGTCGGTGCGGACACTACGATCTCGAAGCCATCTGAAGCCGTCCGA   240
ON007134.1       TCGGGTCTACTACGAGTCGGTGCGGACACTACGATCTCGAAGCCATCTGAAGCCGTCCGA   240
ON007145.1       TCGGGTCTACTACGAGTCGGTGCGGACACTACGATCTCGAAGCCATCTGAAGCCGTCCGA   240
OP297874.1       TCGGGTCTACTACGAGTCGGTGCGGACACTACGATCTCGAAGCCATCTGAAGCCGTCCGA   240
OP297882.1       TCGGGTCTACTACGAGTCGGTGCGGACACTACGATCTCGAAGCCATCTGAAGCCGTCCGA   240
HM585498.2       TCGGGTCTACTACGAGTCGGTGCGGACACTACGATCTCGAAGCCATCTGAAGCCGTCCGA   240
ON007160.1       TCGGGTCTACTACGAGTCGGTGCGGACACTACGATCTCGAAGCCATCTGAAGCCGTCCGA   240
HM585514.2       TCGGGTCTACTACGAGTCGGTGCGGACACTACGATCTCGAAGCCATCTGAAGCCGTCCGA   240
OP297867.1       TCGGGTCTACTACGAGTCGGTGCGGACACTACGATCTCGAAGCCATCTGAAGCCGTCCGA   240
LT594109.1       TCGGGTCTACTACGAGTCGGTGCGGACACTACGATCTCGAAGCCATCTGAAGCCGTCCGA   240
LT594112.1       TCGGGTCTACTACGAGTCGGTGCGGACACTACGATCTCGAAGCCATCTGAAGCCGTCCGA   240
HM585511.2       TCGGGTCTACTACGAGTCGGTGCGGACACTACGATCTCGAAGCCATCTGAAGCCGTCCGA   240
KF498959.1       TCGGGTCTACTACGAGTCGGTGCGGACACTACGATCTCGAAGCCATCTGAAGCCGTCCGA   240
MG999888.1       TCGGGTCTACTACGAGTCGGTGCGGACACTACGATCTCGAAGCCATCTGAAGCCGTCCGA   240
ON960058.1       TCGGGTCTACTACGAGTCGGTGCGGACACTACGATCTCGAAGCCATCTGAAGCCGTCCGA   240
MH160359.1       TCGGGTCTACTACGAGTCGGTGCGGACACTACGATCTCGAAGCCATCTGAAGCCGTCCGA   240
MG999885.1       TCGGGTCTACTACGAGTCGGTGCGGACACTACGATCTCGAAGCCATCTGAAGCCGTCCGA   240
MH999844.1       TCGGGTCTACTACGAGTCGGTGCGGACACTACGATCTCGAAGCCATCTGAAGCCGTCCGA   240
MH160361.1       TCGGGTCTACTACGAGTCGGTGCGGACACTACGATCTCGAAGCCATCTGAAGCCGTCCGA   240
MG999865.1       TCGGGTCTACTACGAGTCGGTGCGGACACTACGATCTCGAAGCCATCTGAAGCCGTCCGA   240
MG999876.1       TCGGGTCTACTACGAGTCGGTGCGGACACTACGATCTCGAAGCCATCTGAAGCCGTCCGA   240
GU734771.1       TCGGGTCTACTACGAGTCGGTGCGGACACTACGATCTCGAAGCCATCTGAAGCCGTCCGA   240
MG999869.1       TCGGGTCTACTACGAGTCGGTGCGGACACTACGATCTCGAAGCCATCTGAAGCCGTCCGA   240
MG999878.1       TCGGGTCTACTACGAGTCGGTGCGGACACTACGATCTCGAAGCCATCTGAAGCCGTCCGA   240
ON960060.1       TCGGGTCTACTACGAGTCGGTGCGGACACTACGATCTCGAAGCCATCTGAAGCCGTCCGA   240
MH999843.1       TCGGGTCTACTACGAGTCGGTGCGGACACTACGATCTCGAAGCCATCTGAAGCCGTCCGA   240
MH999850.1       TCGGGTCTACTACGAGTCGGTGCGGACACTACGATCTCGAAGCCATCTGAAGCCGTCCGA   240
MG999861.1       TCGGGTCTACTACGAGTCGGTGCGGACACTACGATCTCGAAGCCATCTGAAGCCGTCCGA   240
KX791871.1       TCGGGTCTACTACGAGTCGGTGCGGACACTACGATCTCGAAGCCATCTGAAGCCGTCCGA   240
OP297861.1       TCGGGTCTACTACGAGTCGGTGCGGACACTACGATCTCGAAGCCATCTGAAGCCGTCCGA   240
MG999864.1       TCGGGTCTACTACGAGTCGGTGCGGACACTACGATCTCGAAGCCATCTGAAGCCGTCCGA   240
OL638990.1       TCGGGTCTACTACGAGTCGGTGCGGACACTACGATCTCGAAGCCATCTGAAGCCGTCCGA   240
MH999838.1       TCGGGTCTACTACGAGTCGGTGCGGACACTACGATCTCGAAGCCATCTGAAGCCGTCCGA   240
KU310659.1       TCGGGTCTACTACGAGTCGGTGCGGACACTACGATCTCGAAGCCATCTGAAGCCGTCCGA   240
KX791923.1       TCGGGTCTACTACGAGTCGGTGCGGACACTACGATCTCGAAGCCATCTGAAGCCGTCCGA   240
MG999845.1       TCGGGTCTACTACGAGTCGGTGCGGACACTACGATCTCGAAGCCATCTGAAGCCGTCCGA   240
ON007132.1       TCGGGTCTACTACGAGTCGGTGCGGACACTACGATCTCGAAGCCATCTGAAGCCGTCCGA   240
HM585497.2       TCGGGTCTACTACGAGTCGGTGCGGACACTACGATCTCGAAGCCATCTGAAGCCGTCCGA   240
HM585500.2       TCGGGTCTACTACGAGTCGGTGCGGACACTACGATCTCGAAGCCATCTGAAGCCGTCCGA   240
HM585499.2       TCGGGTCTACTACGAGTCGGTGCGGACACTACGATCTCGAAGCCATCTGAAGCCGTCCGA   240
HM585504.2       TCGGGTCTACTACGAGTCGGTGCGGACACTACGATCTCGAAGCCATCTGAAGCCGTCCGA   240
HM585496.2       TCGGGTCTACTACGAGTCGGTGCGGACACTACGATCTCGAAGCCATCTGAAGCCGTCCGA   240
HM585506.2       TCGGGTCTACTACGAGTCGGTGCGGACACTACGATCTCGAAGCCATCTGAAGCCGTCCGA   240
ON007139.1       TCGGGTCTACTACGAGTCGGTGCGGACACTACGATCTCGAAGCCATCTGAAGCCGTCCGA   240
HM585509.2       TCGGGTCTACTACGAATCGGTGCGGACACTACGATCTCGAAGCCATCTGAAGCCGTCCGA   240
OP297883.1       TCGGGTCTACTACGAGTCGGTGCGGACACTACGATCTCGAAGCCATCTGAAGCCGTCCGA   240
MN136523.1       TCGGGTCTACTACGAGTCGGTGCGGACACTACGATCTCGAAGCCATCTGAAGCCGTCCGA   240
MH999841.1       TCGGGTCTACTACGAGTCGGTGCGGACACTACGATCTCGAAGCCATCTGAAGCCGTCCGA   240
MH813983.1       TCGGGTCTACTACGAGTCGGTGCGGACACTACGATCTCGAAGCCATCTGAAGCCGTCCGA   240
MH813990.1       TCGGGTCTACTACGAGTCGGTGCGGACACTACGATCTCGAAGCCATCTGAAGCCGTCCGA   240
MH813994.1       TCGGGTCTACTACGAGTCGGTGCGGACACTACGATCTCGAAGCCATCTGAAGCCGTCCGA   240
MH813989.1       TCGGGTCTACTACGAGTCGGTGCGGACACTACGATCTCGAAGCCATCTGAAGCCGTCCGA   240
MH813985.1       TCGGGTCTACTACGAGTCGGTGCGGACACTACGATCTCGAAGCCATCTGAAGCCGTCCGA   240
MH813987.1       TCGGGTCTACTACGAGTCGGTGCGGACACTACGATCTCGAAGCCATCTGAAGCCGTCCGA   240
MH813993.1       TCGGGTCTACTACGAGTCGGTGCGGACACTACGATCTCGAAGCCATCTGAAGCCGTCCGA   240
MH813992.1       TCGGGTCTACTACGAGTCGGTGCGGACACTACGATCTCGAAGCCATCTGAAGCCGTCCGA   240
MH813988.1       TCGGGTCTACTACGAGTCGGTGCGGACACTACGATCTCGAAGCCATCTGAAGCCGTCCGA   240
MH813984.1       TCGGGTCTACTACGAGTCGGTGCGGACACTACGATCTCGAAGCCATCTGAAGCCGTCCGA   240
MH813982.1       TCGGGTCTACTACGAGTCGGTGCGGACACTACGATCTCGAAGCCATCTGAAGCCGTCCGA   240
OP297864.1       TCGGGTCTACTACGAGTCGGTGCGGCCACTACGATCTCGAAGCCATCTGAAGCCGTCCGA   240
MH813991.1       TCGGGTCTACTACGAGTCGGTGCGGACACTACGATCTCGAAGCCATCTGAAGCCGTCCGA   240
MH813986.1       TCGGGTCTACTACGAGTCGGTGCGGACACTACGATCTCGAAGCCATCTGAAGCCGTCCGA   240
HM585512.2       TCGGGTCTACTACGAGTCGGTGCGGACACTACGATCTCGAAGCCATCTGAAGCCGTCCGA   240
HM585515.2       TCGGGTCTACTACGAGTCGGTGCGGACACTACGATCTCGAAGCCATCTGAAGCCGTCCGA   240
HM585513.2       TCGGGTCTACTACGAGTCGGTGCGGACACTACGATCTCGAAGCCATCTGAAGCCGTCCGA   240
MH999839.1       TCGGGTCTACTACGAGTCGGTGCGGACACTACGATCTCGAAGCCATCTGAAGCCGTCCGA   240
MH999840.1       TCGGGTCTACTACGAGTCGGTGCGGACACTACGATCTCGAAGCCATCTGAAGCCGTCCGA   240
                 ***************.*********.**********************************
```

```
NC_001806.2        CCGCCAACAATCCCCAGGACACCGCGTGTTCCCCGGGAGCCCCGGGTTCCGCGACCACCC    300
MN136524.1         CCGCCAACAATCCCCAGGACACCGCGTGTTCCCCGGGAGCCCCGGGTTCCGCGACCACCC    300
KX791954.1         CCGCCAACAATCCCCAGGACACCGCGTGTTCCCCGGGAGCCCCGGGTTCCGCGACCACCC    300
KX791808.1         CCGCCAACAATCCCCAGGACACCGCGTGTTCCCCGGGAGCCCCGGGTTCCGCGACCACCC    300
KT425109.1         CCGCCAACAATCCCCAGGACACCGCGTGTTCCCCGGGAGCCCCGGGTTCCGCGACCACCC    300
ON007141.1         CCGCCAACAATCCCCAGGACACCGCGTGTTCCCCGGGAGCCCCGGGTTCCGCGACCACCC    300
ON007134.1         CCGCCAACAATCCCCAGGACACCGCGTGTTCCCCGGGAGCCCCGGGTTCCGCGACCACCC    300
ON007145.1         CCGCCAACAATCCCCAGGACACCGCGTGTTCCCCGGGAGCCCCGGGTTCCGCGACCACCC    300
OP297874.1         CCGCCAACAATCCCCAGGACACCGCGTGTTCCCCGGGAGCCCCGGGTTCCGCGACCACCC    300
OP297882.1         CCGCCAACAATCCCCAGGACACCGCGTGTTCCCCGGGAGCCCCGGGTTCCGCGACCACCC    300
HM585498.2         CCGCCAACAATCCCCAGGACACCGCGTGTTCCCCGGGAGCCCCGGGTTCCGCGACCACCC    300
ON007160.1         CCGCCAACAATCCCCAGGACACCGCGTGTTCCCCGGGAGCCCCGGGTTCCGCGACCACCC    300
HM585514.2         CCGCCAACAATCCCCAGGACACCGCGTGTTCCCCGGGAGCCCCGGGTTCCGCGACCACCC    300
OP297867.1         CCGCCAACAATCCCCAGGACACCGCGTGTTCCCCGGGAGCCCCGGGTTCCGCGACCACCC    300
LT594109.1         CCGCCAACAATCCCCAGGACACCGCGTGTTCCCCGGGAGCCCCGGGTTCCGCGACCACCC    300
LT594112.1         CCGCCAACAATCCCCAGGACACCGCGTGTTCCCCGGGAGCCCCGGGTTCCGCGACCACCC    300
HM585511.2         CCGCCAACAATCCCCAGGACACCGCGTGTTCCCCGGGAGCCCCGGGTTCCGCGACCACCC    300
KF498959.1         CCGCCAACAATCCCCAGGACACCGCGTGTTCCCCGGGAGCCCCGGGTTCCGCGACCACCC    300
MG999888.1         CCGCCAACAATCCCCAGGACACCGCGTGTTCCCCGGGAGCCCCGGGTTCCGCGACCACCC    300
ON960058.1         CCGCCAACAATCCCCAGGACACCGCGTGTTCCCCGGGAGCCCCGGGTTCCGCGACCACCC    300
MH160359.1         CCGCCAACAATCCCCAGGACACCGCGTGTTCCCCGGGAGCCCCGGGTTCCGCGACCACCC    300
MG999885.1         CCGCCAACAATCCCCAGGACACCGCGTGTTCCCCGGGAGCCCCGGGTTCCGCGACCACCC    300
MH999844.1         CCGCCAACAATCCCCAGGACACCGCGTGTTCCCCGGGAGCCCCGGGTTCCGCGACCACCC    300
MH160361.1         CCGCCAACAATCCCCAGGACACCGCGTGTTCCCCGGGAGCCCCGGGTTCCGCGACCACCC    300
MG999865.1         CCGCCAACAATCCCCAGGACACCGCGTGTTCCCCGGGAGCCCCGGGTTCCGCGACCACCC    300
MG999876.1         CCGCCAACAATCCCCAGGACACCGCGTGTTCCCCGGGAGCCCCGGGTTCCGCGACCACCC    300
GU734771.1         CCGCCAACAATCCCCAGGACACCGCGTGTTCCCCGGGAGCCCCGGGTTCCGCGACCACCC    300
MG999869.1         CCGCCAACAATCCCCAGGACACCGCGTGTTCCCCGGGAGCCCCGGGTTCCGCGACCACCC    300
MG999878.1         CCGCCAACAATCCCCAGGACACCGCGTGTTCCCCGGGAGCCCCGGGTTCCGCGACCACCC    300
ON960060.1         CCGCCAACAATCCCCAGGACACCGCGTGTTCCCCGGGAGCCCCGGGTTCCGCGACCACCC    300
MH999843.1         CCGCCAACAATCCCCAGGACACCGCGTGTTCCCCGGGAGCCCCGGGTTCCGCGACCACCC    300
MH999850.1         CCGCCAACAATCCCCAGGACACCGCGTGTTCCCCGGGAGCCCCGGGTTCCGCGACCACCC    300
MG999861.1         CCGCCAACAATCCCCAGGACACCGCGTGTTCCCCGGGAGCCCCGGGTTCCGCGACCACCC    300
KX791871.1         CCGCCAAAAATCCCCAGGACACCGCGTGTTCCCCGGGAGCCCCGGGTTCCGCGACCACCC    300
OP297861.1         CCGCCAACAATCCCCAGGACACCGCGTGTTCCCCGGGAGCCCCGGGTTCCGCGACCACCC    300
MG999864.1         CCGCCAACATTCCCCAGGACACCGCGTGTTCCCCGGGAGCCCCGGGTTCCGCGACCACCC    300
OL638990.1         CCGCCAACAATCCCCAGGACACCGCGTGTTCCCCGGGAGCCCCGGGTTCCGCGACCACCC    300
MH999838.1         CCGCCAACAATCCCCAGGACACCGCGTGTTCCCCGGGAGCCCAGGGTTCCGCGACCACCC    300
KU310659.1         CCGCCAACAATCCCCAGGACACCGCGTGTTCCCCGGGAGCCCCGGGTTCCGCGACCACCC    300
KX791923.1         CCGCCAACAATCCCCAGGACACCGCGTGTTCCCCGGGAGCCCCGGGTTCCGCGACCACCC    300
MG999845.1         CCGCCAACAATCCCCAGGACACCGCGTGTTCCCCGGGAGTCCCGGGTTCCGCGACCACCC    300
ON007132.1         CCGCCAACAATCCCCAGGACACCGCGTGTTCCCCGGGAGCCCCGGGTTCCGCGACCACCC    300
HM585497.2         CCGCCAACAATCCCCAGGACACCGCGTGTTCCCCGGGAGTCCCGGGTTCCGCGACCACCC    300
HM585500.2         CCGCCAACAATCCCCAGGACACCGCGTGTTCCCCGGGAGTCCCGGGTTCCGCGACCACCC    300
HM585499.2         CCGCCAACAATCCCCAGGACACCGCGTGTTCCCCGGGAGTCCCGGGTTCCGCGACCACCC    300
HM585504.2         CCGCCAACAATCCCCAGGACACCGCGTGTTCCCCGGGAGTCCCGGGTTCCGCGACCACCC    300
HM585496.2         CCGCCAACAATCCCCAGGACACCGCGTGTTCCCCGGGAGTCCCGGGTTCCGCGACCACCC    300
HM585506.2         CCGCCAACAATCCCCAGGACACCGCGTGTTCCCCGGGAGTCCCGGGTTCCGCGACCACCC    300
ON007139.1         CCGCCAACAATCCCCAGGACACCGCGTGTTCCCCGGGAGCCCCGGGTTCCGCGACCACCC    300
HM585509.2         CCGCCAACATTCCCCAGGACACCGCGTGTTCCCCGGGAGCCCCGGGTTCCGCGACCACCC    300
OP297883.1         CCGCCAACATTCCCCAGGACGCCGCGTGTTCCCCGGGAGCCCCGGGTTCCGCGACCACCC    300
MN136523.1         CCGCCAACATTCCCCAGGACACCGCGTGTTCCCCGGGAGCCCCGGGTTCCGCGACCACCC    300
MH999841.1         CCGCCAACAATCCCCAGGACACCGCGTGTTCCCCGGGAGCCCAGGGTTCCGCGACCACCC    300
MH813983.1         CCGCCAACATTCCCCAGGACACCGCGTGTTCCCCGGGAGCCCCGGGTTCCGCGACCACCC    300
MH813990.1         CCGCCAACATTCCCCAGGACACCGCGTGTTCCCCGGGAGCCCCGGGTTCCGCGACCACCC    300
MH813994.1         CCGCCAACATTCCCCAGGACACCGCGTGTTCCCCGGGAGCCCCGGGTTCCGCGACCACCC    300
MH813989.1         CCGCCAACATTCCCCAGGACACCGCGTGTTCCCCGGGAGCCCCGGGTTCCGCGACCACCC    300
MH813985.1         CCGCCAACATTCCCCAGGACACCGCGTGTTCCCCGGGAGCCCCGGGTTCCGCGACCACCC    300
MH813987.1         CCGCCAACATTCCCCAGGACACCGCGTGTTCCCCGGGAGCCCCGGGTTCCGCGACCACCC    300
MH813993.1         CCGCCAACATTCCCCAGGACACCGCGTGTTCCCCGGGAGCCCCGGGTTCCGCGACCACCC    300
MH813992.1         CCGCCAACATTCCCCAGGACACCGCGTGTTCCCCGGGAGCCCCGGGTTCCGCGACCACCC    300
MH813988.1         CCGCCAACATTCCCCAGGACACCGCGTGTTCCCCGGGAGCCCCGGGTTCCGCGACCACCC    300
MH813984.1         CCGCCAACATTCCCCAGGACACCGCGTGTTCCCCGGGAGCCCCGGGTTCCGCGACCACCC    300
MH813982.1         CCGCCAACATTCCCCAGGACACCGCGTGTTCCCCGGGAGCCCCGGGTTCCGCGACCACCC    300
OP297864.1         CCGCCAACAATCCCCAGGACACCGCGTGTTCCCCGGGAGCCCCGGGTTCCGCGACCACCC    300
MH813991.1         CCGCCAACATTCCCCAGGACACCGCGTGTTCCCCGGGAGCCCCGGGTTCCGCGACCACCC    300
MH813986.1         CCGCCAACATTCCCCAGGACACCGCGTGTTCCCCGGGAGCCCCGGGTTCCGCGACCACCC    300
HM585512.2         CCGCCAACAATCCCCAGGACACCGCGTGTTCCCCGGGAGCCCCGGGTTCCGCGACCACCC    300
HM585515.2         CCGCCAACAATCCCCAGGACACCGCGTGTTCCCCGGGAGCCCCGGGTTCCGCGACCACCC    300
HM585513.2         CCGCCAACAATCCCCAGGACACCGCGTGTTCCCCGGGAGCCCCGGGTTCCGCGACCACCC    300
MH999839.1         CCGCCAACAATCCCCAGGACACCGCGTGTTCCCCGGGAGCCCCGGGTTCCGCGACCACCC    300
MH999840.1         CCGCCAACAATCCCCAGGACACCGCGTGTTCCCCGGGAGCCCCGGGTTCCGCGACCACCC    300
                   *******.*:**********.****************** **.*****************
```

```
NC_001806.2        CGAGAACCTAGGGAACCCAGAGTACCGCGAGCTCCCAGAGACCCCAGGGTACCGCGTGAC    360
MN136524.1         CGAGAACCTAGGGAACCCAGAGTACCGCGAGCTCCCAGAGACCCCAGGGTACCGCGTGAC    360
KX791954.1         CGAGAACCTAGGGAACCCAGAGTACCGCGAGCTCCCAGAGACCCCAGGGTACCGCGTGAC    360
KX791808.1         CGAGAACCTAGGGAACCCAGAGTACCGCGAGCTCCCAGAGACCCCAGGGTACCGCGTGAC    360
KT425109.1         CGAGAACCTAGGGAACCCAGAGTACCGCGAGCTCCCAGAGACCCCAGGGTACCGCGTGAC    360
ON007141.1         CGAGAACCTAGGGAACCCAGAGTACCGCGAGCTCCCAGAGACCCCAGGGTACCGCGTGAC    360
ON007134.1         CGAGAACCTAGGGAACCCAGAGTACCGCGAGCTCCCAGAGACCCCAGGGTACCGCGTGAC    360
ON007145.1         CGAGAACCTAGGGAACCCAGAGTACCGCGAGCTCCCAGAGACCCCAGGGTACCGCGTGAC    360
OP297874.1         CGAGAACCTAGGGAACCCAGAGTACCGCGAGCTCCCAGAGACCCCAGGGTACCGCGTGAC    360
OP297882.1         CGAGAACCTAGGGAACCCAGAGTACCGCGAGCTCCCAGAGACCCCAGGGTACCGCGTGAC    360
HM585498.2         CGAGAACCTAGGGAACCCAGAGTACCGCGAGCTCCCAGAGACCCCAGGGTACCGCGTGAC    360
ON007160.1         CGAGAACCTAGGGAACCCAGAGTACCGCGAGCTCCCAGAGACCCCAGGGTACCGCGTGAC    360
HM585514.2         CGAGAACCTAGGGAACCCAGAGTACCGCGAGCTCCCAGAGACCCCAGGGTACCGCGTGAC    360
OP297867.1         CGAGAACCTAGGGAACCCAGAGTACCGCGAGCTCCCAGAGACCCCAGGGTACCGCGTGAC    360
LT594109.1         CGAGAACCTAGGGAACCCAGAGTACCGCGAGCTCCCAGAGACCCCAGGGTACCGCGTGAC    360
LT594112.1         CGAGAACCTAGGGAACCCAGAGTACCGCGAGCTCCCAGAGACCCCAGGGTACCGCGTGAC    360
HM585511.2         CGAGAACCTAGGGAACCCAGAGTACCGCGAGCTCCCAGAGACCCCAGGGTACCGCGTGAC    360
KF498959.1         CGAGAACCTAGGGAACCCAGAGTACCGCGAGCTCCCAGAGACCCCAGGGTACCGCGTGAC    360
MG999888.1         CGAGAACCTAGGGAACCCAGAGTACCGCGAGCTCCCAGAGACCCCAGGGTACCGCGTGAC    360
ON960058.1         CGAGAACCTAGGGAACCCAGAGTACCGCGAGCTCCCAGAGACCCCAGGGTACCGCGTGAC    360
MH160359.1         CGAGAACCTAGGGAACCCAGAGTACCGCGAGCTCCCAGAGACCCCAGGGTACCGCGTGAC    360
MG999885.1         CGAGAACCTAGGGAACCCAGAGTACCGCGAGCTCCCAGAGACCCCAGGGTACCGCGTGAC    360
MH999844.1         CGAGAACCTAGGGAACCCAGAGTACCGCGAGCTCCCAGAGACCCCAGGGTACCGCGTGAC    360
MH160361.1         CGAGAACCTAGGGAACCCAGAGTACCGCGAGCTCCCAGAGACCCCAGGGTACCGCGTGAC    360
MG999865.1         CGAGAACCTAGGGAACCCAGAGTACCGCGAGCTCCCAGAGACCCCAGGGTACCGCGTGAC    360
MG999876.1         CGAGAACCTAGGGAACCCAGAGTACCGCGAGCTCCCAGAGACCCCAGGGTACCGCGTGAC    360
GU734771.1         CGAGAACCTAGGGAACCCAGAGTACCGCGAGCTCCCAGAGACCCCAGGGTACCGCGTGAC    360
MG999869.1         CGAGAACCTAGGGAACCCAGAGTACCGCGAGCTCCCAGAGACCCCAGGGTACCGCGTGAC    360
MG999878.1         CGAGAACCTAGGGAACCCAGAGTACCGCGAGCTCCCAGAGACCCCAGGGTACCGCGTGAC    360
ON960060.1         CGAGAACCTAGGGAACCCAGAGTACCGCGAGCTCCCAGAGACCCCAGGGTACCGCGTGAC    360
MH999843.1         CGAGAACCTAGGGAACCCAGAGTACCGCGAGCTCCCAGAGACCCCAGGGTACCGCGTGAC    360
MH999850.1         CGAGAACCTAGGGAACCCAGAGTACCGCGAGCTCCCAGAGACCCCAGGGTACCGCGTGAC    360
MG999861.1         CGAGAACCTAGGGAACCCAGAGTACCGCGAGCTCCCAGAGACCCCAGGGTACCGCGTGAC    360
KX791871.1         CGAGAACCTAGGGAACCCAGAGTACCGCGAGCTCCCAGAGACCCCAGGGTACCGCGTGAC    360
OP297861.1         CGAGAACCTAGGGAACCCAGAGTACCGCGAGCTCCCAGAGACCCCAGGGTACCGCGTGAC    360
MG999864.1         CGAGAACCTAGGGAACCCAGAGTACCGCGAGCTCCCAGAGACCCCAGGGTACCGCGTGAC    360
OL638990.1         CGAGAACCTAGGGAACCCAGAGTACCGCGAGCTCCCAGAGACCCCAGGGTACCGCGTGAC    360
MH999838.1         CGAGAACCTAGGGAACCCAGAGTACCGCGAGCTCCCAGAGACCCCAGGGTACCTCGTGAC    360
KU310659.1         CGAGAACCTAGGGAACCCAGAGTACCGCGAGCTCCCAGAGACCCCAGGGTACCGCGTGAC    360
KX791923.1         CGAGAACCTAGGGAACCCAGAGTACCGCGAGCTCCCAGAGACCCCAGGGTACCGCGTGAC    360
MG999845.1         CGAGAACCTAGGGAACCCAGAGTACCGCGAGCTCCCAGAGACCCCAGGGTACCGCGTGAC    360
ON007132.1         CGAGAACCTAGGGAACCCAGAGTACCGCGAGCTCCCAGAGACCCCAGGGTACCGCGTGAC    360
HM585497.2         CGAGAACCTAGGGAACCCAGAGTACCGCGAGCTCCCAGAGACCCCAGGGTACCGCGTGAC    360
HM585500.2         CGAGAACCTAGGGAACCCAGAGTACCGCGAGCTCCCAGAGACCCCAGGGTACCGCGTGAC    360
HM585499.2         CGAGAACCTAGGGAACCCAGAGTACCGCGAGCTCCCAGAGACCCCAGGGTACCGCGTGAC    360
HM585504.2         CGAGAACCTAGGGAACCCAGAGTACCGCGAGCTCCCAGAGACCCCAGGGTACCGCGTGAC    360
HM585496.2         CGAGAACCTAGGGAACCCAGAGTACCGCGAGCTCCCAGAGACCCCAGGGTACCGCGTGAC    360
HM585506.2         CGAGAACCTAGGGAACCCAGAGTACCGCGAGCTCCCAGAGACCCCAGGGTACCGCGTGAC    360
ON007139.1         CGAGAACCTAGGGAACCCAGAGTACCGCGAGCTCCCAGAGACCCCAGGGTACCGCGTGAC    360
HM585509.2         CGAGAACCTAGGGAACCCAGAGTACCGCGAGCTCCCAGAGACCCCAGGGTACCGCGTGAC    360
OP297883.1         CGAGAACCTAGGGAACCCAGAGTACCGCGAGCTCCCAGAGACCCCAGGGTACCGCGTGAC    360
MN136523.1         CGAGAACCTAGGGAACCCAGAGTACCGCGAGCTCCCAGAGACCCCAGGGTACCGCGTGAC    360
MH999841.1         CGAGAACCTAGGGAACCCAGAGTACCGCGAGCTCCCAGAGACCCCAGGGTACCTCGTGAC    360
MH813983.1         CGAGAACCTAGGGAACCCAGAGTACCGCGAGCTCCCAGAGACCCCAGGGTACCGCGTGAC    360
MH813990.1         CGAGAACCTAGGGAACCCAGAGTACCGCGAGCTCCCAGAGACCCCAGGGTACCGCGTGAC    360
MH813994.1         CGAGAACCTAGGGAACCCAGAGTACCGCGAGCTCCCAGAGACCCCAGGGTACCGCGTGAC    360
MH813989.1         CGAGAACCTAGGGAACCCAGAGTACCGCGAGCTCCCAGAGACCCCAGGGTACCGCGTGAC    360
MH813985.1         CGAGAACCTAGGGAACCCAGAGTACCGCGAGCTCCCAGAGACCCCAGGGTACCGCGTGAC    360
MH813987.1         CGAGAACCTAGGGAACCCAGAGTACCGCGAGCTCCCAGAGACCCCAGGGTACCGCGTGAC    360
MH813993.1         CGAGAACCTAGGGAACCCAGAGTACCGCGAGCTCCCAGAGACCCCAGGGTACCGCGTGAC    360
MH813992.1         CGAGAACCTAGGGAACCCAGAGTACCGCGAGCTCCCAGAGACCCCAGGGTACCGCGTGAC    360
MH813988.1         CGAGAACCTAGGGAACCCAGAGTACCGCGAGCTCCCAGAGACCCCAGGGTACCGCGTGAC    360
MH813984.1         CGAGAACCTAGGGAACCCAGAGTACCGCGAGCTCCCAGAGACCCCAGGGTACCGCGTGAC    360
MH813982.1         CGAGAACCTAGGGAACCCAGAGTACCGCGAGCTCCCAGAGACCCCAGGGTACCGCGTGAC    360
OP297864.1         CGAGAACCTAGGGAACCCAGAGTACCGCGAGCTCCCAGAGACCCCAGGGTACCGCGTGAC    360
MH813991.1         CGAGAACCTAGGGAACCCAGAGTACCGCGAGCTCCCAGAGACCCCAGGGTACCGCGTGAC    360
MH813986.1         CGAGAACCTAGGGAACCCAGAGTACCGCGAGCTCCCAGAGACCCCAGGGTACCGCGTGAC    360
HM585512.2         CGAGAACCTAGGGAACCCAGAGTACCGCGAGCTCCCAGAGACCCCAGGGTACCGCGTGAC    360
HM585515.2         CGAGAACCTAGGGAACCCAGAGTACCGCGAGCTCCCAGAGACCCCAGGGTACCGCGTGAC    360
HM585513.2         CGAGAACCTAGGGAACCCAGAGTACCGCGAGCTCCCAGAGACCCCAGGGTACCGCGTGAC    360
MH999839.1         CGAGAACCTAGGGAACCCAGAGTACCGCGAGCTCCCAGAGACCCCAGGGTACCGCGTGAC    360
MH999840.1         CGAGAACCTAGGGAACCCAGAGTACCGCGAGCTCCCAGAGACCCCAGGGTACCGCGTGAC    360
                   ***************************************************** ******
```

```
NC_001806.2    CCCAGGGATCCACGACAACCCCGGTCTCCCAGGGAGCCCCGGTCTCCCCGGGAGCCCCGG    420
MN136524.1     CCCAGGGATCCACGACAACCCCG---------------------------GGAGCCCCGG    393
KX791954.1     CCCAGGGATCCACGACAACCCCG---------------------------GGAGCCCCGG    393
KX791808.1     CCCAGGGATCCACGACAACCCCG---------------------------GGAGCCCCGG    393
KT425109.1     CCCAGGGATCCACGACAACCCCG---------------------------GGAGCCCCGG    393
ON007141.1     CCCAGGGATCCACGACAACCCCG---------------------------GGAGCCCCGG    393
ON007134.1     CCCAGGGATCCACGACAACCCCG---------------------------GGAGCCCCGG    393
ON007145.1     CCCAGGGATCCACGACAACCCCG---------------------------GGAGCCCCGG    393
OP297874.1     CCCAGGGATCCACGACAACCCCG---------------------------GGAGCCCCGG    393
OP297882.1     CCCAGGGATCCACGACAACCCCG---------------------------GGAGCCCCGG    393
HM585498.2     CCCAGGGATCCACGACAACCCCG---------------------------GGAGCCCCGG    393
ON007160.1     CCCAGGGATCCACGACAACCCCG---------------------------GGAGCCCCGG    393
HM585514.2     CCCAGGGATCCACGACAACCCCG---------------------------GGAGCCCCGG    393
OP297867.1     CCCAGGGATCCACGACAACCCCG---------------------------GGAGCCCCGG    393
LT594109.1     CCCAGGGATCCACGACAACCCCG---------------------------GGAGCCCCGG    393
LT594112.1     CCCAGGGATCCACGACAACCCCG---------------------------GGAGCCCCGG    393
HM585511.2     CCCAGGGATCCACGACAACCCCG---------------------------GGAGCCCCGG    393
KF498959.1     CCCAGGGATCCACGACAACCCCG---------------------------GGAGCCCCGG    393
MG999888.1     CCCAGGGATCCACGACAACCCCG---------------------------GGAGCCCCGG    393
ON960058.1     CCCAGGGATCCACGACAACCCCG---------------------------GGAGCCCCGG    393
MH160359.1     CCCAGGGATCCACGACAACCCCG---------------------------GGAGCCCCGG    393
MG999885.1     CCCAGGGATCCACGACAACCCCG---------------------------GGAGCCCCGG    393
MH999844.1     CCCAGGGATCCACGACAACCCCG---------------------------GGAGCCCCGG    393
MH160361.1     CCCAGGGATCCACGACAACCCCG---------------------------GGAGCCCCGG    393
MG999865.1     CCCAGGGATCCACGACAACCCCG---------------------------GGAGCCCCGG    393
MG999876.1     CCCAGGGATCCACGACAACCCCG---------------------------GGAGCCCCGG    393
GU734771.1     CCCAGGGATCCACGACAACCCCG---------------------------GGAGCCCCGG    393
MG999869.1     CCCAGGGATCCACGACAACCCCG---------------------------GGAGCCCCGG    393
MG999878.1     CCCAGGGATCCACGACAACCCCG---------------------------GGAGCCCCGG    393
ON960060.1     CCCAGGGATCCACGACAACCCCG---------------------------GGAGCCCCGG    393
MH999843.1     CCCAGGGATCCACGACAACCCCG---------------------------GGAGCCCCGG    393
MH999850.1     CCCAGGGATCCACGACAACCCCG---------------------------GGAGCCCCGG    393
MG999861.1     CCCAGGGATCCACGACAACCCCG---------------------------GGAGCCCCGG    393
KX791871.1     CCCAGGGATCCACGACAACCCCG---------------------------GGAGCCCCGG    393
OP297861.1     CCCAGGGATCCACGACAACCCCG---------------------------GGAGCCCCGG    393
MG999864.1     CCCAGGGATCCACGACAACCCCG---------------------------GGAGCCCCGG    393
OL638990.1     CCCAGGGATCCACGACAACCCCG---------------------------GGAGCCCCGG    393
MH999838.1     CCCAGGGATCCACGACAACCCCG---------------------------GGAGCCCCGG    393
KU310659.1     CCCAGGGATCCACGACAACCCCG---------------------------GGAGCCCCGG    393
KX791923.1     CCCAGGGATCCACGACAACCCCG---------------------------GGAGCCCCGG    393
MG999845.1     CCCAGGGATCCACGACAACCCCG---------------------------GGAGCCCCGG    393
ON007132.1     CCCAGGGATCCACGACAACCCCG---------------------------GGAGCCCCGG    393
HM585497.2     CCCAGGGATCCACGACAACCCCG---------------------------GGAGCCCCGG    393
HM585500.2     CCCAGGGATCCACGACAACCCCG---------------------------GGAGCCCCGG    393
HM585499.2     CCCAGGGATCCACGACAACCCCG---------------------------GGAGCCCCGG    393
HM585504.2     CCCAGGGATCCACGACAACCCCG---------------------------GGAGCCCCGG    393
HM585496.2     CCCAGGGATCCACGACAACCCCG---------------------------GGAGCCCCGG    393
HM585506.2     CCCAGGGATCCACGACAACCCCG---------------------------GGAGCCCCGG    393
ON007139.1     CCCAGGGATCCACGACAACCCCG---------------------------GGAGCCCCGG    393
HM585509.2     CCCAGGGATCCACGACAACCCCG---------------------------GGAGCCCCGG    393
OP297883.1     CCCAGGGATCCACGACAACCCCG---------------------------GGAGCCCCGG    393
MN136523.1     CCCAGGGATCCACGACAACCCCG---------------------------GGAGCCCCGG    393
MH999841.1     CCCAGGGATCCACGACAACCCCG---------------------------GGAGCCCCGG    393
MH813983.1     CCCAGGGATCCACGACAACCCCG---------------------------GGAGCCCCGG    393
MH813990.1     CCCAGGGATCCACGACAACCCCG---------------------------GGAGCCCCGG    393
MH813994.1     CCCAGGGATCCACGACAACCCCG---------------------------GGAGCCCCGG    393
MH813989.1     CCCAGGGATCCACGACAACCCCG---------------------------GGAGCCCCGG    393
MH813985.1     CCCAGGGATCCACGACAACCCCG---------------------------GGAGCCCCGG    393
MH813987.1     CCCAGGGATCCACGACAACCCCG---------------------------GGAGCCCCGG    393
MH813993.1     CCCAGGGATCCACGACAACCCCG---------------------------GGAGCCCCGG    393
MH813992.1     CCCAGGGATCCACGACAACCCCG---------------------------GGAGCCCCGG    393
MH813988.1     CCCAGGGATCCACGACAACCCCG---------------------------GGAGCCCCGG    393
MH813984.1     CCCAGGGATCCACGACAACCCCG---------------------------GGAGCCCCGG    393
MH813982.1     CCCAGGGATCCACGACAACCCCG---------------------------GGAGCCCCGG    393
OP297864.1     CCCAGGGATCCACGACAACCCCG---------------------------GGAGCCCCGG    393
MH813991.1     CCCAGGGATCCACGACAACCCCG---------------------------GGAGCCCCGG    393
MH813986.1     CCCAGGGATCCACGACAACCCCG---------------------------GGAGCCCCGG    393
HM585512.2     CCCAGGGATCCACGACAACCCCG---------------------------GGAGCCCCGG    393
HM585515.2     CCCAGGGATCCACGACAACCCCG---------------------------GGAGCCCCGG    393
HM585513.2     CCCAGGGATCCACGACAACCCCG---------------------------GGAGCCCCGG    393
MH999839.1     CCCAGGGATCCACGACAACCCCG---------------------------GGAGCCCCGG    393
MH999840.1     CCCAGGGATCCACGACAACCCCG---------------------------GGATCCACGA    393
               *********************                             *** **.**.
```

```
NC_001806.2      TCTCCCCGGGAGCCCCGGACCCCACGCACCCCCCGCGAACCACGTACGGCTCGCGGGTCT   480
MN136524.1       CCTCCCCGGGAGCCCCGGACCCCACGCACCCCCCGCGAACCACGTACGGCTCGCGGGTCT   453
KX791954.1       CCTCCCCGGGAGCCCCGGACCCCACGCACCCCCCGCGAACCACGTACGGCTCGCGGGTCT   453
KX791808.1       CCTCCCCGGGAGCCCCGGACCCCACGCACCCCCCGCGAACCACGTACGGCTCGCGGGTCT   453
KT425109.1       CCTCCCCGGGAGCCCCGGACCCCACGCACCCCCCGCGAACCACGTACGGCTCGCGGGTCT   453
ON007141.1       CCTCCCCGGGAGCCCCGGACCCCACGCACCCCCCGCGAACCACGTACGGCTCGCGGGTCT   453
ON007134.1       CCTCCCCGGGAGCCCCGGACCCCACGCACCCCCCGCGAACCACGTACGGCTCGCGGGTCT   453
ON007145.1       CCTCCCCGGGAGCCCCGGACCCCACGCACCCCCCGCGAACCACGTACGGCTCGCGGGTCT   453
OP297874.1       CCTCCCCGGGAGCCCCGGACCCCACGCACCCCCCGCGAACCACGTACGGCTCGCGGGTCT   453
OP297882.1       CCTCCCCGGGAGCCCCGGACCCCACGCACCCCCCGCGAACCACGTACGGCTCGCGGGTCT   453
HM585498.2       CCTCCCCGGGAGCCCCGGACCCCACGCACCCCCCGCGAACCACGTACGGCTCGCGGGTCT   453
ON007160.1       CCTCCCCGGGAGCCCCGGACCCCACGCACCCCCCGCGAACCACGTACGGCTCGCGGGTCT   453
HM585514.2       CCTCCCCGGGAGCCCCGGACCCCACGCACCCCCCGCGAACCACGTACGGCTCGCGGGTCT   453
OP297867.1       CCTCCCCGGGAGCCCCGGACCCCACGCACCCCCCGCGAACCACGTACGGCTCGCGGGTCT   453
LT594109.1       CCTCCCCGGGAGCCCCGGACCCCACGCACCCCCCGCGAACCACGTACGGCTCGCGGGTCT   453
LT594112.1       CCTCCCCGGGAGCCCCGGACCCCACGCACCCCCCGCGAACCACGTACGGCTCGCGGGTCT   453
HM585511.2       CCTCCCCGGGAGCCCCGGACCCCACGCACCCCCCGCGAACCACGTACGGCTCGCGGGTCT   453
KF498959.1       CCTCCCCGGGAGCCCCGGACCCCACGCACCCCCCGCGAACCACGTACGGCTCGCGGGTCT   453
MG999888.1       CCTCCCCGGGAGCCCCGGACCCCACGCACCCCCCGCGAACCACGTACGGCTCGCGGGTCT   453
ON960058.1       CCTCCCCGGGAGCCCCGGACCCCACGCACCCCCCGCGAACCACGTACGGCTCGCGGGTCT   453
MH160359.1       CCTCCCCGGGAGCCCCGGACCCCACGCACCCCCCGCGAACCACGTACGGCTCGCGGGTCT   453
MG999885.1       CCTCCCCGGGAGCCCCGGACCCCACGCACCCCCCGCGAACCACGTACGGCTCGCGGGTCT   453
MH999844.1       CCTCCCCGGGAGCCCCGGACCCCACGCACCCCCCGCGAACCACGTACGGCTCGCGGGTCT   453
MH160361.1       CCTCCCCGGGAGCCCCGGACCCCACGCACCCCCCGCGAACCACGTACGGCTCGCGGGTCT   453
MG999865.1       CCTCCCCGGGAGCCCCGGACCCCACGCACCCCCCGCGAACCACGTACGGCTCGCGGGTCT   453
MG999876.1       CCTCCCCGGGAGCCCCGGACCCCACGCACCCCCCGCGAACCACGTACGGCTCGCGGGTCT   453
GU734771.1       CCTCCCCGGGAGCCCCGGACCCCACGCACCCCCCGCGAACCACGTACGGCTCGCGGGTCT   453
MG999869.1       CCTCCCCGGGAGCCCCGGACCCCACGCACCCCCCGCGAACCACGTACGGCTCGCGGGTCT   453
MG999878.1       CCTCCCCGGGAGCCCCGGACCCCACGCACCCCCCGCGAACCACGTACGGCTCGCGGGTCT   453
ON960060.1       CCTCCCCGGGAGCCCCGGACCCCACGCACCCCCCGCGAACCACGTACGGCTCGCGGGTCT   453
MH999843.1       CCTCCCCGGGAGCCCCGGACCCCACGCACCCCCCGCGAACCACGTACGGCTCGCGGGTCT   453
MH999850.1       CCTCCCCGGGAGCCCCGGACCCCACGCACCCCCCGCGAACCACGTACGGCTCGCGGGTCT   453
MG999861.1       CCTCCCCGGGAGCCCCGGACCCCACGCACCCCCCGCGAACCACGTACGGCTCGCGGGTCT   453
KX791871.1       CCTCCCCGGGAGCCCCGGACCCCACGCACCCCCCGCGAACCACGTACGGCTCGCGGGTCT   453
OP297861.1       CCTCCCCGGGAGCCCCGGACCCCACGCACCCCCCGCGAACCACGTACGGCTCGCGGGTCT   453
MG999864.1       CCTCCCCGGGAGCCCCGGACCCCACGCACCCCCCGCGAACCACGTACGGCTCGCGGGTCT   453
OL638990.1       CCTCCCCGGGAGCCCCGGACCCCACGCACCCCCCGCGAACCACGTACGGCTCGCGGGTCT   453
MH999838.1       CCTCCCCGGGAGCCCCGGACCCCACGCACCCCCCGCGAACCACGTACGGCTCGCGGGTCT   453
KU310659.1       CCTCCCCGGGAGCCCCGGACCCCACGCACCCCCCGCGAACCACGTACGGCTCGCGGGTCT   453
KX791923.1       CCTCCCCGGGAGCCCCGGACCCCACGCACCCCCCGCGAACCACGTACGGCTCGCGGGTCT   453
MG999845.1       CCTCCCCGGGAGCCCCGGACCCCACGCACCCCCCGCGAACCACGTACGGCTCGCGGGTCT   453
ON007132.1       CCTCCCCGGGAGCCCCGGACCCCACGCACCCCCCGCGAACCACGTACGGCTCGCGGGTCT   453
HM585497.2       CCTCCCCGGGAGCCCCGGACCCCACGCACCCCCCGCGAACCACGTACGGCTCGCGGGTCT   453
HM585500.2       CCTCCCCGGGAGCCCCGGACCCCACGCACCCCCCGCGAACCACGTACGGCTCGCGGGTCT   453
HM585499.2       CCTCCCCGGGAGCCCCGGACCCCACGCACCCCCCGCGAACCACGTACGGCTCGCGGGTCT   453
HM585504.2       CCTCCCCGGGAGCCCCGGACCCCACGCACCCCCCGCGAACCACGTACGGCTCGCGGGTCT   453
HM585496.2       CCTCCCCGGGAGCCCCGGACCCCACGCACCCCCCGCGAACCACGTACGGCTCGCGGGTCT   453
HM585506.2       CCTCCCCGGGAGCCCCGGACCCCACGCACCCCCCGCGAACCACGTACGGCTCGCGGGTCT   453
ON007139.1       CCTCCCCGGGAGCCCCGGACCCCACGCACCCCCCGCGAACCACGTACGGCTCGCGGGTCT   453
HM585509.2       CCTCCCCGGGAGCCCCGGACCCCACGCACCCCCCGCGAACCACGTACGGCTCGCGGGTCT   453
OP297883.1       CCTCCCCGGGAGCCCCGGACCCCACGCACCCCCCGCGAACCACGTACGGCTCGCGGGTCT   453
MN136523.1       CCTCCCCGGGAGCCCCGGACCCCACGCACCCCCCGCGAACCACGTACGGCTCGCGGGTCT   453
MH999841.1       CCTCCCCGGGAGCCCCGGACCCCACGCACCCCCCGCGAACCACGTACGGCTCGCGGGTCT   453
MH813983.1       CCTCCCCGGGAGCCCCGGACCCCACGCACCCCCCGCGAACCACGTACGGCTCGCGGGTCT   453
MH813990.1       CCTCCCCGGGAGCCCCGGACCCCACGCACCCCCCGCGAACCACGTACGGCTCGCGGGTCT   453
MH813994.1       CCTCCCCGGGAGCCCCGGACCCCACGCACCCCCCGCGAACCACGTACGGCTCGCGGGTCT   453
MH813989.1       CCTCCCCGGGAGCCCCGGACCCCACGCACCCCCCGCGAACCACGTACGGCTCGCGGGTCT   453
MH813985.1       CCTCCCCGGGAGCCCCGGACCCCACGCACCCCCCGCGAACCACGTACGGCTCGCGGGTCT   453
MH813987.1       CCTCCCCGGGAGCCCCGGACCCCACGCACCCCCCGCGAACCACGTACGGCTCGCGGGTCT   453
MH813993.1       CCTCCCCGGGAGCCCCGGACCCCACGCACCCCCCGCGAACCACGTACGGCTCGCGGGTCT   453
MH813992.1       CCTCCCCGGGAGCCCCGGACCCCACGCACCCCCCGCGAACCACGTACGGCTCGCGGGTCT   453
MH813988.1       CCTCCCCGGGAGCCCCGGACCCCACGCACCCCCCGCGAACCACGTACGGCTCGCGGGTCT   453
MH813984.1       CCTCCCCGGGAGCCCCGGACCCCACGCACCCCCCGCGAACCACGTACGGCTCGCGGGTCT   453
MH813982.1       CCTCCCCGGGAGCCCCGGACCCCACGCACCCCCCGCGAACCACGTACGGCTCGCGGGTCT   453
OP297864.1       CCTCCCCGGGAGCCCCGGACCCCACGCACCCCCCGCGAACCACGTACGGCTCGCGGGTCT   453
MH813991.1       CCTCCCCGGGAGCCCCGGACCCCACGCACCCCCCGCGAACCACGTACGGCTCGCGGGTCT   453
MH813986.1       CCTCCCCGGGAGCCCCGGACCCCACGCACCCCCCGCGAACCACGTACGGCTCGCGGGTCT   453
HM585512.2       CCTCCCCGGGAGCCCCGGACCCCACGCACCCCCCGCAAACCACGTACGGCTCGCGGGTCT   453
HM585515.2       CCTCCCCGGGAGCCCCGGACCCCACGCACCCCCCGCAAACCACGTACGGCTCGCGGGTCT   453
HM585513.2       CCTCCCCGGGAGCCCCGGACCCCACGCACCCCCCGCAAACCACGTACGGCTCGCGGGTCT   453
MH999839.1       CCTCCCCGGGAGCCCCGGACCCCACGCACCCCCCGCAAACCACGTACGGCTCGCGGGTCT   453
MH999840.1       CAACCCCGGGAGCCCCGGACCCCACGCACCCCCCGCGAACCACGTACGGCTCGCGGGTCT   453
                 .:**********************************.***********************
```

NC_001806.2	GTATAG	486
MN136524.1	GTATAG	459
KX791954.1	GTATAG	459
KX791808.1	GTATAG	459
KT425109.1	GTATAG	459
ON007141.1	GTATAG	459
ON007134.1	GTATAG	459
ON007145.1	GTATAG	459
OP297874.1	GTATAG	459
OP297882.1	GTATAG	459
HM585498.2	GTATAG	459
ON007160.1	GTATAG	459
HM585514.2	GTATAG	459
OP297867.1	GTATAG	459
LT594109.1	GTATAG	459
LT594112.1	GTATAG	459
HM585511.2	GTATAG	459
KF498959.1	GTATAG	459
MG999888.1	GTATAG	459
ON960058.1	GTATAG	459
MH160359.1	GTATAG	459
MG999885.1	GTATAG	459
MH999844.1	GTATAG	459
MH160361.1	GTATAG	459
MG999865.1	GTATAG	459
MG999876.1	GTATAG	459
GU734771.1	GTATAG	459
MG999869.1	GTATAG	459
MG999878.1	GTATAG	459
ON960060.1	GTATAG	459
MH999843.1	GTATAG	459
MH999850.1	GTATAG	459
MG999861.1	GTATAG	459
KX791871.1	GTATAG	459
OP297861.1	GTATAG	459
MG999864.1	GTATAG	459
OL638990.1	GTATAG	459
MH999838.1	GTATAG	459
KU310659.1	GTATAG	459
KX791923.1	GTATAG	459
MG999845.1	GTATAG	459
ON007132.1	GTATAG	459
HM585497.2	GTATAG	459
HM585500.2	GTATAG	459
HM585499.2	GTATAG	459
HM585504.2	GTATAG	459
HM585496.2	GTATAG	459
HM585506.2	GTATAG	459
ON007139.1	GTATAG	459
HM585509.2	GTATAG	459
OP297883.1	GTATAG	459
MN136523.1	GTATAG	459
MH999841.1	GTATAG	459
MH813983.1	GTATAG	459
MH813990.1	GTATAG	459
MH813994.1	GTATAG	459
MH813989.1	GTATAG	459
MH813985.1	GTATAG	459
MH813987.1	GTATAG	459
MH813993.1	GTATAG	459
MH813992.1	GTATAG	459
MH813988.1	GTATAG	459
MH813984.1	GTATAG	459
MH813982.1	GTATAG	459
OP297864.1	GTATAG	459
MH813991.1	GTATAG	459
MH813986.1	GTATAG	459
HM585512.2	GTATAG	459
HM585515.2	GTATAG	459
HM585513.2	GTATAG	459
MH999839.1	GTATAG	459
MH999840.1	GTATAG	459
	******	
