# Supplementary material for: Discovery of a Novel Intron in US10/US11/US12 of HSV-1 Strain 17
Source: Viruses. 2023 Oct 25;15(11):2144. doi: 10.3390/v15112144 (PMC10675037; doi:10.3390/v15112144)
Supplement: Supplementary file 1 [file viruses-15-02144-s001.zip › Supplemental Data S3. 9-nt alignment.pdf]

Supplemental Data S3: Alignment of the HSV-1 US11 Gene 9 nt Shorter Than HSV-1 Strain 17

```
NC_001806.2      ATGAGCCAGACCCAACCCCCGGCCCCAGTTGGGCCGGGCGACCCAGATGTTTACTTAAAA      60
OP297871.1       ATGAGCCAGACCCAACCCCCGGCCCCAGTTGGGCCGGGCGACCCAGATGTTTACTTAAAA      60
OQ658624.1       ATGAGCCAGACCCAACCCCCGGCCCCAGTTGGGCCGGGCGACCCAGATGTTTACTTAAAA      60
MH160371.1       ATGAGCCAGACCCAACCCCCGGCCCCAGTTGGGCCGGGCGACCCAGATGTTTACTTAAAA      60
HM585507.2       ATGAGCCAGACCCAACCCCCGGCCCCAGTTGGGCCGGGCGACCCAGATGTTTACTTAAAA      60
HM585501.2       ATGAGCCAGACCCAACCCCCGGCCCCAGTTGGGCCGGGCGACCCAGATGTTTACTTAAAA      60
HM585503.2       ATGAGCCAGACCCAACCCCCGGCCCCAGTTGGGCCGGGCGACCCAGATGTTTACTTAAAA      60
HM585505.2       ATGAGCCAGACCCAACCCCCGGCCCCAGTTGGGCCGGGCGACCCAGATGTTTACTTAAAA      60
                 ************************************************************

NC_001806.2      GGCGTGCCGTCCGCCGGCATGCACCCCAGAGGTGTTCACGCACCTCGAGGACACCCGCGC      120
OP297871.1       GGCGTGCCGTCCGCCGGCATGCACCCCAGAGGTGTTCACGCACCTCGAGGACACCCGCGC      120
OQ658624.1       GGCGTGCCGTTCGCCGGCATGCACCCCAGAGGTGTTCACGCACCTCGAGGACACCCGCGC      120
MH160371.1       GGCGTGCCGTCCGCCGGCATGCACCCCAGAGGTGTTCACGCACCTCGAGGACACCCGCAC      120
HM585507.2       GGCGTGCCGTCCGCCGGCATGCACCCCAGAGGTGTTCACGCACCTCGAGGACACCCGCGC      120
HM585501.2       GGCGTGCCGTCCGCCGGCATGCACCCCAGAGGTGTTCACGCACCTCGAGGACACCCGCGC      120
HM585503.2       GGCGTGCCGTCCGCCGGCATGCACCCCAGAGGTGTTCACGCACCTCGAGGACACCCGCGC      120
HM585505.2       GGCGTGCCGTCCGCCGGCATGCACCCCAGAGGTGTTCACGCACCTCGAGGACACCCGCGC      120
                 ********** *********************************************** *

NC_001806.2      ATGATCTCCGGACCCCCGCAACGGGGTGATAATGATCAAGCGGCGGGGCAATGTGGAGAT      180
OP297871.1       ATGATCTCCGGACCCCCGCAACGGGGTGATAATGATCAAGCGGCGGGGCAATGTGGAGAT      180
OQ658624.1       ATGATCTCCGGACCCCCGCAACGGGGTGATAATGATCAAGCGGCGGGGCAATGTGGAGAT      180
MH160371.1       ATGATCTCCGGACCCCCGCAACGGGGTGATAATGATCAAGCGGCGGGGCAATGTGGAGAT      180
HM585507.2       ATGATCTCCGGACCCCCGCAACGGGGTGATAATGATCAAGCGGCGGGGCAATGTGGAGAT      180
HM585501.2       ATGATCTCCGGACCCCCGCAACGGGGTGATAATGATCAAGCGGCGGGGCAATGTGGAGAT      180
HM585503.2       ATGATCTCCGGACCCCCGCAACGGGGTGATAATGATCAAGCGGCGGGGCAATGTGGAGAT      180
HM585505.2       ATGATCTCCGGACCCCCGCAACGGGGTGATAATGATCAAGCGGCGGGGCAATGTGGAGAT      180
                 ************************************************************

NC_001806.2      TCGGGTCTACTACGAGTCGGTGCGGACACTACGATCTCGAAGCCATCTGAAGCCGTCCGA      240
OP297871.1       TCGGGTCTACTACGAGTCGGTGCGGACACTACGATCTCGAAGCCATCTGAAGCCGTCCGA      240
OQ658624.1       TCGGGTCTACTACGAGTCGGTGCGGACACTACGATCTCGAAGCCATCTGAAGCCGTCCGA      240
MH160371.1       TCGGGTCTACTACGAGTCGGTGCGGCCACTACGATCTCGAAGCCATCTGAAGCCGTCCGA      240
HM585507.2       TCGGGTCTACTACGAGTCGGTGCGGACACTACGATCTCGAAGCCATCTGAAGCCGTCCGA      240
HM585501.2       TCGGGTCTACTACGAGTCGGTGCGGACACTACGATCTCGAAGCCATCTGAAGCCGTCCGA      240
HM585503.2       TCGGGTCTACTACGAGTCGGTGCGGACACTACGATCTCGAAGCCATCTGAAGCCGTCCGA      240
HM585505.2       TCGGGTCTACTACGAGTCGGTGCGGACACTACGATCTCGAAGCCATCTGAAGCCGTCCGA      240
                 ************************* **********************************

NC_001806.2      CCGCCAACAATCCCCAGGACACCGCGTGTTCCCCGGGAGCCCCGGGTTCCGCGACCACCC      300
OP297871.1       CCGCCAACAATCCCCAGGACACCGCGTGTTCCCCGGGAGCCCCGGGTTCCGCGACCACCC      300
OQ658624.1       CCGCCAACATTCCCCAGGACACCGCGTGTTCCCCGGGAGCCCCGGGTTCCGCGACCACCC      300
MH160371.1       CCGCCAACAATCCCCAGGACACCGCGTGTTCCCCGGGAGCCCCGGGTTCCGCGACCACCC      300
HM585507.2       CCGCCAACATTCCCCAGGACGCCGCGTGTTCCCCGGGAGCCCCGGGTTCCGCGACCACCC      300
HM585501.2       CCGCCAACATTCCCCAGGACGCCGCGTGTTCCCCGGGAGCCCCGGGTTCCGCGACCACCC      300
HM585503.2       CCGCCAACATTCCCCAGGACGCCGCGTGTTCCCCGGGAGCCCCGGGTTCCGCGACCACCC      300
HM585505.2       CCGCCAACATTCCCCAGGACGCCGCGTGTTCCCCGGGAGCCCCGGGTTCCGCGACCACCC      300
                 ********* ********** ***************************************

NC_001806.2      CGAGAACCTAGGGAACCCAGAGTACCGCGAGCTCCCAGAGACCCCAGGGTACCGCGTGAC      360
OP297871.1       CGAGAACCTAGGGAACCCAGAGTACCGCGAGCTCCCAGAGACCCCAGGGTACCGCGTGAC      360
OQ658624.1       CGAGAACCTAGGGAACCCAGAGTACCGCGAGCTCCCAGAGACCCCAGGGTACCGCGTGAC      360
MH160371.1       CGAGAACCTAGGGAACCCAGAGTACCGCGAGCTCCCAGAGACCCCAGGGTACCGCGTGAC      360
HM585507.2       CGAGAACCTAGGGAACCCAGAGTACCGCGAGCTCCCAGAGACCCCAGGGTCCCGCGTGAC      360
HM585501.2       CGAGAACCTAGGGAACCCAGAGTACCGCGAGCTCCCAGAGACCCCAGGGTCCCGCGTGAC      360
HM585503.2       CGAGAACCTAGGGAACCCAGAGTACCGCGAGCTCCCAGAGACCCCAGGGTCCCGCGTGAC      360
HM585505.2       CGAGAACCTAGGGAACCCAGAGTACCGCGAGCTCCCAGAGACCCCAGGGTCCCGCGTGAC      360
                 ************************************************** *********
```

```
NC_001806.2  CCCAGGGATCCACGACAACCCCGGTCTCCCAGGGAGCCCCGGTCTCCCCGGGAGCCCCGG   420
OP297871.1   CCCAGGGATCCACGACAACCCCGGGA---------GCCCCGGCCTCCCCGGGAGCCCCGG   411
OQ658624.1   CCCAGGGATCCACGACAACCCCGGGA---------GCCCCGGCCTCCCCGGGAGCCCCGG   411
MH160371.1   CCCAGGGATCCACGACAACCCCGGGATCCACGACAA---------CCCCGGGAGCCCCGG   411
HM585507.2   CCCAGGGATCCACGACAACCCCGGGA---------GCCCCGGCCTCCCCGGGATCCACGA   411
HM585501.2   CCCAGGGATCCACGACAACCCCGGGA---------GCCCCGGCCTCCCCGGGATCCACGA   411
HM585503.2   CCCAGGGATCCACGACAACCCCGGGA---------GCCCCGGCCTCCCCGGGATCCACGA   411
HM585505.2   CCCAGGGATCCACGACAACCCCGGGA---------GCCCCGGCCTCCCCGGGATCCACGA   411
             ************************                     ******** ** **

NC_001806.2  TCTCCCCGGGAGCCCCGGACCCCACGCACCCCCCGCGAACCACGTACGGCTCGCGGGTCT   480
OP297871.1   CCTCCCCGGGAGCCCCGGACCCCACGCACCCCCCGCGAACCACGTACGGCTCGCGGGTCT   471
OQ658624.1   CCTCCCCGGGAGCCCCGGACCCCACGCACCCCCCGCGAACCACGTACGGCTCGCGGGTCT   471
MH160371.1   CCTCCCCGGGAGCCCCGGACCCCACGCACCCCCCGCGAACCACGTACGGCTCGCGGGTCT   471
HM585507.2   CAACCCCGGGAGCCCCGGACCCCACGCACCCCCCGCGAACCACGTACGGCTCGCGGGTCT   471
HM585501.2   CAACCCCGGGAGCCCCGGACCCCACGCACCCCCCGCGAACCACGTACGGCTCGCGGGTCT   471
HM585503.2   CAACCCCGGGAGCCCCGGACCCCACGCACCCCCCGCGAACCACGTACGGCTCGCGGGTCT   471
HM585505.2   CAACCCCGGGAGCCCCGGACCCCACGCACCCCCCGCGAACCACGTACGGCTCGCGGGTCT   471
                ********************************************************* 

NC_001806.2  GTATAG   486
OP297871.1   GTATAG   477
OQ658624.1   GTATAG   477
MH160371.1   GTATAG   477
HM585507.2   GTATAG   477
HM585501.2   GTATAG   477
HM585503.2   GTATAG   477
HM585505.2   GTATAG   477
             ******
```
