# Supplementary material for: Discovery of a Novel Intron in US10/US11/US12 of HSV-1 Strain 17
Source: Viruses. 2023 Oct 25;15(11):2144. doi: 10.3390/v15112144 (PMC10675037; doi:10.3390/v15112144)
Supplement: Supplementary file 1 [file viruses-15-02144-s001.zip › Supplemental Data S4. 18-nt alignment.pdf]

Supplemental Data S4: Alignment of the HSV-1 US11 Gene 18 nt Shorter Than HSV-1 Strain 17

```
NC_001806.2      ATGAGCCAGACCCAACCCCCGGCCCCAGTTGGGCCGGGCGACCCAGATGTTTACTTAAAA    60
OP297876.1       ATGAGCCAGACCCAACCCCCGGCCCCAGTTGGGCCGGGCGACCCAGATGTTTACTTAAAA    60
KT425108.1       ATGAGCCAGACCCAACCCCCGGCCCCAGTTGGGCCGGGCGACCCAGATGTTTACTTAAAA    60
OP297880.1       ATGAGCCAGACCCAACCCCCGGCCCCAGTTGGGCCGGGCGACCCAGATGTTTACTTAAAA    60
OP297875.1       ATGAGCCAGACCCAACCCCCGGCCCCAGTTGGGCCGGGCGACCCAGATGTTTACTTAAAA    60
ON007163.1       ATGAGCCAGACCCAACCCCCGGCCCCAGTTGGGCCGGGCGACCCAGATGTTTACTTAAAA    60
ON007155.1       ATGAGCCAGACCCAACCCCCGGCCCCAGTTGGGCCGGGCGACCCAGATGTTTACTTAAAA    60
OP297877.1       ATGAGCCAGACCCAACCCCCGGCCCCAGTTGGGCCGGGCGACCCAGATGTTTACTTAAAA    60
MN401201.1       ATGAGCCAGACCCAACCCCCGGCCCCAGTTGGGCCGGGCGACCCAGATGTTTACTTAAAA    60
ON960054.1       ATGAGCCAGACCCAACCCCCGGCCCCAGTTGGGCCGGGCGACCCAGATGTTTACTTAAAA    60
ON007144.1       ATGAGCCAGACCCAACCCCCGGCCCCAGTTGGGCCGGGCGACCCAGATGTTTACTTAAAA    60
LT594457.1       ATGAGCCAGACCCAACCCCCGGCCCCAGTTGGGCCGGGCGACCCAGATGTTTACTTAAAA    60
LT594122.1       ATGAGCCAGACCCAACCCCCGGCCCCAGTTGGGCCGGGCGACCCAGATGTTTACTTAAAA    60
OP297863.1       ATGAGCCAGACCCAACCCCCGGCCCCAGTTGGGCCGGGCGACCCAGATGTTTACTTAAAA    60
ON152716.1       ATGAGCCAGACCCAACCCCCGGCCCCAGTTGGGCCGGGCGACCCAGATGTTTACTTAAAA    60
MH160374.1       ATGAGCCAGACCCAACCCCCGGCCCCAGTTGGGCCGGGCGACCCAGATGTTTACTTAAAA    60
MH999846.1       ATGAGCCAGACCCAACCCCCGGCCCCAGTTGGGCCGGGCGACCCAGATGTTTACTTAAAA    60
LT594108.1       ATGAGCCAGACCCAACCCCCGGCCCCAGTTGGGCCGGGCGACCCAGATGTTTACTTAAAA    60
MH160373.1       ATGAGCCAGACCCAACCCCCGGCCCCAGTTGGGCCGGGCGACCCAGATGTTTACTTAAAA    60
OP297866.1       ATGAGCCAGACCCAACCCCCGGCCCCAGTTGGGCCGGGCGACCCAGATGTTTACTTAAAA    60
LT594107.1       ATGAGCCAGACCCAACCCCCGGCCCCAGTTGGGCCGGGCGACCCAGATGTTTACTTAAAA    60
MG999856.1       ATGAGCCAGACCCAACCCCCGGCCCCAGTTGGGCCGGGCGACCCAGATGTTTACTTAAAA    60
MH160363.1       ATGAGCCAGACCCAACCCCCGGCCCCAGTTGGGCCGGGCGACCCAGATGTTTACTTAAAA    60
ON152715.1       ATGAGCCAGACCCAACCCCCGGCCCCAGTTGGGCCGGGCGACCCAGATGTTTACTTAAAA    60
MN401205.1       ATGAGCCAGACCCAACCCCCGGCCCCAGTTGGGCCGGGCGACCCAGATGTTTACTTAAAA    60
MN401207.1       ATGAGCCAGACCCAACCCCCGGCCCCAGTTGGGCCGGGCGACCCAGATGTTTACTTAAAA    60
MN401202.1       ATGAGCCAGACCCAACCCCCGGCCCCAGTTGGGCCGGGCGACCCAGATGTTTACTTAAAA    60
MG999858.1       ATGAGCCAGACCCAACCCCCGGCCCCAGTTGGGCCGGGCGACCCAGATGTTTACTTAAAA    60
KY922718.1       ATGAGCCAGACCCAACCCCCGGCCCCAGTTGGGCCGGGCGACCCAGATGTTTACTTAAAA    60
MG999891.1       ATGAGCCAGACCCAACCCCCGGCCCCAGTTGGGCCGGGCGACCCAGATGTTTACTTAAAA    60
KY922719.1       ATGAGCCAGACCCAACCCCCGGCCCCAGTTGGGCCGGGCGACCCAGATGTTTACTTAAAA    60
MN401203.1       ATGAGCCAGACCCAACCCCCGGCCCCAGTTGGGCCGGGCGACCCAGATGTTTACTTAAAA    60
MG999880.1       ATGAGCCAGACCCAACCCCCGGCCCCAGTTGGGCCGGGCGACCCAGATGTTTACTTAAAA    60
ON513441.1       ATGAGCCAGACCCAACCCCCGGCCCCAGTTGGGCCGGGCGACCCAGATGTTTACTTAAAA    60
MH999851.1       ATGAGCCAGACCCAACCCCCGGCCCCAGTTGGGCCGGGCGACCCAGATGTTTACTTAAAA    60
MN401204.1       ATGAGCCAGACCCAACCCCCGGCCCCAGTTGGGCCGGGCGACCCAGATGTTTACTTAAAA    60
MG999838.1       ATGAGCCAGACCCAACCCCCGGCCCCAGTTGGGCCGGGCGACCCAGATGTTTACTTAAAA    60
MH999842.1       ATGAGCCAGACCCAACCCCCGGCCCCAGTTGGGCCGGGCGACCCAGATGTTTACTTAAAA    60
ON960055.1       ATGAGCCAGACCCAACCCCCGGCCCCAGTTGGGCCGGGCGACCCAGATGTTTACTTAAAA    60
MN401208.1       ATGAGCCAGACCCAACCCCCGGCCCCAGTTGGGCCGGGCGACCCAGATGTTTACTTAAAA    60
MH999849.1       ATGAGCCAGACCCAACCCCCGGCCCCAGTTGGGCCGGGCGACCCAGATGTTTACTTAAAA    60
MH999845.1       ATGAGCCAGACCCAACCCCCGGCCCCAGTTGGGCCGGGCGACCCAGATGTTTACTTAAAA    60
OP297884.1       ATGAGCCAGACCCAACCCCCGGCCCCAGTTGGGCCGGGCGACCCAGATGTTTACTTAAAA    60
J02220.1         ATGAGCCAGACCCAACCCCCGGCCCCAGTTGGGCCGGGCGACCCAGATGTTTACTTAAAA    60
MK952183.1       ATGAGCCAGACCCAACCCCCGGCCCCAGTTGGGCCGGGCGACCCAGATGTTTCCTTAAAA    60
LT594105.1       ATGAGCCAGACCCAACCCCCGGCCCCAGTTGGGCCGGGCGACCCAGATGTTTACTTAAAA    60
MK952184.1       ATGAGCCAGACCCAACCCCCGGCCCCAGTTGGGCCGGGCGACCCAGATGTTTCCTTAAAA    60
KX424525.1       ATGAGCCAGACCCAACCCCCGGCCCCAGTTGGGCCGGGCGACCCAGATGTTTACTTAAAA    60
MK952185.1       ATGAGCCAGACCCAACCCCCGGCCCCAGTTGGGCCGGGCGACCCAGATGTTTCCTTAAAA    60
OP297862.1       ATGAGCCAGACCCAACCCCCGGCCCCAGTTGGGCCGGGCGACCCAGATGTTTACTTAAAA    60
MN401206.1       ATGAGCCAGACCCAACCCCCGGCCCCAGTTGGGCCGGGCGACCCAGATGTTTACTTAAAA    60
OP297872.1       ATGAGCCAGACCCAACCCCCGGCCCCAATTGGGCCGGGCGACCCAGATGTTTACTTAAAA    60
HM585502.2       ATGAGCCAGACCCAACCCCCGGCCCCAGTTGGGCCGGGCGACCCAGATGTTTACTTAAAA    60
KU310660.1       ATGAGCCAGACCCAACCCCCGGCCCCCGTTGGGCCGGGCGACCCAGATGTTTACTTAAAA    60
KU310661.1       ATGAGCCAGACCCAACCCCCGGCCCCCGTTGGGCCGGGCGACCCAGATGTTTACTTAAAA    60
DQ889502.1       ATGAGCCAGACCCAACCCCCGGCCCCCGTTGGGCCGGGCGACCCAGATGTTTACTTAAAA    60
AB618031.1       ATGAGCCAGACCCAACCCCCGGCCCCCGTTGGGCCGGGCGACCCAGATGTTTACTTAAAA    60
OP297885.1       ATGAGCCAGACCCAACCCCCGGCCCCCGTTGGGCCGGGCGACCCAGATGTTTACTTAAAA    60
ON960057.1       ATGAGCCAGACCCAACCCCCGGCCCCCGTTGGGCCGGGCGACCCAGATGTTTACTTAAAA    60
ON960061.1       ATGAGCCAGACCCAACCCCCGGCCCCCGTTGGGCCGGGCGACCCAGATGTTTACTTAAAA    60
LT594111.1       ATGAGCCAGACCCAACCCCCGGCCCCCGTTGGGCCGGGCGACCCAGATGTTTACTTAAAA    60
OP297886.1       ATGAGCCAGACCCAACCCCCGGCCCCCGTTGGGCCGGGCGACCCAGATGTTTACTTAAAA    60
MG999894.1       ATGAGCCAGACCCAACCCCCGGCCCCCGTTGGGCCGGGCGACCCAGATGTTTACTTAAAA    60
KM222726.1       ATGAGCCAGACCCAACCCCCGGCCCCCGTTGGGCCGGGCGACCCAGATGTTTACTTAAAA    60
ON960056.1       ATGAGCCAGACCCAACCCCCGGCCCCCGTTGGGCCGGGCGACCCAGATGTTTACTTAAAA    60
MG999851.1       ATGAGCCAGACCCAACCCCCGGCCCCCGTTGGGCCGGGCGACCCAGATGTTTACTTAAAA    60
OP297860.1       ATGAGCCAGACCCAACCCCCGGCCCCCGTTGGGCCGGGCGACCCAGATGTTTACTTAAAA    60
                 **************************  ************************ *******
```

```
NC_001806.2      GGCGTGCCGTCCGCCGGCATGCACCCCAGAGGTGTTCACGCACCTCGAGGACACCCGCGC    120
OP297876.1       GGCGTGCCGTCCGCCGGCATGCACCCCAGAGGTGTTCACGCACCTCGAGGACACCCGCGC    120
KT425108.1       GGCGTGCCGTCCGCCGGCATGCACCCCAGAGGTGTTCACGCACCTCGAGGACACCCGCGC    120
OP297880.1       GGCGTGCCGTCCGCCGGCATGCACCCCAGAGGTGTTCACGCACCTCGAGGACACCCGCGC    120
OP297875.1       GGCGTGCCGTCCGCCGGCATGCACCCCAGAGGTGTTCACGCACCTCGAGGACACCCGCGC    120
ON007163.1       GGCGTGCCGTCCGCCGGCATGCACCCCAGAGGTGTTCACGCACCTCGAGGACACCCGCGC    120
ON007155.1       GGCGTGCCGTCCGCCGGCATGCACCCCAGAGGTGTTCACGCACCTCGAGGACACCCGCGC    120
OP297877.1       GGCGTGCCGTCCGCCGGCATGCACCCCAGAGGTGTTCACGCACCTCGAGGACACCCGCGC    120
MN401201.1       GGCGTGCCGTCCGCCGGCATGCACCCCAGAGGTGTTCACGCACCTCGAGGACACCCGCGC    120
ON960054.1       GGCGTGCCGTCCGCCGGCATGCACCCCAGAGGTGTTCACGCACCTCGAGGACACCCGCGC    120
ON007144.1       GGCGTGCCGTCCGCCGGCATGCACCCCAGAGGTGTTCACGCACCTCGAGGACACCCGCGC    120
LT594457.1       GGCGTGCCGTCCGCCGGCATGCACCCCAGAGGTGTTCACGCACCTCGAGGACACCCGCGC    120
LT594122.1       GGCGTGCCGTCCGCCGGCATGCACCCCAGAGGTGTTCACGCACCTCGAGGACACCCGCGC    120
OP297863.1       GGCGTGCCGTCCGCCGGCATGCACCCCAGAGGTGTTCACGCACCTCGAGGACACCCGCGC    120
ON152716.1       GGCGTGCCGTCCGCCGGCATGCACCCCAGAGGTGTTCACGCACCTCGAGGACACCCGCGC    120
MH160374.1       GGCGTGCCGTCCGCCGGCATGCACCCCAGAGGTGTTCACGCACCTCGAGGACACCCGCGC    120
MH999846.1       GGCGTGCCGTCCGCCGGCATGCACCCCAGAGGTGTTCACGCACCTCGAGGACACCCGCGC    120
LT594108.1       GGCGTGCCGTCCGCCGGCATGCACCCCAGAGGTGTTCACGCACCTCGAGGACACCCGCGC    120
MH160373.1       GGCGTGCCGTCCGCCGGCATGCACCCCAGAGGTGTTCACGCACCTCGAGGACACCCGCGC    120
OP297866.1       GGCGTGCCGTCCGCCGGCATGCACCCCAGAGGTGTTCACGCACCTCGAGGACACCCGCGC    120
LT594107.1       GGCGTGCCGTCCGCCGGCATGCACCCCAGAGGTGTTCACGCACCTCGAGGACACCCGCGC    120
MG999856.1       GGCGTGCCGTCCGCCGGCATGCACCCCAGAGGTGTTCACGCACCTCGAGGACACCCGCGC    120
MH160363.1       GGCGTGCCGTCCGCCGGCATGCACCCCAGAGGTGTTCACGCACCTCGAGGACACCCGCGC    120
ON152715.1       GGCGTGCCGTCCGCCGGCATGCACCCCAGAGGTGTTCACGCACCTCGAGGACACCCGCGC    120
MN401205.1       GGCGTGCCGTCCGCCGGCATGCACCCCAGAGGTGTTCACGCACCTCGAGGACACCCGCGC    120
MN401207.1       GGCGTGCCGTCCGCCGGCATGCACCCCAGAGGTGTTCACGCACCTCGAGGACACCCGCGC    120
MN401202.1       GGCGTGCCGTCCGCCGGCATGCACCCCAGAGGTGTTCACGCACCTCGAGGACACCCGCGC    120
MG999858.1       GGCGTGCCGTCCGCCGGCATGCACCCCAGAGGTGTTCACGCACCTCGAGGACACCCGCGC    120
KY922718.1       GGCGTGCCGTCCGCCGGCATGCACCCCAGAGGTGTTCACGCACCTCGAGGACACCCGCGC    120
MG999891.1       GGCGTGCCGTCCGCCGGCATGCACCCCAGAGGTGTTCACGCACCTCGAGGACACCCGCGC    120
KY922719.1       GGCGTGCCGTCCGCCGGCATGCACCCCAGAGGTGTTCACGCACCTCGAGGACACCCGCGC    120
MN401203.1       GGCGTGCCGTCCGCCGGCATGCACCCCAGAGGTGTTCACGCACCTCGAGGACACCCGCGC    120
MG999880.1       GGCGTGCCGTCCGCCGGCATGCACCCCAGAGGTGTTCACGCACCTCGAGGACACCCGCGC    120
ON513441.1       GGCGTGCCGTCCGCCGGCATGCACCCCAGAGGTGTTCACGCACCTCGAGGACACCCGCGC    120
MH999851.1       GGCGTGCCGTCCGCCGGCATGCACCCCAGAGGTGTTCACGCACCTCGAGGACACCCGCGC    120
MN401204.1       GGCGTGCCGTCCGCCGGCATGCACCCCAGAGGTGTTCACGCACCTCGAGGACACCCGCGC    120
MG999838.1       GGCGTGCCGTCCGCCGGCATGCACCCCAGAGGTGTTCACGCACCTCGAGGACACCCGCGC    120
MH999842.1       GGCGTGCCGTCCGCCGGCATGCACCCCAGAGGTGTTCACGCACCTCGAGGACACCCGCGC    120
ON960055.1       GGCGTGCCGTCCGCCGGCATGCACCCCAGAGGTGTTCACGCACCTCGAGGACACCCGCGC    120
MN401208.1       GGCGTGCCGTCCGCCGGCATGCACCCCAGAGGTGTTCACGCACCTCGAGGACACCCGCGC    120
MH999849.1       GGCGTGCCGTCCGCCGGCATGCACCCCAGAGGTGTTCACGCACCTCGAGGACACCCGCGC    120
MH999845.1       GGCGTGCCGTCCGCCGGCATGCACCCCAGAGGTGTTCACGCACCTCGAGGACACCCGCGC    120
OP297884.1       GGCGTGCCGTCCGCCGGCATGCACCCCAGAGGTGTTCACGCACCTCGAGGACACCCGCGC    120
J02220.1         GGCGTGCCGTCCGCCGGCATGCACCCCAGAGGTGTTCACGCACCTCGAGGACACCCGCGC    120
MK952183.1       GGCGTGCCGTCCGCCGGCATGCACCCCAGAGGTGTTCACGCACCTCGAGGACACCCGCGC    120
LT594105.1       GGCGTGCCGTCCGCCGGCATGCACCCCAGAGGTGTTCACGCACCTCGAGGACACCCGCGC    120
MK952184.1       GGCGTGCCGTCCGCCGGCATGCACCCCAGAGGTGTTCACGCACCTCGAGGACACCCGCGC    120
KX424525.1       GGCGTGCCGTCCGCCGGCATGCACCCCAGAGGTGTTCACGCACCTCGAGGACACCCGCGC    120
MK952185.1       GGCGTGCCGTCCGCCGGCATGCACCCCAGAGGTGTTCACGCACCTCGAGGACACCCGCGC    120
OP297862.1       GGCGTGCCGTCCGCCGGCATGCACCCCAGAGGTGTTCACGCACCTCGAGGACACCCGCGC    120
MN401206.1       GGCGTGCCGTCCGCCGGCATGCACCCCAGAGGTGTTCACGCACCTCGAGGACACCCGCGC    120
OP297872.1       GGCGTGCCGTCCGCCGGCATGCACCCCAGAGGTGTTCGCGCACCTCGAGGACACCCGCGC    120
HM585502.2       GGCGTGCCGTCCGCCGGCATGCACCCCAGAGGTGTTCACGCACCTCGAGGACACCCGCGC    120
KU310660.1       GGCGTGCCGTCCGCCGGCATGCACCCCAGAGGTGTTCACGCACCTCGAGGACACCCGCGC    120
KU310661.1       GGCGTGCCGTCCGCCGGCATGCACCCCAGAGGTGTTCACGCACCTCGAGGACACCCGCGC    120
DQ889502.1       GGCATGCCGTCCGCCGGCATGCACCCCAGAGGTGTTCACGCACCTCGAGGACACCCGCGC    120
AB618031.1       GGCATGCCGTCCGCCGGCATGCACCCCAGAGGTGTTCACGCACCTCGAGGACACCCGCGC    120
OP297885.1       GGCATGCCGTCCGCCGGCATGCACCCCAGAGGTGTTCACGCACCTCGAGGACACCCGCGC    120
ON960057.1       GGCATGCCGTCCGCCGGCATGCACCCCAGAGGTGTTCACGCACCTCGAGGACACCCGCGC    120
ON960061.1       GGCGTGCCGTCCGCCGGCATGCACCCCAGAGGTGTTCACGCACCTCGAGGACACCCGCGC    120
LT594111.1       GGCATGCCGTCCGCCGGCATGCACCCCAGAGGTGTTCACGCACCTCGAGGACACCCGCGC    120
OP297886.1       GGCATGCCGTCCGCCGGCATGCACCCCAGAGGTGTTCACGCACCTCGAGGACACCCGCGC    120
MG999894.1       GGCATGCCGTCCGCCGGCATGCACCCCAGAGGTGTTCACGCACCTCGAGGACACCCGCGC    120
KM222726.1       GGCATGCCGTCCGCCGGCATGCACCCCAGAGGTGTTCACGCACCTCGAGGACACCCGCGC    120
ON960056.1       GGCATGCCGTCCGCCGGCATGCACCCCAGAGGTGTTCACGCACCTCGAGGACACCCGCGC    120
MG999851.1       GGCATGCCGTCCGCCGGCATGCACCCCAGAGGTGTTCACGCACCTCGAGGACACCCGCGC    120
OP297860.1       GGCATGCCGTCCGCCGGCATGCACCCCAGAGGTGTTCACGCACCTCGAGGACACCCGCGC    120
                 *** ********************************* **********************
```

```
NC_001806.2      ATGATCTCCGGACCCCCGCAACGGGGTGATAATGATCAAGCGGCGGGGCAATGTGGAGAT    180
OP297876.1       ATGATCTCCGGACCCCCGCAACGGGGTGATAATGATCAAGCGGCGGGGCAATGTGGAGAT    180
KT425108.1       ATGATCTCCGGACCCCCGCAACGGGGTGATAATGATCAAGCGGCGGGGCAATGTGGAGAT    180
OP297880.1       ATGATCTCCGGACCCCCGCAACGGGGTGATAATGATCAAGCGGCGGGGCAATGTGGAGAT    180
OP297875.1       ATGATCTCCGGACCCCCGCAACGGGGTGATAATGATCAAGCGGCGGGGCAATGTGGAGAT    180
ON007163.1       ATGATCTCCGGACCCCCGCAACGGGGTGATAATGATCAAGCGGCGGGGCAATGTGGAGAT    180
ON007155.1       ATGATCTCCGGACCCCCGCAACGGGGTGATAATGATCAAGCGGCGGGGCAATGTGGAGAT    180
OP297877.1       ATGATCTCCGGACCCCCGCAACGGGGTGATAATGATCAAGCGGCGGGGCAATGTGGAGAT    180
MN401201.1       ATGATCTCCGGACCCCCGCAACGGGGTGATAATGATCAAGCGGCGGGGCAATGTGGAGAT    180
ON960054.1       ATGATCTCCGGACCCCCGCAACGGGGTGATAATGATCAAGCGGCGGGGCAATGTGGAGAT    180
ON007144.1       ATGATCTCCGGACCCCCGCAACGGGGTGATAATGATCAAGCGGCGGGGCAATGTGGAGAT    180
LT594457.1       ATGATCTCCGGACCCCCGCAACGGGGTGATAATGATCAAGCGGCGGGGCAATGTGGAGAT    180
LT594122.1       ATGATCTCCGGACCCCCGCAACGGGGTGATAATGATCAAGCGGCGGGGCAATGTGGAGAT    180
OP297863.1       ATGATCTCCGGACCCCCGCAACGGGGTGATAATGATCAAGCGGCGGGGCAATGTGGAGAT    180
ON152716.1       ATGATCTCCGGACCCCCGCAACGGGGTGATAATGATCAAGCGGCGGGGCAATGTGGAGAT    180
MH160374.1       ATGATCTCCGGACCCCCGCAACGGGGTGATAATGATCAAGCGGCGGGGCAATGTGGAGAT    180
MH999846.1       ATGATCTCCGGACCCCCGCAACGGGGTGATAATGATCAAGCGGCGGGGCAATGTGGAGAT    180
LT594108.1       ATGATCTCCGGACCCCCGCAACGGGGTGATAATGATCAAGCGGCGGGGCAATGTGGAGAT    180
MH160373.1       ATGATCTCCGGACCCCCGCAACGGGGTGATAATGATCAAGCGGCGGGGCAATGTGGAGAT    180
OP297866.1       ATGATCTCCGGACCCCCGCAACGGGGTGATAATGATCAAGCGGCGGGGCAATGTGGAGAT    180
LT594107.1       ATGATCTCCGGACCCCCGCAACGGGGTGATAATGATCAAGCGGCGGGGCAATGTGGAGAT    180
MG999856.1       ATGATCTCCGGACCCCCGCAACGGGGTGATAATGATCAAGCGGCGGGGCAATGTGGAGAT    180
MH160363.1       ATGATCTCCGGACCCCCGCAACGGGGTGATAATGATCAAGCGGCGGGGCAATGTGGAGAT    180
ON152715.1       ATGATCTCCGGACCCCCGCAACGGGGTGATAATGATCAAGCGGCGGGGCAATGTGGAGAT    180
MN401205.1       ATGATCTCCGGACCCCCGCAACGGGGTGATAATGATCAAGCGGCGGGGCAATGTGGAGAT    180
MN401207.1       ATGATCTCCGGACCCCCGCAACGGGGTGATAATGATCAAGCGGCGGGGCAATGTGGAGAT    180
MN401202.1       ATGATCTCCGGACCCCCGCAACGGGGTGATAATGATCAAGCGGCGGGGCAATGTGGAGAT    180
MG999858.1       ATGATCTCCGGACCCCCGCAACGGGGTGATAATGATCAAGCGGCGGGGCAATGTGGAGAT    180
KY922718.1       ATGATCTCCGGACCCCCGCAACGGGGTGATAATGATCAAGCGGCGGGGCAATGTGGAGAT    180
MG999891.1       ATGATCTCCGGACCCCCGCAACGGGGTGATAATGATCAAGCGGCGGGGCAATGTGGAGAT    180
KY922719.1       ATGATCTCCGGACCCCCGCAACGGGGTGATAATGATCAAGCGGCGGGGCAATGTGGAGAT    180
MN401203.1       ATGATCTCCGGACCCCCGCAACGGGGTGATAATGATCAAGCGGCGGGGCAATGTGGAGAT    180
MG999880.1       ATGATCTCCGGACCCCCGCAACGGGGTGATAATGATCAAGCGGCGGGGCAATGTGGAGAT    180
ON513441.1       ATGATCTCCGGACCCCCGCAACGGGGTGATAATGATCAAGCGGCGGGGCAATGTGGAGAT    180
MH999851.1       ATGATCTCCGGACCCCCGCAACGGGGTGATAATGATCAAGCGGCGGGGCAATGTGGAGAT    180
MN401204.1       ATGATCTCCGGACCCCCGCAACGGGGTGATAATGATCAAGCGGCGGGGCAATGTGGAGAT    180
MG999838.1       ATGATCTCCGGACCCCCGCAACGGGGTGATAATGATCAAGCGGCGGGGCAATGTGGAGAT    180
MH999842.1       ATGATCTCCGGACCCCCGCAACGGGGTGATAATGATCAAGCGGCGGGGCAATGTGGAGAT    180
ON960055.1       ATGATCTCCGGACCCCCGCAACGGGGTGATAATGATCAAGCGGCGGGGCAATGTGGAGAT    180
MN401208.1       ATGATCTCCGGACCCCCGCAACGGGGTGATAATGATCAAGCGGCGGGGCAATGTGGAGAT    180
MH999849.1       ATGATCTCCGGACCCCCGCAACGGGGTGATAATGATCAAGCGGCGGGGCAATGTGGAGAT    180
MH999845.1       ATGATCTCCGGACCCCCGCAACGGGGTGATAATGATCAAGCGGCGGGGCAATGTGGAGAT    180
OP297884.1       ATGATCTCCGGACCCCCGCAACGGGGTGATAATGATCAAGCGGCGGGGCAATGTGGAGAT    180
J02220.1         ATGATCTCCGGACCCCCGCAACGGGGTGATAATGATCAAGCGGCGGGGCAATGTGGAGAT    180
MK952183.1       ATGATCTCCGGACCCCCGCAACGGGGTGATAATGATCAAGCGGCGGGGCAATGTGGAGAT    180
LT594105.1       ATGATCTCCGGACCCCCGCAACGGGGTGATAATGATCAAGCGGCGGGGCAATGTGGAGAT    180
MK952184.1       ATGATCTCCGGACCCCCGCAACGGGGTGATAATGATCAAGCGGCGGGGCAATGTGGAGAT    180
KX424525.1       ATGATCTCCGGACCCCCGCAACGGGGTGATAATGATCAAGCGGCGGGGCAATGTGGAGAT    180
MK952185.1       ATGATCTCCGGACCCCCGCAACGGGGTGATAATGATCAAGCGGCGGGGCAATGTGGAGAT    180
OP297862.1       ATGATCTCCGGACCCCCGCAACGGGGTGATAATGATCAAGCGGCGGGGCAATGTGGAGAT    180
MN401206.1       ATGATCTCCGGACCCCCGCAACGGGGTGATAATGATCAAGCGGCGGGGCAATGTGGAGAT    180
OP297872.1       ATGATCTCCGGACCCCCGCAACGGGGTGATAATGATCAAGCGGCGGGGCAATGTGGAGAT    180
HM585502.2       ATGATCTCCGGACCCCCGCAACGGGGTGATAATGATCAAGCGGCGGGGCAATGTGGAGAT    180
KU310660.1       ATGATCTCCGGACCCCCGCAACGGGGTGATAATGATCAAGCGGCGGGGCAATGTGGAGAT    180
KU310661.1       ATGATCTCCGGACCCCCGCAACGGGGTGATAATGATCAAGCGGCGGGGCAATGTGGAGAT    180
DQ889502.1       ATGATCTCCGGACCCCCACAACGGGGTGATAATGATCAAGCGGCGGGGCAATGTGGAGAT    180
AB618031.1       ATGATCTCCGGACCCCCACAACGGGGTGATAATGATCAAGCGGCGGGGCAATGTGGAGAT    180
OP297885.1       ATGATCTCCGGACCCCCACAACGGGGTGATAATGATCAAGCGGCGGGGCAATGTGGAGAT    180
ON960057.1       ATGATCTCCGGACCCCCACAACGGGGTGATAATGATCAAGCGGCGGGGCAATGTGGAGAT    180
ON960061.1       ATGATCTCCGGACCCCCGCAACGGGGTGATAATGATCAAGCGGCGGGGCAATGTGGAGAT    180
LT594111.1       ATGATCTCCGGACCCCCACAACGGGGTGATAATGATCAAGCGGCGGGGCAATGTGGAGAT    180
OP297886.1       ATGATCTCCGGACCCCCACAACGGGGTGATAATGATCAAGCGGCGGGGCAATGTGGAGAT    180
MG999894.1       ATGATCTCCGGACCCCCACAACGGGGTGATAATGATCAAGCGGCGGGGCAATGTGGAGAT    180
KM222726.1       ATGATCTCCGGACCCCCACAACGGGGTGATAATGATCAAGCGGCGGGGCAATGTGGAGAT    180
ON960056.1       ATGATCTCCGGACCCCCACAACGGGGTGATAATGATCAAGCGGCGGGGCAATGTGGAGAT    180
MG999851.1       ATGATCTCCGGACCCCCACAACGGGGTGATAATGATCAAGCGGCGGGGCAATGTGGAGAT    180
OP297860.1       ATGATCTCCGGACCCCCACAACGGGGTGATAATGATCAAGCGGCGGGGCAATGTGGAGAT    180
                 ***************** ******************************************
```

```
NC_001806.2       TCGGGTCTACTACGAGTCGGTGCGGACACTACGATCTCGAAGCCATCTGAAGCCGTCCGA    240
OP297876.1        TCGGGTCTACTACGAGTCGGTGCGGACACTACGATCTCGAAGCCATCTGAAGCCGTCCGA    240
KT425108.1        TCGGGTCTACTACGAGTCGGTGCGGACACTACGATCTCGAAGCCATCTGAAGCCGTCCGA    240
OP297880.1        TCGGGTCTACTACGAGTCGGTGCGGACACTACGATCTCGAAGCCATCTGAAGCCGTCCGA    240
OP297875.1        TCGGGTCTACTACGAGTCGGTGCGGACACTACGATCTCGAAGCCATCTGAAGCCGTCCGA    240
ON007163.1        TCGGGTCTACTACGAGTCGGTGCGGACACTACGATCTCGAAGCCATCTGAAGCCGTCCGA    240
ON007155.1        TCGGGTCTACTACGAGTCGGTGCGGACACTACGATCTCGAAGCCATCTGAAGCCGTCCGA    240
OP297877.1        TCGGGTCTACTACGAGTCGGTGCGGACACTACGATCTCGAAGCCATCTGAAGCCGTCCGA    240
MN401201.1        TCGGGTCTACTACGAGTCGGTGCGGACACTACGATCTCGAAGCCATCTGAAGCCGTCCGA    240
ON960054.1        TCGGGTCTACTACGAGTCGGTGCGGACACTACGATCTCGAAGCCATCTGAAGCCGTCCGA    240
ON007144.1        TCGGGTCTACTACGAGTCGGTGCGGACACTACGATCTCGAAGCCATCTGAAGCCGTCCGA    240
LT594457.1        TCGGGTCTACTACGAGTCGGTGCGGACACTACGATCTCGAAGCCATCTGAAGCCGTCCGA    240
LT594122.1        TCGGGTCTACTACGAGTCGGTGCGGACACTACGATCTCGAAGCCATCTGAAGCCGTCCGA    240
OP297863.1        TCGGGTCTACTACGAGTCGGTGCGGACACTACGATCTCGAAGCCATCTGAAGCCGTCCGA    240
ON152716.1        TCGGGTCTACTACGAGTCGGTGCGGACACTACGATCTCGAAGCCATCTGAAGCCGTCCGA    240
MH160374.1        TCGGGTCTACTACGAGTCGGTGCGGACACTACGATCTCGAAGCCATCTGAAGCCGTCCGA    240
MH999846.1        TCGGGTCTACTACGAGTCGGTGCGGACACTACGATCTCGAAGCCATCTGAAGCCGTCCGA    240
LT594108.1        TCGGGTCTACTACGAGTCGGTGCGGACACTACGATCTCGAAGCCATCTGAAGCCGTCCGA    240
MH160373.1        TCGGGTCTACTACGAGTCGGTGCGGACACTACGATCTCGAAGCCATCTGAAGCCGTCCGA    240
OP297866.1        TCGGGTCTACTACGAGTCGGTGCGGACACTACGATCTCGAAGCCATCTGAAGCCGTCCGA    240
LT594107.1        TCGGGTCTACTACGAGTCGGTGCGGACACTACGATCTCGAAGCCATCTGAAGCCGTCCGA    240
MG999856.1        TCGGGTCTACTACGAGTCGGTGCGGACACTACGATCTCGAAGCCATCTGAAGCCGTCCGA    240
MH160363.1        TCGGGTCTACTACGAGTCGGTGCGGACACTACGATCTCGAAGCCATCTGAAGCCGTCCGA    240
ON152715.1        TCGGGTCTACTACGAGTCGGTGCGGACACTACGATCTCGAAGCCATCTGAAGCCGTCCGA    240
MN401205.1        TCGGGTCTACTACGAGTCGGTGCGGACACTACGATCTCGAAGCCATCTGAAGCCGTCCGA    240
MN401207.1        TCGGGTCTACTACGAGTCGGTGCGGACACTACGATCTCGAAGCCATCTGAAGCCGTCCGA    240
MN401202.1        TCGGGTCTACTACGAGTCGGTGCGGACACTACGATCTCGAAGCCATCTGAAGCCGTCCGA    240
MG999858.1        TCGGGTCTACTACGAGTCGGTGCGGACACTACGATCTCGAAGCCATCTGAAGCCGTCCGA    240
KY922718.1        TCGGGTCTACTACGAGTCGGTGCGGACACTACGATCTCGAAGCCATCTGAAGCCGTCCGA    240
MG999891.1        TCGGGTCTACTACGAGTCGGTGCGGACACTACGATCTCGAAGCCATCTGAAGCCGTCCGA    240
KY922719.1        TCGGGTCTACTACGAGTCGGTGCGGACACTACGATCTCGAAGCCATCTGAAGCCGTCCGA    240
MN401203.1        TCGGGTCTACTACGAGTCGGTGCGGACACTACGATCTCGAAGCCATCTGAAGCCGTCCGA    240
MG999880.1        TCGGGTCTACTACGAGTCGGTGCGGACACTACGATCTCGAAGCCATCTGAAGCCGTCCGA    240
ON513441.1        TCGGGTCTACTACGAGTCGGTGCGGACACTACGATCTCGAAGCCATCTGAAGCCGTCCGA    240
MH999851.1        TCGGGTCTACTACGAGTCGGTGCGGACACTACGATCTCGAAGCCATCTGAAGCCGTCCGA    240
MN401204.1        TCGGGTCTACTACGAGTCGGTGCGGACACTACGATCTCGAAGCCATCTGAAGCCGTCCGA    240
MG999838.1        TCGGGTCTACTACGAGTCGGTGCGGACACTACGATCTCGAAGCCATCTGAAGCCGTCCGA    240
MH999842.1        TCGGGTCTACTACGAGTCGGTGCGGACACTACGATCTCGAAGCCATCTGAAGCCGTCCGA    240
ON960055.1        TCGGGTCTACTACGAGTCGGTGCGGACACTACGATCTCGAAGCCATCTGAAGCCGTCCGA    240
MN401208.1        TCGGGTCTACTACGAGTCGGTGCGGACACTACGATCTCGAAGCCATCTGAAGCCGTCCGA    240
MH999849.1        TCGGGTCTACTACGAGTCGGTGCGGACACTACGATCTCGAAGCCATCTGAAGCCGTCCGA    240
MH999845.1        TCGGGTCTACTACGAGTCGGTGCGGACACTACGATCTCGAAGCCATCTGAAGCCGTCCGA    240
OP297884.1        TCGGGTCTACTACGAGTCGGTGCGGACACTACGATCTCGAAGCCATCTGAAGCCGTCCGA    240
J02220.1          TCGGGTCTACTACGAGTCGGTGCGGACACTACGATCTCGAAGCCATCTGAAGCCGTCCGA    240
MK952183.1        TCGGGTCTACTACGAGTCGGTGCGGACACTACGATCTCGAAGCCATCTGAAGCCGTCCGA    240
LT594105.1        TCGGGTCTACTACGAGTCGGTGCGGACACTACGATCTCGAAGCCATCTGAAGCCGTCCGA    240
MK952184.1        TCGGGTCTACTACGAGTCGGTGCGGACACTACGATCTCGAAGCCATCTGAAGCCGTCCGA    240
KX424525.1        TCGGGTCTACTACGAGTCGGTGCGGACACTACGATCTCGAAGCCATCTGAAGCCGTCCGA    240
MK952185.1        TCGGGTCTACTACGAGTCGGTGCGGACACTACGATCTCGAAGCCATCTGAAGCCGTCCGA    240
OP297862.1        TCGGGTCTACTACGAGTCGGTGCGGACACTACGATCTCGAAGCCATCTGAAGCCGTCCAA    240
MN401206.1        TCGGGTCTACTACGAGTCGGTGCGGACACTACGATCTCGAAGCCATCTGAAGCCGTCCGA    240
OP297872.1        TCGGGTCTACTACGAGTCGGTGCGGACACTACGATCTCGAAGCCATCTGAAGCCGTCCGA    240
HM585502.2        TCGGGTCTACTACGAGTCGGTGCGGACACTACGATCTCGAAGCCATCTGAAGCCGTCCGA    240
KU310660.1        TCGGGTCTACTACGAGTCGGTGCGGACACTACGATCTCGAAGCCATCTGAAGCCGTCCGA    240
KU310661.1        TCGGGTCTACTACGAGTCGGTGCGGACACTACGATCTCGAAGCCATCTGAAGCCGTCCGA    240
DQ889502.1        TCGGGTCTACTACGAGTCGGTGCGGACACTACGATCTCGAAGCCATCTGAAGCCGTCCGA    240
AB618031.1        TCGGGTCTACTACGAGTCGGTGCGGACACTACGATCTCGAAGCCATCTGAAGCCGTCCGA    240
OP297885.1        TCGGGTCTACTACGAGTCGGTGCGGACACTACGATCTCGAAGCCATCTGAAGCCGTCCGA    240
ON960057.1        TCGGGTCTACTACGAGTCGGTGCGGACACTACGATCTCGAAGCCATCTGAAGCCGTCCGA    240
ON960061.1        TCGGGTCTACTACGAGTCGGTGCGGACACTACGATCTCGAAGCCATCTGAAGCCGTCCGA    240
LT594111.1        TCGGGTCTACTACGAGTCGGTGCGGACACTACGATCTCGAAGCCATCTGAAGCCGTCCGA    240
OP297886.1        TCGGGTCTACTACGAGTCGGTGCGGACACTACGATCTCGAAGCCATCTGAAGCCGTCCGA    240
MG999894.1        TCGGGTCTACTACGAGTCGGTGCGGACACTACGATCTCGAAGCCATCTGAAGCCGTCCGA    240
KM222726.1        TCGGGTCTACTACGAGTCGGTGCGGACACTACGATCTCGAAGCCATCTGAAGCCGTCCGA    240
ON960056.1        TCGGGTCTACTACGAGTCGGTGCGGACACTACGATCTCGAAGCCATCTGAAGCCGTCCGA    240
MG999851.1        TCGGGTCTACTACGAGTCGGTGCGGACACTACGATCTCGAAGCCATCTGAAGCCGTCCGA    240
OP297860.1        TCGGGTCTACTACGAGTCGGTGCGGACACTACGATCTCGAAGCCATCTGAAGCCGTCCGA    240
                  ********************************************************** *
```

```
NC_001806.2       CCGCCAACAATCCCCAGGACACCGCGTGTTCCCCGGGAGCCCCGGGTTCCGCGACCACCC    300
OP297876.1        CCGCCAACAATCCCCAGGACACCGCGTGTTCCCCGGGAGCCCCGGGTTCCGCGACCACCC    300
KT425108.1        CCGCCAACAATCCCCAGGACACCGCGTGTTCCCCGGGAGCCCCGGGTTCCGCGACCACCC    300
OP297880.1        CCGCCAACAATCCCCAGGACACCGCGTGTTCCCCGGGAGCCCCGGGTTCCGCGACCACCC    300
OP297875.1        CCGCCAACAATCCCCAGGACACCGCGTGTTCCCCGGGAGCCCCGGGTTCCGCGACCACCC    300
ON007163.1        CCGCCAACAATCCCCAGGACACCGCGTGTTCCCCGGGAGCCCCGGGTTCCGCGACCACCC    300
ON007155.1        CCGCCAACAATCCCCAGGACACCGCGTGTTCCCCGGGAGCCCCGGGTTCCGCGACCACCC    300
OP297877.1        CCGCCAACAATCCCCAGGACACCGCGTGTTCCCCGGGAGCCCCGGGTTCCGCGACCACCC    300
MN401201.1        CCGCCAACAATCCCCAGGACACCGCGTGTTCCCCGGGAGCCCCGGGTTCCGCGACCACCC    300
ON960054.1        CCGCCAACAATCCCCAGGACACCGCGTGTTCCCCGGGAGCCCCGGGTTCCGCGACCACCC    300
ON007144.1        CCGCCAACAATCCCCAGGACACCGCGTGTTCCCCGGGAGCCCCGGGTTCCGCGACCACCC    300
LT594457.1        CCGCCAACAATCCCCAGGACACCGCGTGTTCCCCGGGAGCCCCGGGTTCCGCGACCACCC    300
LT594122.1        CCGCCAACAATCCCCAGGACACCGCGTGTTCCCCGGGAGCCCCGGGTTCCGCGACCACCC    300
OP297863.1        CCGCCAACAATCCCCAGGACACCGCGTGTTCCCCGGGAGCCCCGGGTTCCGCGACCACCC    300
ON152716.1        CCGCCAACAATCCCCAGGACACCGCGTGTTCCCCGGGAGCCCCGGGTTCCGCGACCACCC    300
MH160374.1        CCGCCAACAATCCCCAGGACACCGCGTGTTCCCCGGGAGCCCCGGGTTCCGCGACCACCC    300
MH999846.1        CCGCCAACAATCCCCAGGACACCGCGTGTTCCCCGGGAGCCCCGGGTTCCGCGACCACCC    300
LT594108.1        CCGCCAACAATCCCCAGGACACCGCGTGTTCCCCGGGAGCCCCGGGTTCCGCGACCACCC    300
MH160373.1        CCGCCAACAATCCCCAGGACACCGCGTGTTCCCCGGGAGCCCCGGGTTCCGCGACCACCC    300
OP297866.1        CCGCCAACAATCCCCAGGACACCGCGTGTTCCCCGGGAGCCCCGGGTTCCGCGACCACCC    300
LT594107.1        CCGCCAACAATCCCCAGGACACCGCGTGTTCCCCGGGAGCCCCGGGTTCCGCGACCACCC    300
MG999856.1        CCGCCAACAATCCCCAGGACACCGCGTGTTCCCCGGGAGCCCCGGGTTCCGCGACCACCC    300
MH160363.1        CCGCCAACAATCCCCAGGACACCGCGTGTTCCCCGGGAGCCCCGGGTTCCGCGACCACCC    300
ON152715.1        CCGCCAACAATCCCCAGGACACCGCGTGTTCCCCGGGAGCCCCGGGTTCCGCGACCACCC    300
MN401205.1        CCGCCAACAATCCCCAGGACACCGCGTGTTCCCCGGGAGCCCCGGGTTCCGCGACCACCC    300
MN401207.1        CCGCCAACAATCCCCAGGACACCGCGTGTTCCCCGGGAGCCCCGGGTTCCGCGACCACCC    300
MN401202.1        CCGCCAACAATCCCCAGGACACCGCGTGTTCCCCGGGAGCCCCGGGTTCCGCGACCACCC    300
MG999858.1        CCGCCAACAATCCCCAGGACACCGCGTGTTCCCCGGGAGCCCCGGGTTCCGCGACCACCC    300
KY922718.1        CCGCCAACAATCCCCAGGACACCGCGTGTTCCCCGGGAGCCCCGGGTTCCGCGACCACCC    300
MG999891.1        CCGCCAACAATCCCCAGGACACCGCGTGTTCCCCGGGAGCCCCGGGTTCCGCGACCACCC    300
KY922719.1        CCGCCAACAATCCCCAGGACACCGCGTGTTCCCCGGGAGCCCCGGGTTCCGCGACCACCC    300
MN401203.1        CCGCCAACAATCCCCAGGACACCGCGTGTTCCCCGGGAGCCCCGGGTTCCGCGACCACCC    300
MG999880.1        CCGCCAACAATCCCCAGGACACCGCGTGTTCCCCGGGAGCCCCGGGTTCCGCGACCACCC    300
ON513441.1        CCGCCAACAATCCCCAGGACACCGCGTGTTCCCCGGGAGCCCCGGGTTCCGCGACCACCC    300
MH999851.1        CCGCCAACAATCCCCAGGACACCGCGTGTTCCCCGGGAGCCCCGGGTTCCGCGACCACCC    300
MN401204.1        CCGCCAACAATCCCCAGGACACCGCGTGTTCCCCGGGAGCCCCGGGTTCCGCGACCACCC    300
MG999838.1        CCGCCAACAATCCCCAGGACACCGCGTGTTCCCCGGGAGCCCCGGGTTCCGCGACCACCC    300
MH999842.1        CCGCCAACAATCCCCAGGACACCGCGTGTTCCCCGGGAGCCCCGGGTTCCGCGACCACCC    300
ON960055.1        CCGCCAACAATCCCCAGGACACCGCGTGTTCCCCGGGAGCCCCGGGTTCCGCGACCACCC    300
MN401208.1        CCGCCAACAATCCCCAGGACACCGCGTGTTCCCCGGGAGCCCCGGGTTCCGCGACCACCC    300
MH999849.1        CCGCCAACAATCCCCAGGACACCGCGTGTTCCCCGGGAGCCCCGGGTTCCGCGACCACCC    300
MH999845.1        CCGCCAACAATCCCCAGGACACCGCGTGTTCCCCGGGAGCCCCGGGTTCCGCGACCACCC    300
OP297884.1        CCGCCAACAATCCCCAGGACACCGCGTGTTCCCCGGGAGCCCCGGGTTCCGCGACCACCC    300
J02220.1          CCGCCAACAATCCCCAGGACACCGCGTGTTCCCCGGGAGCCCCGGGTTCCGCGACCACCC    300
MK952183.1        CCGCCAACAATCCCCAGGACACCGCGTGTTCCCCGGGAGCCCCGGGTTCCGCGACCACCC    300
LT594105.1        CCGCCAACAATCCCCAGGACACCGCGTGTTCCCCGGGAGCCCCGGGTTCCGCGCCCACCC    300
MK952184.1        CCGCCAACAATCCCCAGGACACCGCGTGTTCCCCGGGAGCCCCGGGTTCCGCGACCACCC    300
KX424525.1        CCGCCAACAATCCCCAGGACACCGCGTGTTCCCCGGGAGCCCCGGGTTCCGCGACCACCC    300
MK952185.1        CCGCCAACAATCCCCAGGACACCGCGTGTTCCCCGGGAGCCCCGGGTTCCGCGACCACCC    300
OP297862.1        CCGCCAACAATCCCCAGGACACCGCGTGTTCCCCGGGAGCCCCGGGTTCCGCGACCACCC    300
MN401206.1        CCGCCAACAATCCCCAGGACACCGCGTGTTCCCCGGGAGCCCCGGGTTCCGCGACCACCC    300
OP297872.1        CCGCCAACAATCCCCCGGACACCGCGTGTTCCCCGGGAGCCCCGGGTTCCGCGACCACCC    300
HM585502.2        CCGCCAACATTCCCCAGGACGCCGCGTGTTCCCCGGGAGCCCCGGGTTCCGCGACCACCC    300
KU310660.1        CCACCAACAATCCCCAGGACACCGCGTGTTCCCCGGGAGCCCCGGGTTCCGCGACCACCC    300
KU310661.1        CCACCAACAATCCCCAGGACACCGCGTGTTCCCCGGGAGCCCCGGGTTCCGCGACCACCC    300
DQ889502.1        CCACCAACAATCCCCAGGACACCGCGTGTTCCCCGGGAGCCCCGGGTTCCGCGACCACCC    300
AB618031.1        CCACCAACAATCCCCAGGACACCGCGTGTTCCCCGGGAGCCCCGGGTTCCGCGACCACCC    300
OP297885.1        CCACCAACAATCCCCAGGACACCGCGTGTTCCCCGGGAGCCCCGGGTTCCGCGACCACCC    300
ON960057.1        CCACCAACAATCCCCAGGACACCGCGTGTTCCCCGGGAGCCCCGGGTTCCGCGACCACCC    300
ON960061.1        CCACCAACAATCCCCAGGACACCGCGTGTTCCCCGGGAGCCCCGGGTTCCGCGACCACCC    300
LT594111.1        CCACCAACAATCCCCAGGACACCGCGTGTTCCCCGGGAGCCCCGGGTTCCGCGACCACCC    300
OP297886.1        CCACCAACAATCCCCAGGACACCGCGTGTTCCCCGGGAGCCCCGGGTTCCGCGACCACCC    300
MG999894.1        CCACCAACAATCCCCAGGACACCGCGTGTTCCCCGGGAGCCCCGGGTTCCGCGACCACCC    300
KM222726.1        CCACCAACAATCCCCAGGACACCGCGTGTTCCCCGGGAGCCCCGGGTTCCGCGACCACCC    300
ON960056.1        CCACCAACAATCCCCAGGACACCGCGTGTTCCCCGGGAGCCCCGGGTTCCGCGACCACCC    300
MG999851.1        CCACCAACAATCCCCAGGACACCGCGTGTTCCCCGGGAGCCCCGGGTTCCGCGACCACCC    300
OP297860.1        CCACCAACAATCCCCAGGACACCGCGTGTTCCCCGGGAGCCCCGGGTTCCGCGACCACCC    300
                  ** ****** ***** **** ******************************** ******
```

```
NC_001806.2        CGAGAACCTAGGGAACCCAGAGTACCGCGAGCTCCCAGAGACCCCAGGGTACCGCGTGAC     360
OP297876.1         CGAGAACCTAGGGAACCCAGAGTACCGCGAGCTCCCAGAGACCCCAGGGTACCGCGTGAC     360
KT425108.1         CGAGAACCTAGGGAACCCAGAGTACCGCGAGCTCCCAGAGACCCCAGGGTACCGCGTGAC     360
OP297880.1         CGAGAACCTAGGGAACCCAGAGTACCGCGAGCTCCCAGAGACCCCAGGGTACCGCGTGAC     360
OP297875.1         CGAGAACCTAGGGAACCCAGAGTACCGCGAGCTCCCAGAGACCCCAGGGTACCGCGTGAC     360
ON007163.1         CGAGAACCTAGGGAACCCAGAGTACCGCGAGCTCCCAGAGACCCCAGGGTACCGCGTGAC     360
ON007155.1         CGAGAACCTAGGGAACCCAGAGTACCGCGAGCTCCCAGAGACCCCAGGGTACCGCGTGAC     360
OP297877.1         CGAGAACCTAGGGAACCCAGAGTACCGCGAGCTCCCAGAGACCCCAGGGTACCGCGTGAC     360
MN401201.1         CGAGAACCTAGGGAACCCAGAGTACCGCGAGCTCCCAGAGACCCCAGGGTACCGCGTGAC     360
ON960054.1         CGAGAACCTAGGGAACCCAGAGTACCGCGAGCTCCCAGAGACCCCAGGGTACCGCGTGAC     360
ON007144.1         CGAGAACCTAGGGAACCCAGAGTACCGCGAGCTCCCAGAGACCCCAGGGTACCGCGTGAC     360
LT594457.1         CGAGAACCTAGGGAACCCAGAGTACCGCGAGCTCCCAGAGACCCCAGGGTACCGCGTGAC     360
LT594122.1         CGAGAACCTAGGGAACCCAGAGTACCGCGAGCTCCCAGAGACCCCAGGGTACCGCGTGAC     360
OP297863.1         CGAGAACCTAGGGAACCCAGAGTACCGCGAGCTCCCAGAGACCCCAGGGTACCGCGTGAC     360
ON152716.1         CGAGAACCTAGGGAACCCAGAGTACCGCGAGCTCCCAGAGACCCCAGGGTACCGCGTGAC     360
MH160374.1         CGAGAACCTAGGGAACCCAGAGTACCGCGAGCTCCCAGAGACCCCAGGGTACCGCGTGAC     360
MH999846.1         CGAGAACCTAGGGAACCCAGAGTACCGCGAGCTCCCAGAGACCCCAGGGTACCGCGTGAC     360
LT594108.1         CGAGAACCTAGGGAACCCAGAGTACCGCGAGCTCCCAGAGACCCCAGGGTACCGCGTGAC     360
MH160373.1         CGAGAACCTAGGGAACCCAGAGTACCGCGAGCTCCCAGAGACCCCAGGGTACCGCGTGAC     360
OP297866.1         CGAGAACCTAGGGAACCCAGAGTACCGCGAGCTCCCAGAGACCCCAGGGTACCGCGTGAC     360
LT594107.1         CGAGAACCTAGGGAACCCAGAGTACCGCGAGCTCCCAGAGACCCCAGGGTACCGCGTGAC     360
MG999856.1         CGAGAACCTAGGGAACCCAGAGTACCGCGAGCTCCCAGAGACCCCAGGGTACCGCGTGAC     360
MH160363.1         CGAGAACCTAGGGAACCCAGAGTACCGCGAGCTCCCAGAGACCCCAGGGTACCGCGTGAC     360
ON152715.1         CGAGAACCTAGGGAACCCAGAGTACCGCGAGCTCCCAGAGACCCCAGGGTACCGCGTGAC     360
MN401205.1         CGAGAACCTAGGGAACCCAGAGTACCGCGAGCTCCCAGAGACCCCAGGGTACCGCGTGAC     360
MN401207.1         CGAGAACCTAGGGAACCCAGAGTACCGCGAGCTCCCAGAGACCCCAGGGTACCGCGTGAC     360
MN401202.1         CGAGAACCTAGGGAACCCAGAGTACCGCGAGCTCCCAGAGACCCCAGGGTACCGCGTGAC     360
MG999858.1         CGAGAACCTAGGGAACCCAGAGTACCGCGAGCTCCCAGAGACCCCAGGGTACCGCGTGAC     360
KY922718.1         CGAGAACCTAGGGAACCCAGAGTACCGCGAGCTCCCAGAGACCCCAGGGTACCGCGTGAC     360
MG999891.1         CGAGAACCTAGGGAACCCAGAGTACCGCGAGCTCCCAGAGACCCCAGGGTACCGCGTGAC     360
KY922719.1         CGAGAACCTAGGGAACCCAGAGTACCGCGAGCTCCCAGAGACCCCAGGGTACCGCGTGAC     360
MN401203.1         CGAGAACCTAGGGAACCCAGAGTACCGCGAGCTCCCAGAGACCCCAGGGTACCGCGTGAC     360
MG999880.1         CGAGAACCTAGGGAACCCAGAGTACCGCGAGCTCCCAGAGACCCCAGGGTACCGCGTGAC     360
ON513441.1         CGAGAACCTAGGGAACCCAGAGTACCGCGAGCTCCCAGAGACCCCAGGGTACCGCGTGAC     360
MH999851.1         CGAGAACCTAGGGAACCCAGAGTACCGCGAGCTCCCAGAGACCCCAGGGTACCGCGTGAC     360
MN401204.1         CGAGAACCTAGGGAACCCAGAGTACCGCGAGCTCCCAGAGACCCCAGGGTACCGCGTGAC     360
MG999838.1         CGAGAACCTAGGGAACCCAGAGTACCGCGAGCTCCCAGAGACCCCAGGGTACCGCGTGAC     360
MH999842.1         CGAGAACCTAGGGAACCCAGAGTACCGCGAGCTCCCAGAGACCCCAGGGTACCGCGTGAC     360
ON960055.1         CGAGAACCTAGGGAACCCAGAGTACCGCGAGCTCCCAGAGACCCCAGGGTACCGCGTGAC     360
MN401208.1         CGAGAACCTAGGGAACCCAGAGTACCGCGAGCTCCCAGAGACCCCAGGGTACCGCGTGAC     360
MH999849.1         CGAGAACCTAGGGAACCCAGAGTACCGCGAGCTCCCAGAGACCCCAGGGTACCGCGTGAC     360
MH999845.1         CGAGAACCTAGGGAACCCAGAGTACCGCGAGCTCCCAGAGACCCCAGGGTACCGCGTGAC     360
OP297884.1         CGAGAACCTAGGGAACCCAGAGTACCGCGAGCTCCCAGAGACCCCAGGGTACCGCGTGAC     360
J02220.1           CGAGAACCTAGGGAACCCAGAGTACCGCGAGATCCCAGAGACCCCAGGGTACCGCGTGAC     360
MK952183.1         CGAGAACCTAGGGAACCCAGAGTACCGCGAGCTCCCAGAGACCCCAGGGTACCGCGTGAC     360
LT594105.1         CGAGAACCTAGGGAACCCAGAGTACCGCGAGCTCCCAGAGACCCCAGGGTACCGCGTGAC     360
MK952184.1         CGAGAACCTAGGGAACCCAGAGTACCGCGAGCTCCCAGAGACCCCAGGGTACCGCGTGAC     360
KX424525.1         CGAGAACCTAGGGAACCCAGAGTACCGCGAGCTCCCAGAGACCCCAGGGTACCGCGTGAC     360
MK952185.1         CGAGAACCTAGGGAACCCAGAGTACCGCGAGCTCCCAGAGACCCCAGGGTACCGCGTGAC     360
OP297862.1         CGAGAACCTAGGGAACCCAGAGTACCGCGAGCTCCCAGAGACCCCAGGGTACCGCGTGAC     360
MN401206.1         CGAGAACCTAGGGAACCCAGAGTACCGCGAGCTCCCAGAGACCCCAGGGTACCGCGTGAC     360
OP297872.1         CGAGAACCTAGGGAACCCAGAGTACCGCGAGCTCCCAGAGACCCCAGGGTACCGCGTGAC     360
HM585502.2         CGAGAACCTAGGGAACCCAGAGTACCGCGAGCTCCCAGAGACCCCAGGGTCCCGCGTGAC     360
KU310660.1         CGAGAACCTAGGGAACCCAGAGTACCGCGAGCTTCCAGAGACCCCAGGGTACCGCGTGAC     360
KU310661.1         CGAGAACCTAGGGAACCCAGAGTACCGCGAGCTTCCAGAGACCCCAGGGTACCGCGTGAC     360
DQ889502.1         CGAGAACCTAGGGAACCCAGAGTACCGCGAGCTTCCAGAGACCCCAGGGTACCGCGTGAC     360
AB618031.1         CGAGAACCTAGGGAACCCAGAGTACCGCGAGCTTCCAGAGACCCCAGGGTACCGCGTGAC     360
OP297885.1         CGAGAACCTAGGGAACCCAGAGTACCGCGAGCTTCCAGAGACCCCAGGGTACCGCGTGAC     360
ON960057.1         CGAGAACCTAGGGAACCCAGAGTACCGCGAGCTTCCAGAGACCCCAGGGTACCGCGTGAC     360
ON960061.1         CGAGAACCTAGGGAACCCAGAGTACCGCGATCTTCCAGAGACCCCAGGGTACCGCGTGAC     360
LT594111.1         CGAGAACCTAGGGAACCCAGAGTACCGCGAGCTTCCAGAGACCCCAGGGTACCGCGTGAC     360
OP297886.1         CGAGAACCTAGGGAACCCAGAGTACCGCGAGCTTCCAGAGACCCCAGGGTACCGCGTGAC     360
MG999894.1         CGAGAACCTAGGGAACCCAGAGTACCGCGAGCTTCCAGAGACCCCAGGGTACCGCGTGAC     360
KM222726.1         CGAGAACCTAGGGAACCCAGAGTACCGCGAGCTTCCAGAGACCCCAGGGTACCGCGTGAC     360
ON960056.1         CGAGAACCTAGGGAACCCAGAGTACCGCGAGCTTCCAGAGACCCCAGGGTACCGCGTGAC     360
MG999851.1         CGAGAACCTAGGGAACCCAGAGTACCGCGAGCTTCCAGAGACCCCAGGGTACCGCGTGAC     360
OP297860.1         CGAGAACCTAGGGAACCCAGAGTACCGCGAGCTTCCAGAGACCCCAGGGTACCGCGTGAC     360
                   ******************************  * **************** *********
```

```
NC_001806.2     CCCAGGGATCCACGACAACCCCGGTCTCCCAGGGAGCCCCGGTCTCCCCGGGAGCCCCGG  420
OP297876.1      CCCAGGGATCCACGACAACCCCGGTCT------------------CCCAGGGAGCCCCGG  402
KT425108.1      CCCAGGGATCCACGACAACCCCGGTCT------------------CCCAGGGAGCCCCGG  402
OP297880.1      CCCAGGGATCCACGACAACCCCGGTCT------------------CCCAGGGAGCCCCGG  402
OP297875.1      CCCAGGGATCCACGACAACCCCGGTCT------------------CCCAGGGAGCCCCGG  402
ON007163.1      CCCAGGGATCCACGACAACCCCGGTCT------------------CCCAGGGAGCCCCGG  402
ON007155.1      CCCAGGGATCCACGACAACCCCGGTCT------------------CCCAGGGAGCCCCGG  402
OP297877.1      CCCAGGGATCCACGACAACCCCGGTCT------------------CCCAGGGAGCCCCGG  402
MN401201.1      CCCAGGGATCCACGACAACCCCGGTCT------------------CCCAGGGAGCCCCGG  402
ON960054.1      CCCAGGGATCCACGACAACCCCGGTCT------------------CCCAGGGAGCCCCGG  402
ON007144.1      CCCAGGGATCCACGACAACCCCGGTCT------------------CCCAGGGAGCCCCGG  402
LT594457.1      CCCAGGGATCCACGACAACCCCGGTCT------------------CCCAGGGAGCCCCGG  402
LT594122.1      CCCAGGGATCCACGACAACCCCGGTCT------------------CCCAGGGAGCCCCGG  402
OP297863.1      CCCAGGGATCCACGACAACCCCGGTCT------------------CCCAGGGAGCCCCGG  402
ON152716.1      CCCAGGGATCCACGACAACCCCGGTCT------------------CCCAGGGAGCCCCGG  402
MH160374.1      CCCAGGGATCCACGACAACCCCGGTCT------------------CCCAGGGAGCCCCGG  402
MH999846.1      CCCAGGGATCCACGACAACCCCGGTCT------------------CCCAGGGAGCCCCGG  402
LT594108.1      CCCAGGGATCCACGACAACCCCGGTCT------------------CCCAGGGAGCCCCGG  402
MH160373.1      CCCAGGGATCCACGACAACCCCGGTCT------------------CCCAGGGAGCCCCGG  402
OP297866.1      CCCAGGGATCCACGACAACCCCGGTCT------------------CCCAGGGAGCCCCGG  402
LT594107.1      CCCAGGGATCCACGACAACCCCGGTCT------------------CCCAGGGAGCCCCGG  402
MG999856.1      CCCAGGGATCCACGACAACCCCGGTCT------------------CCCAGGGAGCCCCGG  402
MH160363.1      CCCAGGGATCCACGACAACCCCGGTCT------------------CCCAGGGAGCCCCGG  402
ON152715.1      CCCAGGGATCCACGACAACCCCGGTCT------------------CCCAGGGAGCCCCGG  402
MN401205.1      CCCAGGGATCCACGACAACCCCGGTCT------------------CCCAGGGAGCCCCGG  402
MN401207.1      CCCAGGGATCCACGACAACCCCGGTCT------------------CCCAGGGAGCCCCGG  402
MN401202.1      CCCAGGGATCCACGACAACCCCGGTCT------------------CCCAGGGAGCCCCGG  402
MG999858.1      CCCAGGGATCCACGACAACCCCGGTCT------------------CCCAGGGAGCCCCGG  402
KY922718.1      CCCAGGGATCCACGACAACCCCGGTCT------------------CCCAGGGAGCCCCGG  402
MG999891.1      CCCAGGGATCCACGACAACCCCGGTCT------------------CCCAGGGAGCCCCGG  402
KY922719.1      CCCAGGGATCCACGACAACCCCGGTCT------------------CCCAGGGAGCCCCGG  402
MN401203.1      CCCAGGGATCCACGACAACCCCGGTCT------------------CCCAGGGAGCCCCGG  402
MG999880.1      CCCAGGGATCCACGACAACCCCGGTCT------------------CCCAGGGAGCCCCGG  402
ON513441.1      CCCAGGGATCCACGACAACCCCGGTCT------------------CCCAGGGAGCCCCGG  402
MH999851.1      CCCAGGGATCCACGACAACCCCGGTCT------------------CCCAGGGAGCCCCGG  402
MN401204.1      CCCAGGGATCCACGACAACCCCGGTCT------------------CCCAGGGAGCCCCGG  402
MG999838.1      CCCAGGGATCCACGACAACCCCGGTCT------------------CCCAGGGAGCCCCGG  402
MH999842.1      CCCAGGGATCCACGACAACCCCGGTCT------------------CCCAGGGAGCCCCGG  402
ON960055.1      CCCAGGGATCCACGACAACCCCGGTCT------------------CCCAGGGAGCCCCGG  402
MN401208.1      CCCAGGGATCCACGACAACCCCGGTCT------------------CCCAGGGAGCCCCGG  402
MH999849.1      CCCAGGGATCCACGACAACCCCGGTCT------------------CCCAGGGAGCCCCGG  402
MH999845.1      CCCAGGGATCCACGACAACCCCGGTCT------------------CCCAGGGAGCCCCGG  402
OP297884.1      CCCAGGGATCCACGACAACCCCGGTCT------------------CCCAGGGAGCCCCGG  402
J02220.1        CCCAGGGATCCACGACAACCCCGGTCT------------------CCCAGGGAGCCCCGG  402
MK952183.1      CCCAGGGATCCACGACAACCCCGGTCT------------------CCCAGGGAGCCCCGG  402
LT594105.1      CCCAGGGATCCACGACAACCCCGGTCT------------------CCCAGGGAGCCCCGG  402
MK952184.1      CCCAGGGATCCACGACAACCCCGGTCT------------------CCCAGGGAGCCCCGG  402
KX424525.1      CCCAGGGATCCACGACAACCCCGGTCT------------------CCCAGGGAGCCCCGG  402
MK952185.1      CCCAGGGATCCACGACAACCCCGGTCT------------------CCCAGGGAGCCCCGG  402
OP297862.1      CCCAGGGATCCACGACAACCCCGGTCT------------------CCCAGGGAGCCCCGG  402
MN401206.1      CCCAGGGATCCACGACAACCCCGGTCT------------------CCCAGGGAGCCCCGG  402
OP297872.1      CCCAGGGATCCACGACAACCCCGGTCT------------------CCCAGGGAGCCCCGG  402
HM585502.2      CCCAGGGATCCACGACAACCCCGGTCT------------------CCCAGGGAGCCCCGG  402
KU310660.1      CCCAGGGATCCACGACAGCCCCGGTCT------------------CCCAGGGAGCCCCGG  402
KU310661.1      CCCAGGGATCCACGACAGCCCCGGTCT------------------CCCAGGGAGCCCCGG  402
DQ889502.1      CCCAGGGATCCACGACAGCCCCGGTCT------------------CCCAGGGAGCCCCGG  402
AB618031.1      CCCAGGGATCCACGACAGCCCCGGTCT------------------CCCAGGGAGCCCCGG  402
OP297885.1      CCCAGGGATCCACGACAGCCCCGGTCT------------------CCCAGGGAGCCCCGG  402
ON960057.1      CCCAGGGATCCACGACAGCCCCGGTCT------------------CCCAGGGAGCCCCGG  402
ON960061.1      CCCAGGGATCCACGACAGCCCCGGTCT------------------CCCAGGGAGCCCCGG  402
LT594111.1      CCCAGGGATCCACGACAGCCCCGGTCT------------------CCCAGGGAGCCCCGG  402
OP297886.1      CCCAGGGATCCACGACAGCCCCGGTCT------------------CCCAGGGAGCCCCGG  402
MG999894.1      CCCAGGGATCCACGACAGCCCCGGTCT------------------CCCAGGGAGCCCCGG  402
KM222726.1      CCCAGGGATCCACGACAGCCCCGGTCT------------------CCCAGGGAGCCCCGG  402
ON960056.1      CCCAGGGATCCACGACAGCCCCGGTCT------------------CCCAGGGAGCCCCGG  402
MG999851.1      CCCAGGGATCCACGACAGCCCCGGTCT------------------CCCAGGGAGCCCCGG  402
OP297860.1      CCCAGGGATCCACGACAGCCCCGGTCT------------------CCCAGGGAGCCCCGG  402
                ***************** *********                  *** ***********
```

```
NC_001806.2      TCTCCCCGGGAGCCCCGGACCCCACGCACCCCCCGCGAACCACGTACGGCTCGCGGGTCT    480
OP297876.1       TCTCCCCGGGAGCCCCGGACCCCACGCACCCCCCGCGAACCACGTACGGCTCGCGGGTCT    462
KT425108.1       TCTCCCCGGGAGCCCCGGACCCCACGCACCCCCCGCGAACCACGTACGGCTCGCGGGTCT    462
OP297880.1       TCTCCCCGGGAGCCCCGGACCCCACGCACCCCCCGCGAACCACGTACGGCTCGCGGGTCT    462
OP297875.1       TCTCCCCGGGAGCCCCGGACCCCACGCACCCCCCGCGAACCACGTACGGCTCGCGGGTCT    462
ON007163.1       TCTCCCCGGGAGCCCCGGACCCCACGCACCCCCCGCGAACCACGTACGGCTCGCGGGTCT    462
ON007155.1       TCTCCCCGGGAGCCCCGGACCCCACGCACCCCCCGCGAACCACGTACGGCTCGCGGGTCT    462
OP297877.1       TCTCCCCGGGAGCCCCGGACCCCACGCACCCCCCGCGAACCACGTACGGCTCGCGGGTCT    462
MN401201.1       TCTCCCCGGGAGCCCCGGACCCCACGCACCCCCCGCGAACCACGTACGGCTCGCGGGTCT    462
ON960054.1       TCTCCCCGGGAGCCCCGGACCCCACGCACCCCCCGCGAACCACGTACGGCTCGCGGGTCT    462
ON007144.1       TCTCCCCGGGAGCCCCGGACCCCACGCACCCCCCGCGAACCACGTACGGCTCGCGGGTCT    462
LT594457.1       TCTCCCCGGGAGCCCCGGACCCCACGCACCCCCCGCGAACCACGTACGGCTCGCGGGTCT    462
LT594122.1       TCTCCCCGGGAGCCCCGGACCCCACGCACCCCCCGCGAACCACGTACGGCTCGCGGGTCT    462
OP297863.1       TCTCCCCGGGAGCCCCGGACCCCACGCACCCCCCGCGAACCACGTACGGCTCGCGGGTCT    462
ON152716.1       TCTCCCCGGGAGCCCCGGACCCCACGCACCCCCCGCGAACCACGTACGGCTCGCGGGTCT    462
MH160374.1       TCTCCCCGGGAGCCCCGGACCCCACGCACCCCCCGCGAACCACGTACGGCTCGCGGGTCT    462
MH999846.1       TCTCCCCGGGAGCCCCGGACCCCACGCACCCCCCGCGAACCACGTACGGCTCGCGGGTCT    462
LT594108.1       TCTCCCCGGGAGCCCCGGACCCCACGCACCCCCCGCGAACCACGTACGGCTCGCGGGTCT    462
MH160373.1       TCTCCCCGGGAGCCCCGGACCCCACGCACCCCCCGCGAACCACGTACGGCTCGCGGGTCT    462
OP297866.1       TCTCCCCGGGAGCCCCGGACCCCACGCACCCCCCGCGAACCACGTACGGCTCGCGGGTCT    462
LT594107.1       TCTCCCCGGGAGCCCCGGACCCCACGCACCCCCCGCGAACCACGTACGGCTCGCGGGTCT    462
MG999856.1       TCTCCCCGGGAGCCCCGGACCCCACGCACCCCCCGCGAACCACGTACGGCTCGCGGGTCT    462
MH160363.1       TCTCCCCGGGAGCCCCGGACCCCACGCACCCCCCGCGAACCACGTACGGCTCGCGGGTCT    462
ON152715.1       TCTCCCCGGGAGCCCCGGACCCCACGCACCCCCCGCGAACCACGTACGGCTCGCGGGTCT    462
MN401205.1       TCTCCCCGGGAGCCCCGGACCCCACGCACCCCCCGCGAACCACGTACGGCTCGCGGGTCT    462
MN401207.1       TCTCCCCGGGAGCCCCGGACCCCACGCACCCCCCGCGAACCACGTACGGCTCGCGGGTCT    462
MN401202.1       TCTCCCCGGGAGCCCCGGACCCCACGCACCCCCCGCGAACCACGTACGGCTCGCGGGTCT    462
MG999858.1       TCTCCCCGGGAGCCCCGGACCCCACGCACCCCCCGCGAACCACGTACGGCTCGCGGGTCT    462
KY922718.1       TCTCCCCGGGAGCCCCGGACCCCACGCACCCCCCGCGAACCACGTACGGCTCGCGGGTCT    462
MG999891.1       TCTCCCCGGGAGCCCCGGACCCCACGCACCCCCCGCGAACCACGTACGGCTCGCGGGTCT    462
KY922719.1       TCTCCCCGGGAGCCCCGGACCCCACGCACCCCCCGCGAACCACGTACGGCTCGCGGGTCT    462
MN401203.1       TCTCCCCGGGAGCCCCGGACCCCACGCACCCCCCGCGAACCACGTACGGCTCGCGGGTCT    462
MG999880.1       TCTCCCCGGGAGCCCCGGACCCCACGCACCCCCCGCGAACCACGTACGGCTCGCGGGTCT    462
ON513441.1       TCTCCCCGGGAGCCCCGGACCCCACGCACCCCCCGCGAACCACGTACGGCTCGCGGGTCT    462
MH999851.1       TCTCCCCGGGAGCCCCGGACCCCACGCACCCCCCGCGAACCACGTACGGCTCGCGGGTCT    462
MN401204.1       TCTCCCCGGGAGCCCCGGACCCCACGCACCCCCCGCGAACCACGTACGGCTCGCGGGTCT    462
MG999838.1       TCTCCCCGGGAGCCCCGGACCCCACGCACCCCCCGCGAACCACGTACGGCTCGCGGGTCT    462
MH999842.1       TCTCCCCGGGAGCCCCGGACCCCACGCACCCCCCGCGAACCACGTACGGCTCGCGGGTCT    462
ON960055.1       TCTCCCCGGGAGCCCCGGACCCCACGCACCCCCCGCGAACCACGTACGGCTCGCGGGTCT    462
MN401208.1       TCTCCCCGGGAGCCCCGGACCCCACGCACCCCCCGCGAACCACGTACGGCTCGCGGGTCT    462
MH999849.1       TCTCCCCGGGAGCCCCGGACCCCACGCACCCCCCGCGAACCACGTACGGCTCGCGGGTCT    462
MH999845.1       TCTCCCCGGGAGCCCCGGACCCCACGCACCCCCCGCGAACCACGTACGGCTCGCGGGTCT    462
OP297884.1       TCTCCCCGGGAGCCCCGGACCCCACGCACCCCCCGCGAACCACGTACGGCTCGCGGGTCT    462
J02220.1         TCTCCCCGGGAGCCCCGGACCCCACGCACCCCCCGCGAACCACGTACGGCTCGCGGGTCT    462
MK952183.1       TCTCCCCGGGAGCCCCGGACCCCACGCACCCCCCGCGAACCACGTACGGCTCGCGGGTCT    462
LT594105.1       TCTCCCCGGGAGCCCCGGACCCCACGCACCCCCCGCGAACCACGTACGGCTCGCGGGTCT    462
MK952184.1       TCTCCCCGGGAGCCCCGGACCCCACGCACCCCCCGCGAACCACGTACGGCTCGCGGGTCT    462
KX424525.1       TCTCCCCGGGAGCCCCGGACCCCACGCACCCCCCGCGAACCACGTAGGGCTCGCGGGTCT    462
MK952185.1       TCTCCCCGGGAGCCCCGGACCCCACGCACCCCCCGCGAACCACGTACGGCTCGCGGGTCT    462
OP297862.1       TCTCCCCGGGAGCCCCGGACCCCACGCACCCCCCGCGAACCACGTACGGCTCGCGGGTCT    462
MN401206.1       TCTCCCAGGGAGCCCCGGACCCCACGCACCCCCCGCGAACCACGTACGGCTCGCGGGTCT    462
OP297872.1       TCTCCCCGGGAGCCCCGGACCCCACGCACCCCCCGCGAACCACGTACGGCTCGCGGGTCT    462
HM585502.2       TCTCCCCGGGAGCCCCGGACCCCACGCACCCCCCGCGAACCACGTACGGCTCGCGGGTCT    462
KU310660.1       CCTCCCCGGGAGCCACGGACCCCACGCACCCCCCGCGAACCACGTACGGCTCGCGGGGCT    462
KU310661.1       CCTCCCCGGGAGCCACGGACCCCACGCACCCCCCGCGAACCACGTACGGCTCGCGGGGCT    462
DQ889502.1       CCTCCCCGGGAGCCACGGACCCCACGCACCCCCCGCGAACCACGTACGGCTCGCGGGTCT    462
AB618031.1       CCTCCCCGGGAGCCACGGACCCCACGCACCCCCCGCGAACCACGTACGGCTCGCGGGTCT    462
OP297885.1       CCTCCCCGGGAGCCACGGACCCCACGCACCCCCCGCGAACCACGTACGGCTCGCGGGTCT    462
ON960057.1       CCTCCCCGGGAGCCACGGACCCCACGCACCCCCCGCGAACCACGTACGGCTCGCGGGTCT    462
ON960061.1       TCTCCCAGGGAGCCACGGACCCCACGCACCCCCCGCGAACCACGTACGGCTCGCGGGGCT    462
LT594111.1       CCTCCCCGGGAGCCACGGACCCCACGCACCCCCCGCGAACCACGTACGGCTCGCGGGTCT    462
OP297886.1       CCTCCCCGGGAGCCACGGACCCCACGCACCCCCCGCGAACCACGTACGGCTCGCGGGTCT    462
MG999894.1       CCTCCCCGGGAGCCACGGACCCCACGCACCCCCCGCGAACCACGTACGGCTCGCGGGTCT    462
KM222726.1       CCTCCCCGGGAGCCACGGACCCCACGCACCCCCCGCGAACCACGTACGGCTCGCGGGTCT    462
ON960056.1       CCTCCCCGGGAGCCACGGACCCCACGCACCCCCCGCGAACCACGTACGGCTCGCGGGTCT    462
MG999851.1       CCTCCCCGGGAGCCACGGACCCCACGCACCCCCCGCGAACCACGTACGGCTCGCGGGTCT    462
OP297860.1       CCTACCCGGGAGCCACGGACCCCACGCACCCCCCGCGAACCACGTACGGCTCGCGGGTCT    462
                  ** ** ******* ******************************* ********** **
```

NC_001806.2	GTATAG	486
OP297876.1	GTATAG	468
KT425108.1	GTATAG	468
OP297880.1	GTATAG	468
OP297875.1	GTATAG	468
ON007163.1	GTATAG	468
ON007155.1	GTATAG	468
OP297877.1	GTATAG	468
MN401201.1	GTATAG	468
ON960054.1	GTATAG	468
ON007144.1	GTATAG	468
LT594457.1	GTATAG	468
LT594122.1	GTATAG	468
OP297863.1	GTATAG	468
ON152716.1	GTATAG	468
MH160374.1	GTATAG	468
MH999846.1	GTATAG	468
LT594108.1	GTATAG	468
MH160373.1	GTATAG	468
OP297866.1	GTATAG	468
LT594107.1	GTATAG	468
MG999856.1	GTATAG	468
MH160363.1	GTATAG	468
ON152715.1	GTATAG	468
MN401205.1	GTATAG	468
MN401207.1	GTATAG	468
MN401202.1	GTATAG	468
MG999858.1	GTATAG	468
KY922718.1	GTATAG	468
MG999891.1	GTATAG	468
KY922719.1	GTATAG	468
MN401203.1	GTATAG	468
MG999880.1	GTATAG	468
ON513441.1	GTATAG	468
MH999851.1	GTATAG	468
MN401204.1	GTATAG	468
MG999838.1	GTATAG	468
MH999842.1	GTATAG	468
ON960055.1	GTATAG	468
MN401208.1	GTATAG	468
MH999849.1	GTATAG	468
MH999845.1	GTATAG	468
OP297884.1	GTATAG	468
J02220.1	GTATAG	468
MK952183.1	GTATAG	468
LT594105.1	GTATAG	468
MK952184.1	GTATAG	468
KX424525.1	GTATAG	468
MK952185.1	GTATAG	468
OP297862.1	GTATAG	468
MN401206.1	GTATAG	468
OP297872.1	GTATAG	468
HM585502.2	GTATAG	468
KU310660.1	GTATAG	468
KU310661.1	GTATAG	468
DQ889502.1	GTATAG	468
AB618031.1	GTATAG	468
OP297885.1	GTATAG	468
ON960057.1	GTATAG	468
ON960061.1	GTATAG	468
LT594111.1	GTATAG	468
OP297886.1	GTATAG	468
MG999894.1	GTATAG	468
KM222726.1	GTATAG	468
ON960056.1	GTATAG	468
MG999851.1	GTATAG	468
OP297860.1	GTATAG	468
	******	
