# Supplementary material for: Discovery of a Novel Intron in US10/US11/US12 of HSV-1 Strain 17
Source: Viruses. 2023 Oct 25;15(11):2144. doi: 10.3390/v15112144 (PMC10675037; doi:10.3390/v15112144)
Supplement: Supplementary file 1 [file viruses-15-02144-s001.zip › Supplemental Data S5. 9X_nt_ins alignment.pdf]

Supplemental Data S5: Alignment of the HSV-1 US11 Gene with the Length of Multiple 9 nt Longer Than HSV-1 Strain 17

```
NC_001806.2      ATGAGCCAGACCCAACCCCCGGCCCCAGTTGGGCCGGGCGACCCAGATGTTTACTTAAAA         60
OP297870.1       ATGAGCCAGACCCAACCCCCGGCCCCAGTTGGGCCGGGCGACCCAGATGTTTACTTAAAA         60
OQ102003.1       ATGAGCCAGACCCAACCCCCGGCCCCAGTTGGGCCGGGCGACCCAGATGTTTCCTTAAAA         60
HM585510.2       ATGAGCCAGACCCAACCCCCGGCCCCAGTTGGGCCGGGCGACCCAGATGTTTACTTAAAA         60
                 **************************************************** *******

NC_001806.2      GGCGTGCCGTCCGCCGGCATGCACCCCAGAGGTGTTCACGCACCTCGAGGACACCCGCGC         120
OP297870.1       GGCGTGCCGTCCGCCGGCATGCACCCCAGAGGTGTTCACGCACCTCGAGGACACCCGCGC         120
OQ102003.1       GGCGCGCCGTCCACCGGCATGCACCCCAGAGGTGTTCACGCACCTCGAGGACACCCGCGC         120
HM585510.2       GGCGTGCCGTCCGCCGGCATGCACCCCAGAGGTGTTCACGCACCTCGAGGACACCCGCGC         120
                 **** ******* ***********************************************

NC_001806.2      ATGATCTCCGGACCCCCGCAACGGGGTGATAATGATCAAGCGGCGGGGCAATGTGGAGAT         180
OP297870.1       ATGATCTCCGGACCCCCGCAACGGGGTGATAATGATCAAGCGGCGGGGCAATGTGGAGAT         180
OQ102003.1       ATGATCTCCGGACCCCCGCAACGGGGTGATAATGATCAAGCGGCGGGGCAATGTGGAGAT         180
HM585510.2       ATGATCTCCGGACCCCCGCAACGGGGTGATAATGATCAAGCGGCGGGGCAATGTGGAGAT         180
                 ************************************************************

NC_001806.2      TCGGGTCTACTACGAGTCGGTGCGGACACTACGATCTCGAAGCCATCTGAAGCCGTCCGA         240
OP297870.1       TCGGGTCTACTACGAGTCGGTGCGGACACTACGATCTCGAAGCCATCTGAAGCCGTCCGA         240
OQ102003.1       TCGGGTCTACTACGAGTCGGTGCGGACACTACGATCTCGAAGCCATCTGAAGCCGTCCGA         240
HM585510.2       TCGGGTCTACTACGAGTCGGTGCGGACACTACGATCTCGAAGCCATCTGAAGCCGTCCGA         240
                 ************************************************************

NC_001806.2      CCGCCAACAATCCCCAGGACACCGCGTGTTCCCCGGGAGCCCCGGGTTCCGCGACCACCC         300
OP297870.1       CCGCCAACAATCCCCAGGACACCGCGTGTTCCCCGGGAGCCCCGGGTTCCGCGACCACCC         300
OQ102003.1       CCGCCAACAATCCCCAGGACGCCGCGTGTTCCCCGGGAGCCCCGGGTTCCGCGACCACCC         300
HM585510.2       CCGCCAACATTCCCCAGGACGCCGCGTGTTCCCCGGGAGCCCCGGGTTCCGCGACCACCC         300
                 ********* ********** ***************************************

NC_001806.2      CGAGAACCTAGGGAACCCAGAGTACCGCGAGCTCCCAGAGACCCCAGGGTACCGCGTGAC         360
OP297870.1       CGAGAACCTAGGGAACCCAGAGTACCGCGAGCTCCCAGAGACCCCAGGGTACCGCGTGAC         360
OQ102003.1       CGAGAACCTAGGGAACCCAGAGTACCGCGAGCTCCCAGAGACCCCAGGGTACCGCGTGAC         360
HM585510.2       CGAGAACCTAGGGAACCCAGAGTACCGCGAGCTCCCAGAGACCCCAGGGTCCCGCGTGAC         360
                 ************************************************** *********

NC_001806.2      CCCAGGGATCCACGACAACCCCGGTCTCCCAGGGAGCCCCGGTCTCC---------CCGG         411
OP297870.1       CCCAGGGATCCACGACAACCCCGGGAGCCCCGGCCTCCCCGGGAGCCCCGGCCTCCCCGG         420
OQ102003.1       CCCAGGGATCCACGACAGTCCCGGTCTCCCCGGGAGCCCCGGGATCCACGCACGCCCCGC         420
HM585510.2       CCCAGGGATCCACGACAACCCCGGGAGCCCCGGCCTCCCCGGGATCCACGACAACCCCGG         420
                 *****************  *****   *** **   ******   **         ****

NC_001806.2      GAG------------------CCCCGGTCTCCCCGGGAGCCCCGGACCCCACGCACCCCC         453
OP297870.1       GAG------------------CCCCGGCCTCCCCGGGAGCCCCGGACCCCACGCACCCCC         462
OQ102003.1       GAACCACGACAG---------CCCCGGTCTCCCCGGGAGCCCCGGGATCCACGCACGCCC         471
HM585510.2       GAGCCCCGGCCTCCCCGGGATCCACGACAACCCCGGGAGCCCCGGACCCCACGCACCCCC         480
                 **                   **  *    ****************  ******** ***

NC_001806.2      CGCGAACCACGTACGGCTCGCGGGTCTGTATAG       486
OP297870.1       CGCGAACCACGTACGGCTCGCGGGTCTGTATAG       495
OQ102003.1       CGCGAACCACGGACGGCTCGCGGGTCTGTATAG       504
HM585510.2       CGCGAACCACGTACGGCTCGCGGGTCTGTATAG       513
                 *********** *********************
```
