# Supplementary material for: Discovery of a Novel Intron in US10/US11/US12 of HSV-1 Strain 17
Source: Viruses. 2023 Oct 25;15(11):2144. doi: 10.3390/v15112144 (PMC10675037; doi:10.3390/v15112144)
Supplement: Supplementary file 1 [file viruses-15-02144-s001.zip › Supplemental Data S6. non_9_gaps.alignment.pdf]

Supplemental Data S6: Alignment of the HSV-1 US11 Gene Non Multiple 9 nt shorter Than US11 Gene in HSV-1 Strain 17

```
NC_001806.2      ATGAGCCAGACCCAACCCCCGGCCCCAGTTGGGCCGGGCGACCCAGATGTTTACTTAAAA         60
MH160377.1       ATGAGCCAGACCCAACCCCCGGCCCCAGTTGGGCCGGGCGACCCAGATGTTTACTTAAAA         60
MG999862.1       ATGAGCCAGACCCAACCCCCGGCCCCAGTTGGGCCGGGCGACCCAGATGTTTACTTAAAA         60
MG999868.1       ATGAGCCAGACCCAACCCCCGGCCCCAGTTGGGCCGGGCGACCCAGATGTTTACTTAAAA         60
MG999884.1       ATGAGCCAGACCCAACCCCCGGCCCCAGTTGGGCCGGGCGACCCAGATGTTTACTTAAAA         60
                 ************************************************************

NC_001806.2      GGCGTGCCGTCCGCCGGCATGCACCCCAGAGGTGTTCACGCACCTCGAGGACACCCGCGC         120
MH160377.1       GGCGTGCCGTCCGCCGGCATGCACCCCAGAGGTGTTCACGCACCTCGAGGACACCCGCGC         120
MG999862.1       GGCGTGCCGTCCGCCGGCATGCACCCCAGAGGTGTTCACGCACCTCGAGGACACCCGCGC         120
MG999868.1       GGCGTGCCGTCCGCCGGCATGCACCCCAGAGGTGTTCACGCACCTCGAGGACACCCGCGC         120
MG999884.1       GGCGTGCCGTCCGCCGGCATGCACCCCAGAGGTGTTCACGCACCTCGAGGACACCCGCAC         120
                 **********************************************************.*

NC_001806.2      ATGATCTCCGGACCCCCGCAACGGGGTGATAATGATCAAGCGGCGGGGCAATGTGGAGAT         180
MH160377.1       ATGATCTCCGGACCCCCGCAACGGGGTGATAATGATCAAGCGGCGGGGCAATGTGGAGAT         180
MG999862.1       ATGATCTCCGGACCCCCGCAACGGGGTGATAATGATCAAGCGGCGGGGCAATGTGGAGAT         180
MG999868.1       ATGATCTCCGGACCCCCGCAACGGGGTGATAATGATCAAGCGGCGGGGCAATGTGGAGAT         180
MG999884.1       ATGATCTCCGGATCCCCGCAACGGGGTGATAATGATCAAGCGGCGGGGCAATGTGGAGAT         180
                 ************ ***********************************************

NC_001806.2      TCGGGTCTACTACGAGTCGGTGCGGACACTACGATCTCGAAGCCATCTGAAGCCGTCCGA         240
MH160377.1       TCGGGTCTACTACGAGTCGGTGCGGACACTACGATCTCGAAGCCATCTGAAGCCGTCCGA         240
MG999862.1       TCGGGTCTACTACGAGTCGGTGCGGACACTACGATCTCGAAGCCATCTGAAGCCGTCCGA         240
MG999868.1       TCGGGTCTACTACGAGTCGGTGCGGACACTACGATCTCGAAGCCATCTGAAGCCGTCCGA         240
MG999884.1       TCGGGTCTACTACGAGTCGGTGCGGACACTACGATCTCGAAGCCATCTGAAGCCGTCCGA         240
                 ************************************************************

NC_001806.2      CCGCCAACAATCCCCAGGACACCGCGTGTTCCCCGGGAGCCCCGGGTTCCGCGACCACCC         300
MH160377.1       CCGCCAACAATCCCCAGGACACCGCGTGTTCCCCGGGAGCCCCGGGTTCCGCGACCACCC         300
MG999862.1       CCGCCAACAATCCCCAGGACACCGCGTGTTCCCCGGGAGCCCCGGGTTCCGCGACCACCC         300
MG999868.1       CCGCCAACAATCCCCAGGACACCGCGTGTTCCCCGGGAGCCCCGGGTTCCGCGACCACCC         300
MG999884.1       CCGCCAACAATCCCCAGGACACCGCGTGTTCCCCGGGAGCCCCGGGTTCCGCGACCACCC         300
                 ************************************************************

NC_001806.2      CGAGAACCTAGGGAACCCAGAGTACCGCGAGCTCCCAGAGACCCCAGGGTACCGCGTGAC         360
MH160377.1       CGAGAACCTAGGGAACCCAGAGTACCGCGAGCTCCCAGAGACCCCAGGGTACCGCGTGAC         360
MG999862.1       CGAGAACCTAGGGAACCCAGAGTACCGCGAGCTCCCAGAGACCCCAGGGTACCGCGTGAC         360
MG999868.1       CGAGAACCTAGGGAACCCAGAGTACCGCGAGCTCCCAGAGACCCCAGGGTACCGCGTGAC         360
MG999884.1       CGAGAACCTAGGGAACCCAGAGTACCGCGAGCTCCCAGAGACCCCAGGGTACCGCGTGAC         360
                 ************************************************************

NC_001806.2      CCCAGGGATCCACGACAACCCCGGTCTCCCAGGGAGCCCCGGTCTCCCCGGGAGCCCCGG         420
MH160377.1       CCCAGGGATCCACGACAACCCCGGC---------------------CCCGGGAGCCCCGG         399
MG999862.1       CCCAGGGATCCACGACAACCCCGGC---------------------CCCGGGAGCCCCGG         399
MG999868.1       CCCAGGGATCCACGACAACCCCGGC------------------------GGGAGCCCCGG         396
MG999884.1       CCCAGGGATCCACGACAACCCCGGC---------------------CCCGGGAGCCCCGG         399
                 ************************                        ************

NC_001806.2      TCTCCCCGGGAGCCCCGGACCCCACGCACCCCCCGCGAACCACGTACGGCTCGCGGGTCT         480
MH160377.1       CCTCCCCGGGAGCCCCGGACCCCACGCACCCCCCGCGAACCACGTACGGCTCGCGGGTCT         459
MG999862.1       CCTCCCCGGGAGCCCCGGACCCCACGCACCCCCCGCGAACCACGTACGGCTCGCGGGTCT         459
MG999868.1       CCTCCCCGGGAGCCCCGGACCCCACGCACCCCCCGCGAACCACGTACGGCTCGCGGGTCT         456
MG999884.1       CCTCCCCGGGAGCCCCGGACCCCACGCACCCCCCGCAAACCACGTACGGCTCGCGGGTCT         459
                  ***********************************.***********************

NC_001806.2      GTATAG      486
MH160377.1       GTATAG      465
MG999862.1       GTATAG      465
MG999868.1       GTATAG      462
MG999884.1       GTATAG      465
                 ******
```
