# Supplementary material for: Discovery of a Novel Intron in US10/US11/US12 of HSV-1 Strain 17
Source: Viruses. 2023 Oct 25;15(11):2144. doi: 10.3390/v15112144 (PMC10675037; doi:10.3390/v15112144)
Supplement: Supplementary file 1 [file viruses-15-02144-s001.zip › Supplemental Data S7. non_3_gaps.alignment.pdf]

## Supplemental Data S7: Alignment of the HSV-1 US11 Gene Non Multiple 3 nt shorter Than US11 Gene in HSV-1 Strain 17

```
NC_001806.2      ATGAGCCAGACCCAACCCCCGGCCCCAGTTGGGCCGGGCGACCCAGATGTTTACTTAAAA         60
MG999855.1       ATGAGCCAGACCCAACCCCCGGCCCCAGTTGGGCCGGGCGACCCAGATGTTTACTTAAAA         60
MG999853.1       ATGAGCCAGACCCAACCCCCGGCCCCAGTTGGGCCGGGCGACCCAGATGTTTACTTAAAA         60
MH160367.1       ATGAGCCAGACCCAACCCCCGGCCCCAGTTGGGCCGGGCGACCCAGATGTTTACTTAAAA         60
                 ************************************************************

NC_001806.2      GGCGTGCCGTCCGCCGGCATGCACCCCAGAGGTGTTCACGCACCTCGAGGACACCCGCGC         120
MG999855.1       GGCGTGCCGTCCGCCGGCATGCACCCCAGAGGTGTTCACGCACCTCGAGGACACCCGCGC         120
MG999853.1       GGCGTGCCGTCCGCCGGCATGCACCCCAGAGGTGTTCACGCACCTCGAGGACACCCGCGC         120
MH160367.1       GGCGTGCCGTCCGCCGGCATGCACCCCAGAGGTGTTCACGCACCTCGAGGACACCCGCAC         120
                 ********************************************************** *

NC_001806.2      ATGATCTCCGGACCCCCGCAACGGGGTGATAATGATCAAGCGGCGGGGCAATGTGGAGAT         180
MG999855.1       ATGATCTCCGGACCCCCGCAACGGGGTGATAATGATCAAGCGGCGGGGCAATGTGGAGAT         180
MG999853.1       ATGATCTCCGGACCCCCGCAACGGGGTGATAATGATCAAGCGGCGGGGCAATGTGGAGAT         180
MH160367.1       CTGATCTCCGGACCCCCGCAACGGGGTGATAATGATCAAGCGGCGGGGCAATGTGGAGAT         180
                  ***********************************************************

NC_001806.2      TCGGGTCTACTACGAGTCGGTGCGGACACTACGATCTCGAAGCCATCTGAAGCCGTCCGA         240
MG999855.1       TCGGGTCTACTACGAGTCGGTGCGGACACTACGATCTCGAAGCCATCTGAAGCCGTCCGA         240
MG999853.1       TCGGGTCTACTACGAGTCGGTGCGGACACTACGATCTCGAAGCCATCTGAAGCCGTCCGA         240
MH160367.1       TCGGGTCTACTACGAGTCGGTGCGGACACTACGATCTCGAAGCCATCTGAAGCCGTCCGA         240
                 ************************************************************

NC_001806.2      CCGCCAACAATCCCCAGGACACCGCGTGTTCCCCGGGAGCCCCGGGTTCCGCGACCACCC         300
MG999855.1       CCGCCAACAATCCCCAGGACACCGCGTGTTCCCCGGGAGCCCCGGGTTCCGCGACCACCC         300
MG999853.1       CCGCCAACAATCCCCAGGACACCGCGTGTTCCCCGGGAGCCCCGGGTTCCGCGACCACCC         300
MH160367.1       CCGCCAACAATCCCCAGGACACCGCGTGTTCCCCGGGAGCCCCGGGTTCCGCGACCACCC         300
                 ************************************************************

NC_001806.2      CGAGAACCTAGGGAACCCAGAGTACCGCGAGCTCCCAGAGACCCCAGGGTACCGCGTGAC         360
MG999855.1       CGAGAACCTAGGGAACCCAGAGTACCGCGAGCTCCCAGAGACCCCAGGGTACCGCGTGAC         360
MG999853.1       CGAGAACCTAGGGAACCCAGAGTACCGCGAGCTCCCAGAGACCCCAGGGTACCGCGTGAC         360
MH160367.1       CGAGAACCTAGGGAACCCAGAGTACCGCGAGCTCCCAGAGACCCCAGGGTACCGCGTGAC         360
                 ************************************************************

NC_001806.2      CCCAGGGATCCACGACAACCCCGGTCTCCCAGGGAGCCCCGGTCTCCCCGGGAGCCCCGG         420
MG999855.1       CCCAGGGATCCACGACAACCCCGG----------------------CCCGGGAGCCCCGG         398
MG999853.1       CCCAGGGATCCACGACAACCCCGG-------------------------GGGAGCCCCGG         395
MH160367.1       CCCAGGGATCCACGACAACCCCGG-----------------------CCGGGAGCCCCGG         397
                 ***********************                          ***********

NC_001806.2      TCTCCCCGGGAGCCCCGGACCCCACGCACCCCCCGCGAACCACGTACGGCTCGCGGGTCT         480
MG999855.1       CCTCCCCGGGAGCCCCGGACCCCACGCACCCCCCGCGAACCACGTACGGCTCGCGGGTCT         458
MG999853.1       CCTCCCCGGGAGCCCCGGACCCCACGCACCCCCCGCGAACCACGTACGGCTCGCGGGTCT         455
MH160367.1       CCTCCCCGGGAGCCCCGGACCCCACGCACCCCCCGCGAACCACGTACGGCTCGCGGGTCT         457
                  ***********************************************************

NC_001806.2      GTATAG      486
MG999855.1       GTATAG      464
MG999853.1       GTATAG      461
MH160367.1       GTATAG      463
                 ******
```
