# Supplementary material for: Discovery of a Novel Intron in US10/US11/US12 of HSV-1 Strain 17
Source: Viruses. 2023 Oct 25;15(11):2144. doi: 10.3390/v15112144 (PMC10675037; doi:10.3390/v15112144)
Supplement: Supplementary file 1 [file viruses-15-02144-s001.zip › Supplemental Methods and Figures.pdf]

SUPPLEMENTAL METHODS AND FIGURES

Supplemental Methods

Computational modeling of US11 structure

The mature US11 protein sequence of HSV-1 strain 17 was obtained by deleting the 27 nt intron identified in this study from its annotated coding sequence in the NCBI GenBank record (NCBI Acc#: NC_001806.2) which was then translated into the protein sequence. To investigate the US11 structural features, both 2D and 3D modeling techniques were performed. The secondary structure prediction methods from multiple sources including PSIPRED [1], PROTEUS2 [2], and NetSurfP-3.0 [3] were used with the US11 protein sequence as the input. The results from all 2D modeling methods show US11 has disordered features in almost the whole sequence, which indicates that its 3D structure determination will be difficult, further evidenced by the fact that no templates with significant similarity to US11 were found when NCBI blast was performed against the PDB database. Therefore, multiple de-novo 3D structure modeling tools including trRosetta [4], Robetta [5] and QUARK [6] were used to perform the ab initio prediction. After manual inspection of the returned results, the top trRosetta result was selected as the final representative US11 model due to the fact that trRosetta returned relatively stable results (top 5) by comprising counterparts. In addition, trRosetta was reported to be one of the top servers in terms of rapid and accurate de novo structure prediction [4]. trRosetta first takes the input of a protein sequence, and a deep residual neural network is applied to predict the inter-residue distance and orientation distributions, which are then transformed as restraints to guide the structure prediction based on direct energy minimization under the framework of Rosetta [4]. In the current work, the default option was used to predict the US11 3D structure. SWISS-MODEL was finally employed to perform further structure refinement [7]. It should be noted that due to the disordered property of US11, the predicted model should not be considered high confidence but one of many possibilities of structural conformations (**Supplemental Figure S3**).

1. McGuffin, L. J.; Bryson, K.; Jones, D. T., The PSIPRED protein structure prediction server. *Bioinformatics* **2000,** 16, (4), 404-405.
2. Montgomerie, S.; Cruz, J. A.; Shrivastava, S.; Arndt, D.; Berjanskii, M.; Wishart, D. S., PROTEUS2: a web server for comprehensive protein structure prediction and structure-based annotation. *Nucleic acids research* **2008,** 36, (suppl_2), W202-W209.
3. Høie, M. H.; Kiehl, E. N.; Petersen, B.; Nielsen, M.; Winther, O.; Nielsen, H.; Hallgren, J.; Marcatili, P., NetSurfP-3.0: accurate and fast prediction of protein structural features by protein language models and deep learning. *Nucleic acids research* **2022,** 50, (W1), W510-W515.
4. Du, Z.; Su, H.; Wang, W.; Ye, L.; Wei, H.; Peng, Z.; Anishchenko, I.; Baker, D.; Yang, J., The trRosetta server for fast and accurate protein structure prediction. *Nature protocols* **2021,** 16, (12), 5634-5651.
5. Kim, D. E.; Chivian, D.; Baker, D., Protein structure prediction and analysis using the Robetta server. *Nucleic acids research* **2004,** 32, (suppl_2), W526-W531.
6. Xu, D.; Zhang, Y., Ab initio protein structure assembly using continuous structure fragments and optimized knowledge-based force field. *Proteins: Structure, Function, and Bioinformatics* **2012,** 80, (7), 1715-1735.
7. Waterhouse, A.; Bertoni, M.; Bienert, S.; Studer, G.; Tauriello, G.; Gumienny, R.; Heer, F. T.; de Beer, T. A. P.; Rempfer, C.; Bordoli, L.; Lepore, R.; Schwede, T., SWISS-MODEL: homology modelling of protein structures and complexes. *Nucleic Acids Res* **2018,** 46, (W1), W296-w303.

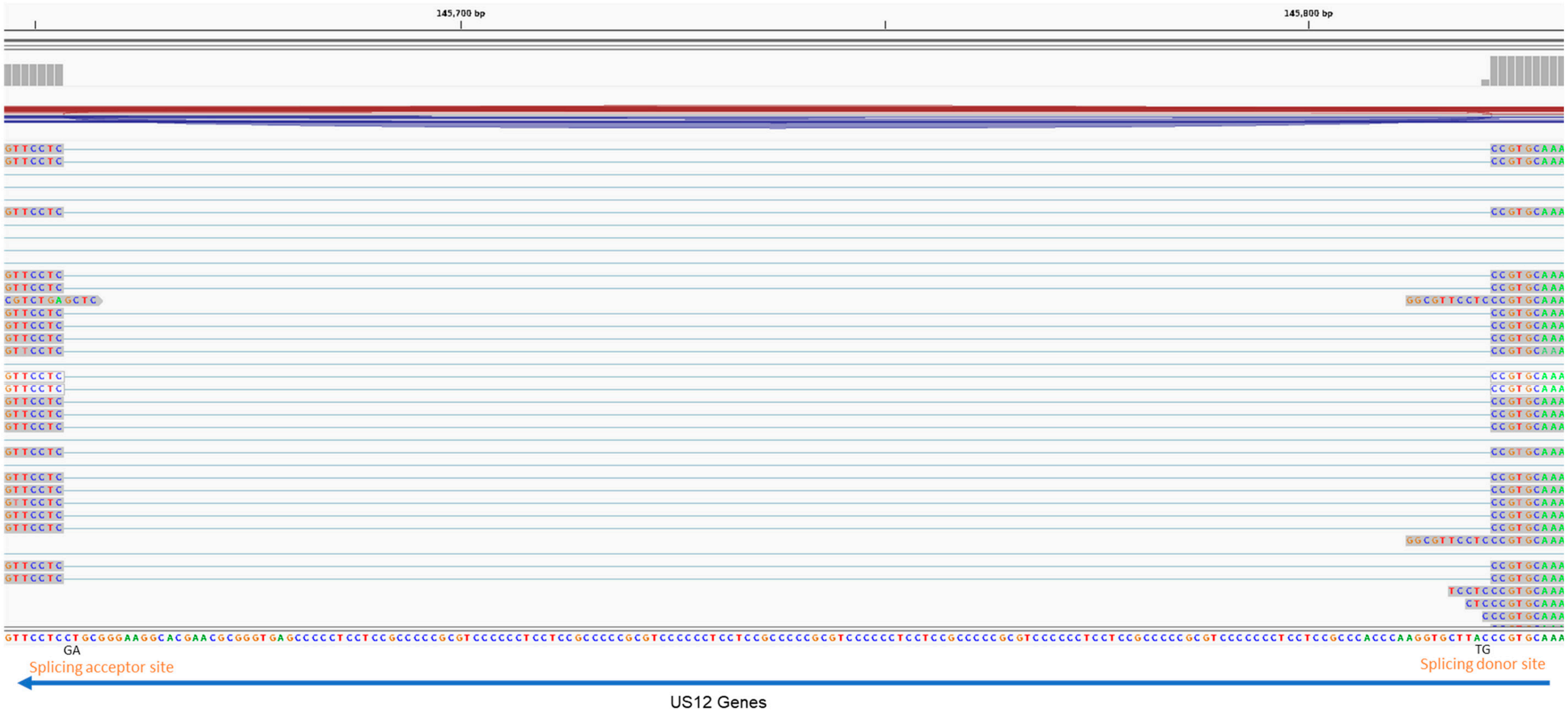

Figure S1. A zoomed-in view of the region of US12 intron within 5′ UTR. the splicing donor site GT (complemented strand of AC) and acceptor site AG (complemented strand of CT) were located at the border of the intron and labeled. US12 is on the complement strand of the reference sequence and the gene direction is indicated by the arrow.

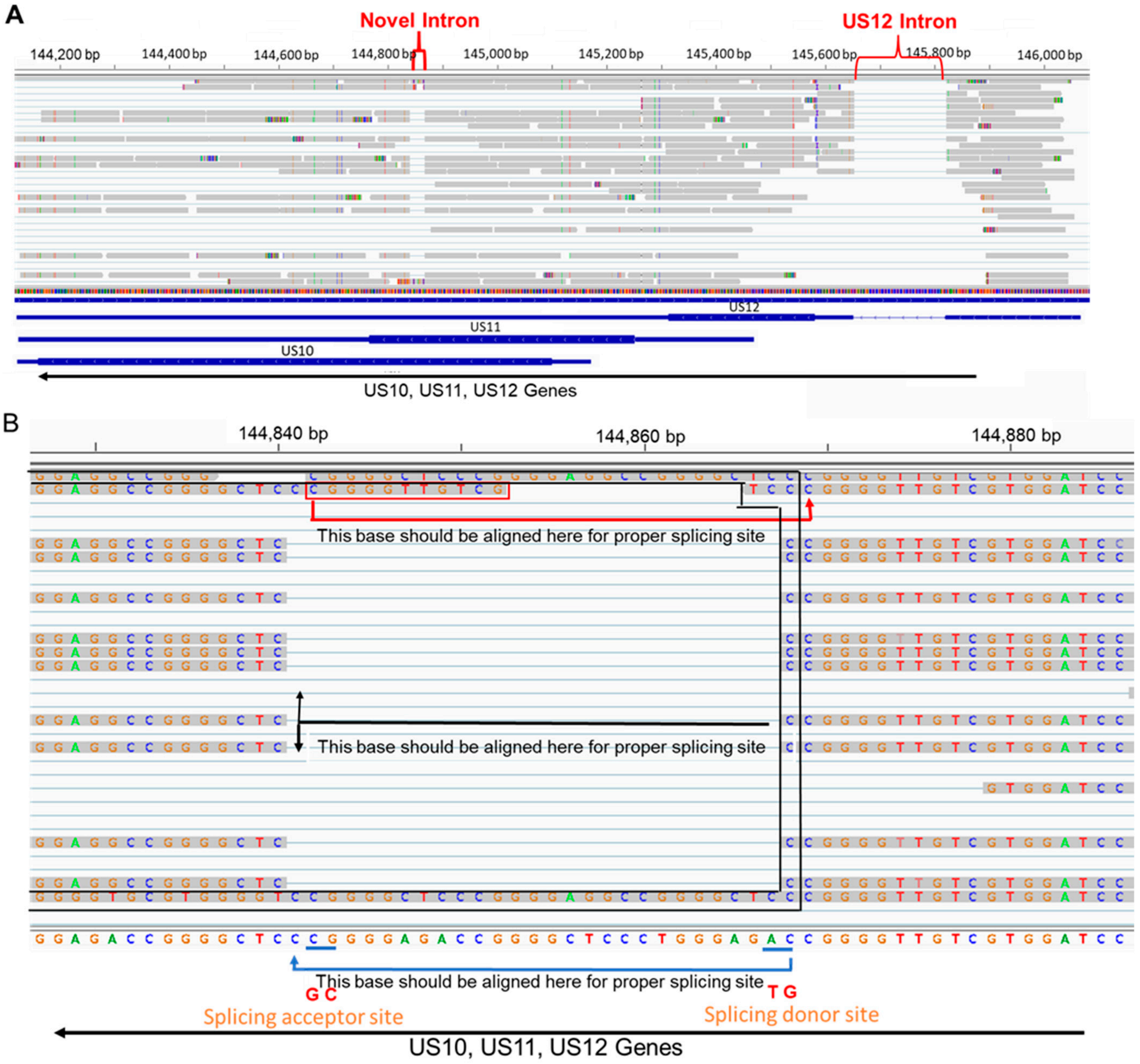

Figure S2. (To be continued)

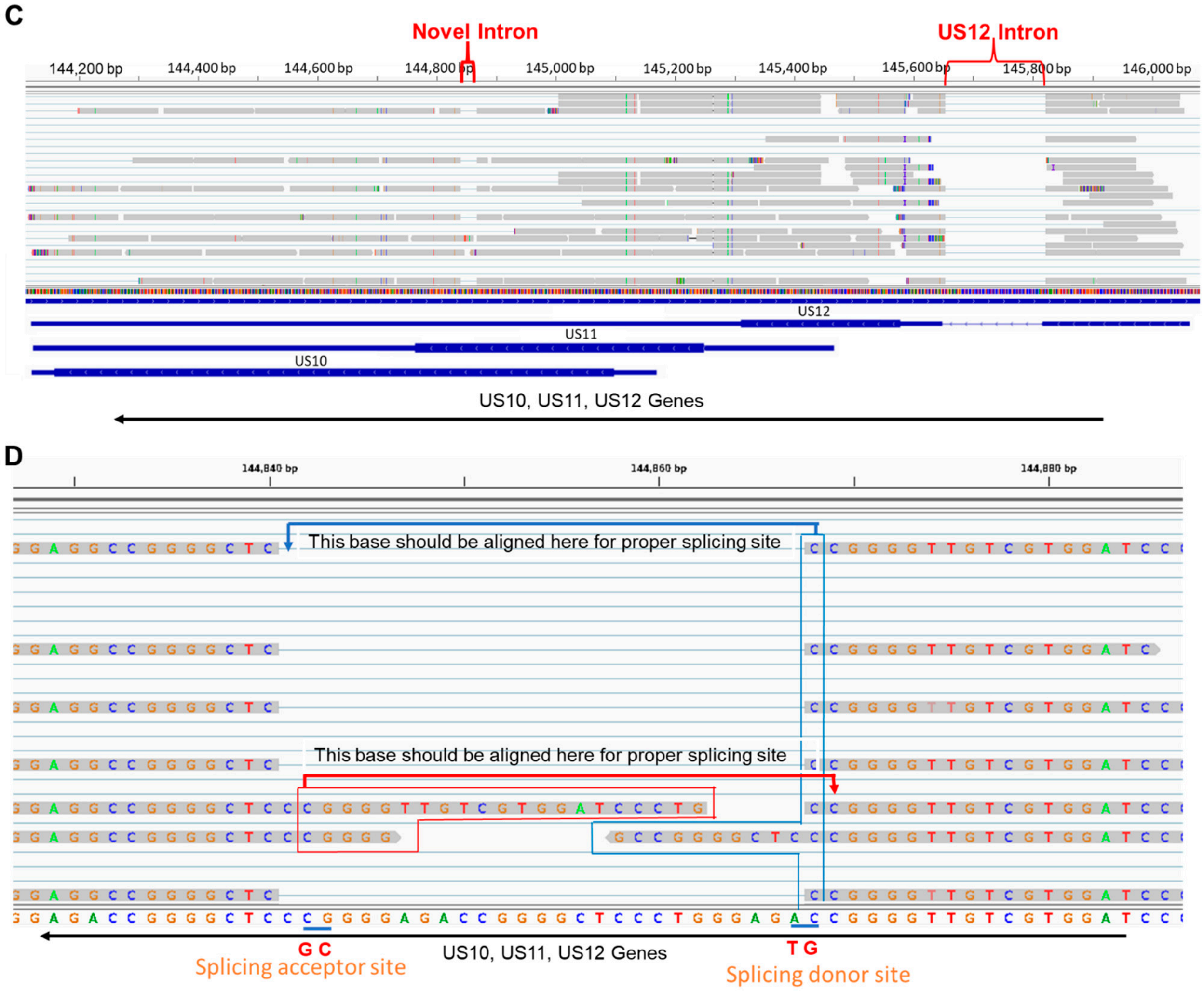

Figure S2. Identification of a Novel Intron in US10, US11 and US12 Genes of HSV-1 Strain 17. The RNA-Seq reads from two HSV-1 strain 17 infected human fibroblast KMB17 cell culture samples (SRR6029569: **A** and **B**; SRR6029570: **C** and **D**) were aligned with the reference sequence, HSV-1 strain 17 genome sequence (NCBI ACC#: NC_001806.2), to visualize the intron within US10/US11/US12 genes. The novel intron in US10/US11/US12 and well-known US12 intron were marked with red brackets. The annotated gene structure of US10, US11 and US12 in reference were shown in the bottom of the panel **A** and **C**. Thick bar: coding sequence, narrow bar: untranslated exon sequence, line: intron. These genes are on the complement strand of the reference sequence.

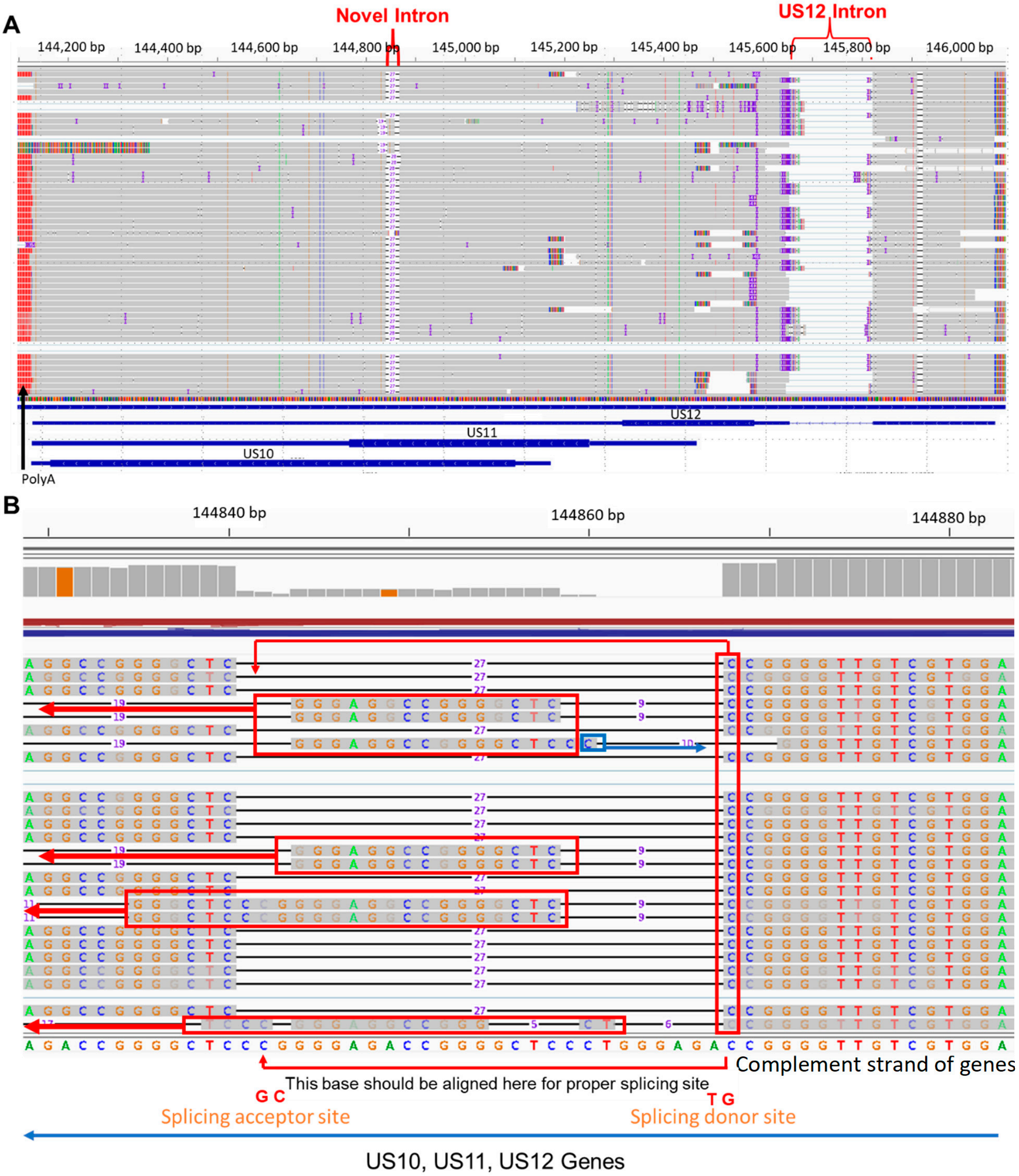

Figure S3. Identification of a Novel Intron in US10/US11/US12 Genes of *HSV-1* Strain 17: (**A**) Combined RNA-Seq sequencing reads with PacBio SMRT platform from six HSV-1 strain 17-infected Vero cell culture samples (NCBI BioProject #: PRJNA382882) were aligned with the reference sequence, HSV-1 strain 17 genome sequence (NCBI ACC#: NC_001806.2) and visualized with IGV. The novel intron in US10/US11/US12 and well-known US12 intron were shown with red brackets. The gene structure of US10, US11 and US12 were shown in the bottom of the panel. These genes are on the complement strand of the reference sequence. (**B**) A zoomed-in view of the region of the newly identified novel intron. After we move base "C" on right side of the gap to the left side, the splicing donor site GT (complement of AC) and acceptor site CG (complemented s of CG) were shown at the border of the novel intron. Some read sequences within the intron could be moved properly as shown in the figure, consistent with the intron structure.

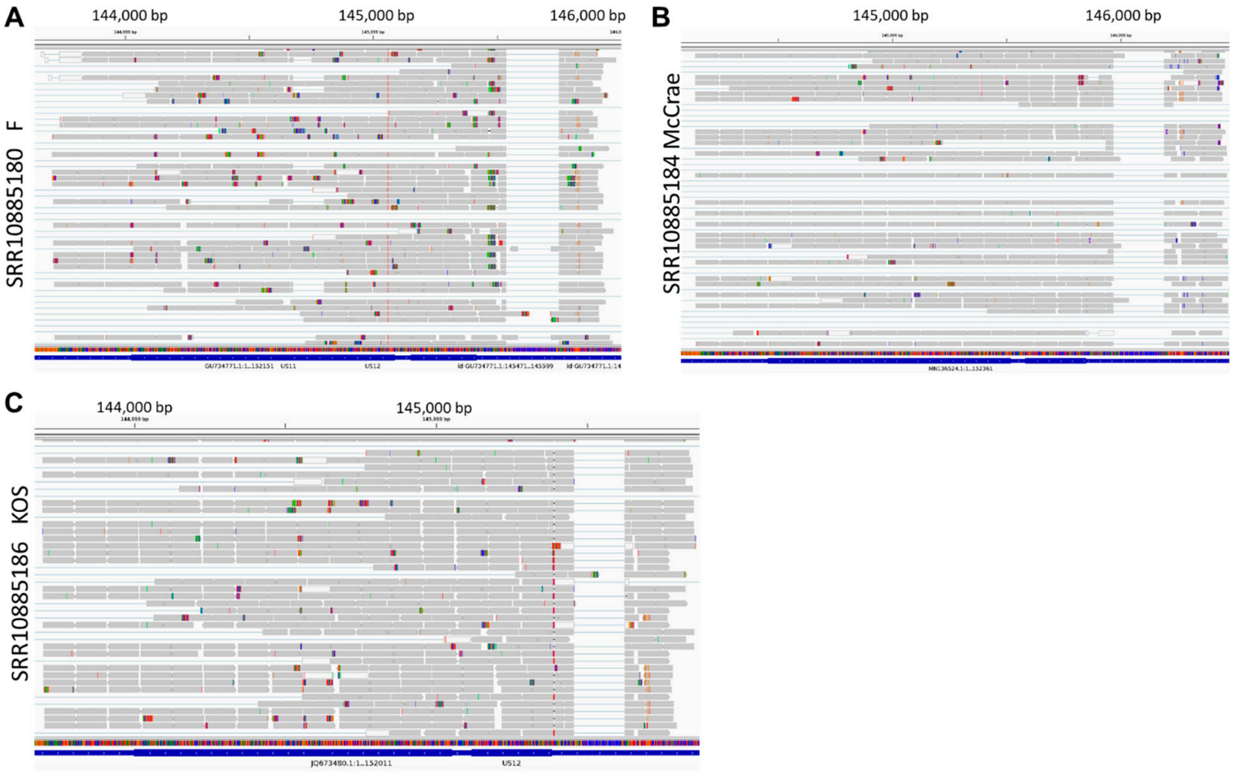

Figure S4. No New Intron Identified in US10/US11/US12 Genes in HSV-1 Strain F, McKrae, and KOS. (**A**) The alignment of the RNA-Seq sequencing reads from an HSV-1 strain F infected human neuron cell culture sample (NCBI SRA#: SRR10885180) with its genome sequence (NCBI ACC#: GU734771.1). (**B**) The alignment of the RNA-Seq sequencing reads from the HSV-1 strain McKrae-infected human neuron cell culture sample (NCBI SRA#: SRR10885184) with its genome sequence (NCBI ACC#: MN136524.1). (**C**) The alignment of the RNA-Seq sequencing reads from the HSV-1 strain KOS-infected human neuron cell culture sample (NCBI SRA#: SRR10885186) with its genome sequence (NCBI ACC#: JQ780693.1).

A

```
Strain_17_S MIKRRGNVEIRVYYESVRTLRSRSHLKPSDRQQSPGHRVFPGSPGFRDHPENLGNPEYRE 60
HSV-N-7     MIKRRGNVEIRVYYESVRTLRSRSHLKPSDRQQSPGHRVFPGSPGFRDHPENLGNPEYRE 60
Strain_ES   MIKRRGNVEIRVYYESVRTLRSRSHLKPSDRQHSPGHRVFPGSPGFRDHPENLGNPEYRE 60
OP297870.1  MIKRRGNVEIRVYYESVRTLRSRSHLKPSDRQQSPGHRVFPGSPGFRDHPENLGNPEYRE 60
OQ102003.1  MIKRRGNVEIRVYYESVRTLRSRSHLKPSDRQQSPGRRVFPGSPGFRDHPENLGNPEYRE 60
HM585510.2  MIKRRGNVEIRVYYESVRTLRSRSHLKPSDRQHSPGRRVFPGSPGFRDHPENLGNPEYRE 60
            ********************************:***:***********************

Strain_17_S LPETPGYRVTPGIHDNPG------------------SPGLPGSPGPHAPPANHVRLAGLY 102
HSV-N-7     LPETPGYRVTPGIHDNPGLPG---------------SPGLPGSPGPHAPPANHVRLAGLY 105
Strain_ES   LPETPGYRVTPGIHDNPGSPGLPG------------SPGLPGSPGPHAPPANHVRLAGLY 108
OP297870.1  LPETPGYRVTPGIHDNPGSPGLPGSPGLPG------SPGLPGSPGPHAPPANHVRLAGLY 114
OQ102003.1  LPETPGYRVTPGIHDSPGLPGSPGIHARPANHD---SPGLPGSPGIHARPANHGRLAGLY 117
HM585510.2  LPETPGSRVTPGIHDNPGSPGLPGIHDNPGSPGLPGIHDNPGSPGPHAPPANHVRLAGLY 120
            ****** ********.**                      ***** ** **** ******

Strain_17_S SPGKYAPLASPDPFSPQHGAYARARVGIHTAVRVPPTGSPTHTHLRQDPGDEPTSDDSGL 162
HSV-N-7     SPGKYAPLASPDPFSPQHGAYARARVGIHTAVRVPPTGSPTHTHLRQDPGDEPTSDDSGL 165
Strain_ES   SPGKYAPLASPDPFSPQDGAYARARVGLHTAVRVPPTGSPTHTHLRHDPGDEPTSDDSGL 168
OP297870.1  SPGKYAPLASPDPFSPQDGAYARARVGLHTAVRVPPTGSPTHTHLRHDPGDEPTSDDSGL 174
OQ102003.1  SPGKYAPLASPDPFSPQDGAYARARVGIHTAVRVPPTGSPTHTHLRHDPGDEPTSDDSGL 177
HM585510.2  SPGKYAPLASPDPFSPQDGAYARARVGLHTAVRVPPTGSPTHTHLRHDPGDEPTSDDSGL 180
            *****************.*********:******************:*************

Strain_17_S YPLDARALAHLVMLPADHRAFFRTVVEVSRMCAANVRDPPPPATGAMLGRHARLVHTQWL 222
HSV-N-7     YPLDARALAHLVMLPTDHRAFFRTVVEVSRMCAANVRDPPPPATGAMLGRHARLVHTQWL 225
Strain_ES   YPLDARALAHLVMLPADHRAFFRTVVDVSRMCAANVRDPPPPATGAMLGRHARLVHTQWL 228
OP297870.1  YPLDARALAHLVMLPADHRAFFRTVVDVSRMCAANVRDPPPPATGAMLGRHARLVHTQWL 234
OQ102003.1  YPLDARALAHLVMLPADHRAFFRTVVEVSRMCAANVRDPPPPATGAMLGRHARLVHTQWL 237
HM585510.2  YPLDARALAHLVMLPADHRAFFRTVVDVSRMCAANVRDPPPPATGAMLGRHARLVHTQWL 240
            ***************:**********:*********************************

Strain_17_S RANQETSPLWPWRTAAINFITTMAPRVQTHRHMHDLLMACAFWCCLTHASTCSYAGLYST 282
HSV-N-7     RANQETSPLWPWRTAAINFITTMAPRVQTHRHMHDLLMACAFWCCLTHASTCSYAGLYST 285
Strain_ES   RANQETSPLWPWRTAAINFITTMAPRVQTHRHMHDLLMACAFWCCLTHASTCSYAGLYST 288
OP297870.1  RANQETSPLWPWRTAAINFITTMAPRVQTHRHMHDLLMACAFWCCLTHASTCSYAGLYST 294
OQ102003.1  RANRETSPLWPWRTAAINFITTMAPRVQTHRHMHDLLMACAFWCCLTHASTCSYAGLYST 297
HM585510.2  RANQETSPLWPWRTAAINFITTMAPRVQTHRHMHDLLMACAFWCCLTHASTCSYAGLYST 300
            ***:********************************************************

Strain_17_S HCLHLFGAFGCGDPALTPPLC------ 303
HSV-N-7     HCLHLFGAFGCGDPALTPPL------- 305
Strain_ES   HCLHLFGAFGCGDPALTPPLC------ 309
OP297870.1  HCLHLFGAFGCGDPALTPPLC------ 315
OQ102003.1  HCLHLFGAFGCGAPALTPPLCQGNLCP 324
HM585510.2  HCLHLFGAFGCGDPALTPPLC------ 321
            ************ *******
```

B

```
Strain_17_S MIKRRGNVEIRVYYESVRTLRSRSHLKPSDRQQSPGHRVFPGSPGFRDHPENLGNPEYRE 60
HM585508.2  MIKRRGNVEIRVYYESVRTLRSRSHLKPSDRQQSPGHRVFPGSPGFRDHPENLGNPEYRE 60
KT425110.1  MIKRRGNVEIRVYYESVRTLRSRSHLKPSDRQQSPGHRVFPGSPGFRDHPENLGNPEYRE 60
MF156584.1  MIKRRGNVEIRVYYESVRTLRSRSHLKPSDRQQSPGHRVFPGSPGFRDHPENLGNPEYRE 60
LT594110.1  MIKRRGNVEIRVYYESVRTLRSRSHLKPSDRQQSPGHRVFPGSPGFRDHPENLGNPEYRE 60
KJ847330.1  MIKRRGNVEIRVYYESVRTLRSRSHLKPSDRQQSPGHRVFPGSPGFRDHPENLGNPEYRE 60
JQ780693.1  MIKRRGNVEIRVYYESVRTLRSRSHLKPSDRQQSPGHRVFPGSPGFRDHPENLGNPEYRE 60
KT887224.1  MIKRRGNVEIRVYYESVRTLRSRSHLKPSDRQQSPGHRVFPGSPGFRDHPENLGNPEYRE 60
KT780616.1  MIKRRGNVEIRVYYESVRTLRSRSHLKPSDRQQSPGHRVFPGSPGFRDHPENLGNPEYRE 60
MH480513.1  MIKRRGNVEIRVYYESVRTLRSRSHLKPSDRQQSPGHRVFPGSPGFRDHPENLGNPEYRE 60
            ************************************************************

Strain_17_S LPETPGYRVTPGIHDNPGSPGLPGSPGPHAPPANHVRLAGLYSPGKYAPLASPDPFSPQH 120
HM585508.2  LPETPGYRVTPGIHDNPG---LPGSPGPHAPPANHVRLAGLYSPGKYAPLASPDPFSPQH 117
KT425110.1  LPETPGYRVTPGIHDNPG---LPGSPGPHAPPANHVRLAGLYSPGKYAPLASPDPFSPQD 117
MF156584.1  LPETPGYRVTPGIHDNPG---LPGSPGPHAPPANHVRLAGLYSPGKYAPLASPDPFSPQD 117
LT594110.1  LPETPGYRVTPGIHDNPG---LPGSPGPHAPPANHVRLAGLYSPGKYAPLASPDPFSPQH 117
KJ847330.1  LPETPGYRVTPGIHDNPG---LPGSPGPHAPPANHVRLAGLYSPGKYAPLASPDPFSPQH 117
JQ780693.1  LPETPGYRVTPGIHDNPG---LPGSPGPHAPPANHVRLAGLYSPGKYAPLASPDPFSPQD 117
KT887224.1  LPETPGYRVTPGIHDNPG---LPGSPGPHAPPANHVRLAGLYSPGKYAPLASPDPFSPQD 117
KT780616.1  LPETPGYRVTPGIHDNPG---LPGSPGPHAPPANHVRLAGLYSPGKYAPLASPDPFSPQH 117
MH480513.1  LPETPGYRVTPGIHDNPG---LPGSPGPHAPPANHVRLAGLYSPGKYAPLASPDPFSPQH 117
            ******************   **************************************.

Strain_17_S GAYARARVGIHTAVRVPPTGSPTHTHLRQDPGDEPTSDDSGLYPLDARALAHLVMLPADH 180
HM585508.2  GAYARARVGIHTAVRVPPTGSPTHTHLRQDPGDEPTSDDSGLYPLDARALAHLVMLPADH 177
KT425110.1  GAYARARVGIHTAVRVPPTGSPTHTHLRQDPGDEPTSDDSGLYPLDARALAHLVMLPADH 177
MF156584.1  GAYARARVGIHTAVRVPPTGSPTHTHLRQDPGDEPTSDDSGLYPLDARALAHLVMLPADH 177
LT594110.1  GAYARARVGIHTAVRVPPTGSPTHTHLRQDPGDEPTSDDSGLYPLDARALAHLVMLPADH 177
KJ847330.1  GAYARARVGIHTAVRVPPTGSPTHTHLRQDPGDEPTSDDSGLYPLDARALAHLVMLPADH 177
JQ780693.1  GAYARARVGIHTAVRVPPTGSPTHTHLRQDPGDEPTSDDSGLYPLDARALAHLVMLPADH 177
KT887224.1  GAYARARVGIHTAVRVPPTGSPTHTHLRQDPGDEPTSDDSGLYPLDARALAHLVMLPADH 177
KT780616.1  GAYARARVGIHTAVRVPPTGSPTHTHLRQDPGDEPTSDDSGLYPLDARALAHLVMLPADH 177
MH480513.1  GAYARARVGIHTAVRVPPTGSPTHTHLRHDPGDEPTSDDSGLYPLDARALAHLVMLPADH 177
            ****************************:*******************************

Strain_17_S RAFFRTVVEVSRMCAANVRDPPPPATGAMLGRHARLVHTQWLRANQETSPLWPWRTAAIN 240
HM585508.2  RAFFRTVVEVSRMCAANVRDPPPPATGAMLGRHARLVHTQWLRANQETSPLWPWRTAAIN 237
KT425110.1  RAFFRTVVEVSRMCAANVRDPPPPATGAMLGRHARLVHTQWLRANQETSPLWPWRTAAIN 237
MF156584.1  RAFFRTVVEVSRMCAANVRDPPPPATGAMLGRHARLVHTQWLRANQETSPLWPWRTAAIN 237
LT594110.1  RAFFRTVVEVSRMCAANVRDPPPPATGAMLGRHARLVHTQWLRANQETSPLWPWRTAAIN 237
KJ847330.1  RAFFRTVVEVSRMCAANVRDPPPPATGAMLGRHARLVHTQWLRANQETSPLWPWRTAAIN 237
JQ780693.1  RAFFRTVVEVSRMCAANVRDPPPPATGAMLGRHARLVHTQWLRANQETSPLWPWRTAAIN 237
KT887224.1  RAFFRTVVEVSRMCAANVRDPPPPATGAMLGRHARLVHTQWLRANQETSPLWPWRTAAIN 237
KT780616.1  RAFFRTVVEVSRMCAANVRDPPPPATGAMLGRHARLVHTQWLRANQETSPLWPWRTAAIN 237
MH480513.1  RAFFRTVVEVSRMCAANVRDPPPPATGAMLGRHARLVHTQWLRANQETSPLWPWRTAAIN 237
            ************************************************************

Strain_17_S FITTMAPRVQTHRHMHDLLMACAFWCCLTHASTCSYAGLYSTHCLHLFGAFGCGDPALTP 300
HM585508.2  FITTMAPRVQTHRHMHDLLMACAFWCCLTHASTCSYAGLYSTHCLHLFGAFGCGDPALTP 297
KT425110.1  FITTMAPRVQTHRHMHDLLMACAFWCCLTHASTCSYAGLYSTHCLHLFGAFGCGDPALTP 297
MF156584.1  FITTMAPRVQTHRHMHDLLMACAFWCCLTHASTCSYAGLYSTHCLHLFGAFGCGDPALTP 297
LT594110.1  FITTMAPRVQTHRHMHDLLMACAFWCCLTHASTCSYAGLYSTHCLHLFGAFGCGDPALTP 297
KJ847330.1  FITTMAPRVQTHRHMHDLLMACAFWCCLTHASTCSYAGLYSTHCLHLFGAFGCGDPALTP 297
JQ780693.1  FITTMAPRVQTHRHMHDLLMACAFWCCLTHASTCSYAGLYSTHCLHLFGAFGCGDPALTP 297
KT887224.1  FITTMAPRVQTHRHMHDLLMACAFWCCLTHASTCSYAGLYSTHCLHLFGAFGCGDPALTP 297
KT780616.1  FITTMAPRVQTHRHMHDLLMACAFWCCLTHASTCSYAGLYSTHCLHLFGAFGCGDPALTP 297
MH480513.1  FITTMAPRVQTHRHMHDLLMACAFWCCLTHASTCSYAGLYSTHCLHLFGAFGCGDPALTP 297
            ************************************************************

Strain_17_S PLC      303
HM585508.2  PLC      300
KT425110.1  PLC      300
MF156584.1  PLC      300
LT594110.1  PLC      300
KJ847330.1  PLC      300
JQ780693.1  PLC      300
KT887224.1  PLC      300
KT780616.1  PLC      300
MH480513.1  PLC      300
            ***
```

C

```
Strain_17_S MIKRRGNVEIRVYYESVRTLRSRSHLKPSDRQQSPGHRVFPGSPGFRDHPENLGNPEYRE 60
MH999847.1  MIKRRGNVEIRVYYESVRTLRSRSHLKPSDRQQSPGHRVFPGSPGFRDHPENLGNPEYRE 60
ON960059.1  MIKRRGNVEIRVYYESVRTLRSRSHLKPSDRQQSPGHRVFPGSPGFRDHPENLGNPEYR- 59
LT594106.1  MIKRRGNVEIRVYYESVRTLRSRSHLKPSDRQQSPGHRVFPGSPGFRDHPENLGNPEYRE 60
            ***********************************************************

Strain_17_S LPETPGYRVTPGIHDNP---GSPGLPGSPGPHAPPANHVRLAGLYSPGKYAPLASPDPFS 117
MH999847.1  LPETPGYRVTP---------GSPGLPGSPGPHAPPANHVRLAGLYSPGKYAPLASPDPFS 111
ON960059.1  --------VTPGIHDNPGLPGSPGLPGSPGPHAPPANHVRLAGLYSPGKYAPLASPDPFS 111
LT594106.1  LPETPGYRVTP---------GIHDNPGSPGPHAPPANHVRLAGLYSPGKYAPLASPDPFS 111
                    ***         *  . ***********************************

Strain_17_S PQHGAYARARVGIHTAVRVPPTGSPTHTHLRQDPGDEPTSDDSGLYPLDARALAHLVMLP 177
MH999847.1  PQDGAYARARVGLHTAVRVPPTGSPTHTHLRHDPGDEPTSDDSGLYPLDARALAHLVMLP 171
ON960059.1  PQDGAYARARVGIHTAVRVPPTGSPTHTHLRHDPGDEPTSDDSGLYPLDARALAHLVMLP 171
LT594106.1  PQDGAYARARVGLHTAVRVPPTGSPTHTHLRHDPGDEPTSDDSGLYPLDARALAHLVMLP 171
            **.*********:******************:****************************

Strain_17_S ADHRAFFRTVVEVSRMCAANVRDPPPPATGAMLGRHARLVHTQWLRANQETSPLWPWRTA 237
MH999847.1  ADHRAFFRTVVEVSRMCAANVRDPPPPATGAMLGRHARLVHTQWLRANQETSPLWPWRTA 231
ON960059.1  ADHRAFFRTVVEVSRMCAANVRDPPPPATGAMLGRHARLVHTQWLRANQETSPLWPWRTA 231
LT594106.1  ADHRAFFRTVVDVSRMCAANVRDPPPPATGAMLGRHARLVHTQWLRANQETSPLWPWRTA 231
            ***********:************************************************

Strain_17_S AINFITTMAPRVQTHRHMHDLLMACAFWCCLTHASTCSYAGLYSTHCLHLFGAFGCGDPA 297
MH999847.1  AINFITTMAPRVQTHRHMHDLLMACAFWCCLTHASTCSYAGLYSTHCLHLFGAFGCGDPA 291
ON960059.1  AINFITTMAPRVQTHRHMHDLLMACAFWCCLTHASTCSYAGLYSTHCLHLFGAFGCGAPA 291
LT594106.1  AINFITTMAPRVQTHRHMHDLLMACAFWCCLTHASTCSYAGLYSTHCLHLFGAFGCGDPA 291
            ********************************************************* **

Strain_17_S LTPPLC------   303
MH999847.1  LTPPLC------   297
ON960059.1  LTPPLCQGNLYP   303
LT594106.1  LTPPLC------   297
            ******
```

Figure S5 (Legend on next page)

Figure S5. **The Alignment of US10 Amino Acid Sequence with Different Lengths Compared to Mature US10 Protein in Strain 17.** (A) Alignment of the US10 amino acid sequence of different lengths with mature US10 in HSV-1 strain 17: HSV-N-7 (representative of 18-nt shorter group), ES (representative of 9-nt shorter group), OP297870.1 (9-nt longer), OQ102003.1 (18-nt longer) and HM585510.2 (27-nt longer). The peptide shaded with red represented intron region if the introns were not spliced. If the proposed introns were spliced, these peptide sequences would not be present and US10 protein are the same as US10 in HSV-1 strain 17. (B) Alignment of the US10 amino acid sequences of 36-nt shorter group with mature US10 in HSV-1 strain 17. (C) Alignment of the US10 amino acid sequences of 45-nt shorter group with mature US10 in HSV-1 strain 17

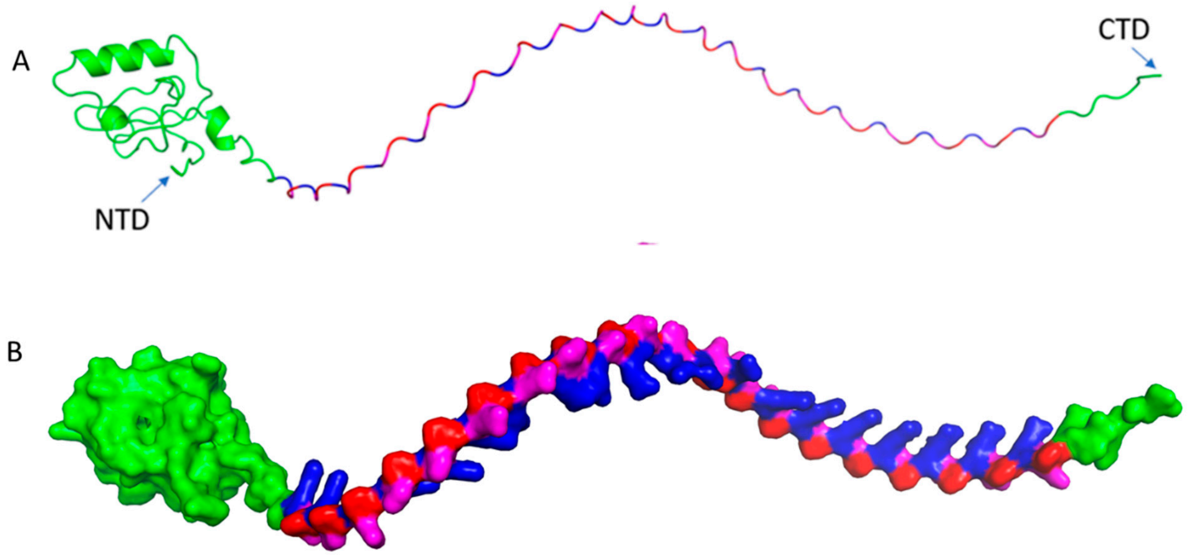

Figure S6. Predicted Structure of Mature US11 in HSV-1 Strain 17. (**A**) In cartoon mode. (**B**) In surface mode. The RXP repeats located from amino acid 86 to 145 are colored in blue for arginine, magenta for X (middle of RXP) and red for proline. Other parts are colored in green.
